# Gene network analysis predicts the primary regulators of ABA-dependent transcriptional activation and repression in *Populus* roots

**DOI:** 10.1371/journal.pgen.1012256

**Published:** 2026-08-04

**Authors:** David Cohen, Maira De Freitas Pereira, Iva Pavlović, Ondrej Novák, Marie-Béatrice Bogeat-Triboulot, Sarah Jane Cookson, Irène Hummel

**Affiliations:** 1 AgroParisTech, INRAE, UMR Silva, Université de Lorraine, Nancy, France; 2 Laboratory of Growth Regulators, Faculty of Science of Palacký University & Institute of Experimental Botany of the Czech Academy of Sciences, Olomouc, Czech Republic; 3 EGFV, Université Bordeaux, Bordeaux Sciences Agro, INRAE, ISVV, Villenave d’Ornon, France; Max Planck Institute of Molecular Plant Physiology: Max-Planck-Institut fur molekulare Pflanzenphysiologie, GERMANY

## Abstract

Abscisic acid (ABA) acts as a key signalling molecule that mediates plant responses to environmental cues as well as plant growth and development. Stress-induced and developmental changes in ABA content trigger a myriad of post-transcriptional and transcriptional events. Yet, ABA-dependent transcriptional responses are context dependent, and their temporal dynamics in roots under non-stress conditions remain poorly resolved. In this study, we characterised the hallmarks of ABA signalling and responses in the poplar root transcriptome (*Populus nigra* L.). We disturbed ABA homeostasis by exogenous ABA treatments, and we combined time-resolved transcriptomics with unsupervised gene network analysis to identify ABA-activated and ABA-inactivated gene co-expression modules. Considering the temporal dynamics of transcriptional events, we predicted the primary targets of the ABA signal, characterised early responding ABA-dependent processes, identified hub genes and revealed their putative functional links. We demonstrated that the properties of a master ABA-activated module induced by exogenous treatments were preserved in the transcriptome response to osmotic stress, revealing a core gene set of ABA-dependent stress responses. Our work sheds light on ABA repression of gene expression, the reprogramming of metabolism and the leaf-senescence pathway. Based on current functional knowledge and phylogenetic information, including poplar-specific features, we proposed a working model of ABA action on root transcriptome in poplar that integrates master genes, key responsive processes, and their putative regulatory architecture.

## Author summary

Abscisic acid (ABA) is a plant hormone that dynamically governs plant development and stress responses. Core ABA signalling pathway conveys environmental and developmental cues to the nucleus, triggering extensive transcriptional reprogramming required to adjust plant physiology. Here, we quantified time-dependent changes in gene expression and in hormone concentrations, occurring within the root of poplar plants following exogenous ABA treatments. By combining time-resolved transcriptomics with unsupervised gene network analysis, we revealed the modular structure of ABA-dependant transcriptional responses and identified the ABA-activated and ABA-repressed processes. Through the integration of gene network architecture with in-depth literature analysis and phylogenetic information, we inferred regulatory mechanisms controlling primary ABA-dependent responses in roots, and identified lineage-specific programs. Interestingly, ABA signalling in roots engages regulatory pathways and functional responses previously associated with germination, stress responses, metabolic regulation, or leaf senescence. By integrating these multiple insights into a working model, we propose a unified framework for ABA-dependent regulatory mechanisms, providing new perspectives for research in both annual and perennial plant species.

## Introduction

The sesquiterpenoid phytohormone abscisic acid (ABA) is involved in the regulation of plant development, growth and functioning. ABA signalling plays key roles throughout the plant life cycle, from seed maturation to leaf senescence, also mediating both seed and bud dormancy [1–4]. As a stress signalling molecule, ABA orchestrates plant responses to challenging environments, including water deprivation and high salinity [5–8].

Local active ABA levels are finely tuned through the balance between *de novo* ABA biosynthesis and catabolism, compartmentation, transport and external uptake. Currently, the metabolic pathways underlying ABA homeostasis are mostly well known [9,10]. ABA biosynthesis begins in the plastids via the methylerythritol phosphate (MEP) pathway, which produces isoprenoid precursors for the biosynthesis of carotenoids, gibberellin, and cytokinin [11]. The plastid-localized cleavage of 9’-cis-neoxanthin or 9-cis-violaxanthin into xanthoxin by 9-*CIS*-EPOXYCAROTENOID DIOXYGENASE (NCED) initiates the entry into the ABA biosynthetic pathway, which is completed by two cytosolic enzymatic reactions converting xanthoxin into active ABA [12]. NCED is considered to be the rate-limiting step in *de novo* ABA biosynthesis [13,14]. ABA catabolism requires the ABA hydroxylation by the CYP707A subfamily of the cytochrome P450 monooxygenases [15,16]. CYP707As mainly produce 8’-hydroxy-ABA, which spontaneously isomerizes to phaseic acid (PA). Phaseic acid reductase (PAR) converts PA to dihydrophaseic acid (DPA). While DPA is an inactive form, PA retains residual activity and can bind to the ABA receptor and activate some of the ABA-responsive genes [17,18]. CYP707As also produce 9’-hydroxy-ABA, which is converted to neophasic acid (neoPA) by neoPA reductase, NeoPAR [19]. The enzymes involved in the production of 7’-hydroxy-ABA (7-OH-ABA) have yet to be characterized. Local ABA levels depend on the diffusion of uncharged ABA forms and the active translocation of ABA [2,20]. Several proteins have ABA transport activity, such as ATP-binding cassette (ABCG) transporters, multidrug and toxic compound extrusion (MATE)-type/DTX transporters, and members of the NRT1/PTR family (NPF) [21–26]. The AWPM-19 family protein, OsPM1, has been shown to mediate ABA influx in rice [27].

The schematic model of the ABA signalling pathway was established two decades ago using exogenous application of chemicals, forward and reverse genetics, protein-protein interactions, and modelling [28–31]. Core ABA signalling components include the REGULATORY COMPONENT OF ABA RECEPTOR (RCAR), CLADE A PROTEIN PHOSPHATASE (HAB), and SNF1-RELATED PROTEIN KINASE 2 (SNRK2) [3,28,32,33]. HAB PHOSPHATASES act as a negative regulator of ABA signalling by inhibiting the kinase activity of SNRK2s, thereby repressing the phosphorylation of downstream ABA effectors. RCAR receptors, when linked to ABA molecule, bind to and inhibit HABs, releasing SnRK2s from inhibition, which in turn mediates ABA signal to specific targets, such as ABA-responsive transcription factors or membrane channels [34–38]. SNRK2 phosphorylation targets the basic leucine zipper (bZIP) transcription factors, DPBF/ABI5 (DC3 PROMOTER BINDING FACTOR) and AREB/ABF members (ABA-RESPONSIVE ELEMENT BINDING), which in turn promote the transcription of themselves and their specific targets [39,40]. These transcription factors are identified as canonical ABA pathway regulators [41]. They recognize the *cis* ABA-responsive elements (ABREs) in the promoter of their targets and activate their expression [39]. Core ABA signalling components and canonical ABA pathway regulators are highly conserved across land plants [42,43]. The physical interactions between core ABA signalling components are functionally preserved in several species, including *Populus* species [38,44,45].

Activation of ABA signalling pathway elicits massive transcriptome remodelling, and this response is strongly context-dependent [10,46]. In Arabidopsis seedlings, thousands of genes have been identified as ABA responsive, displaying pronounced increases or decreases in expression following ABA treatment [47–50]. ABA orchestrates the expression of numerous drought-responsive genes, including those involved in detoxification, protection against cellular damage, water transport, and the biosynthesis of osmoprotectants [48,51,52]. Concurrently, ABA signalling triggers transcriptional feedback loops that modulate ABA action by targeting ABA metabolism, core signalling components, and canonical regulators of the ABA pathway [10,41,47]. ABA modulates gene regulatory networks by altering both the expression and binding patterns of multiple transcription factors, and thereby influencing the transcription of their downstream targets [41,50,53]. The complex, multi-layered, and dynamic architecture of ABA-dependent transcriptional responses is widely recognised, but remains largely overlooked in roots. This is an important lack of knowledge because ABA acts as a key regulator of root growth and development, and controls the whole-plant functioning, particularly under stress conditions.

Here, we used time-resolved transcriptomics and co-expression network analysis to construct a comprehensive map of the earliest ABA-responsive events occurring in the root transcriptome of the perennial species, *Populus nigra*. We hypothesized that time-resolved ABA treatments, followed by their release, would identify the cascade of ABA-dependent responses and their underlying regulatory networks. We took advantage of the adventitious rooting capacities of clonal poplar cuttings in hydroponics to analyse the root transcriptome in the absence of leaves and under complete darkness [54,55]. Specifically, we asked which genes are potentially primary ABA targets, what is the relative importance of transcriptional activation and repression, and how are these responses coordinated over time. We investigated the preservation of ABA-dependent co-expression modules in the context of osmotic stress. Following the principles of “guilt-by-profiling” and “guilt-by-association”, we deciphered ABA regulatory network in the poplar root tip. The architecture of ABA-dependent network and its integration with multiple stress-responsive pathways in a perennial species revealed ABA-specific and lineage-specific responses.

## Results

### Abscisic acid (ABA) exposure progressively disrupts ABA homeostasis in the root tip

**Fig 1:**
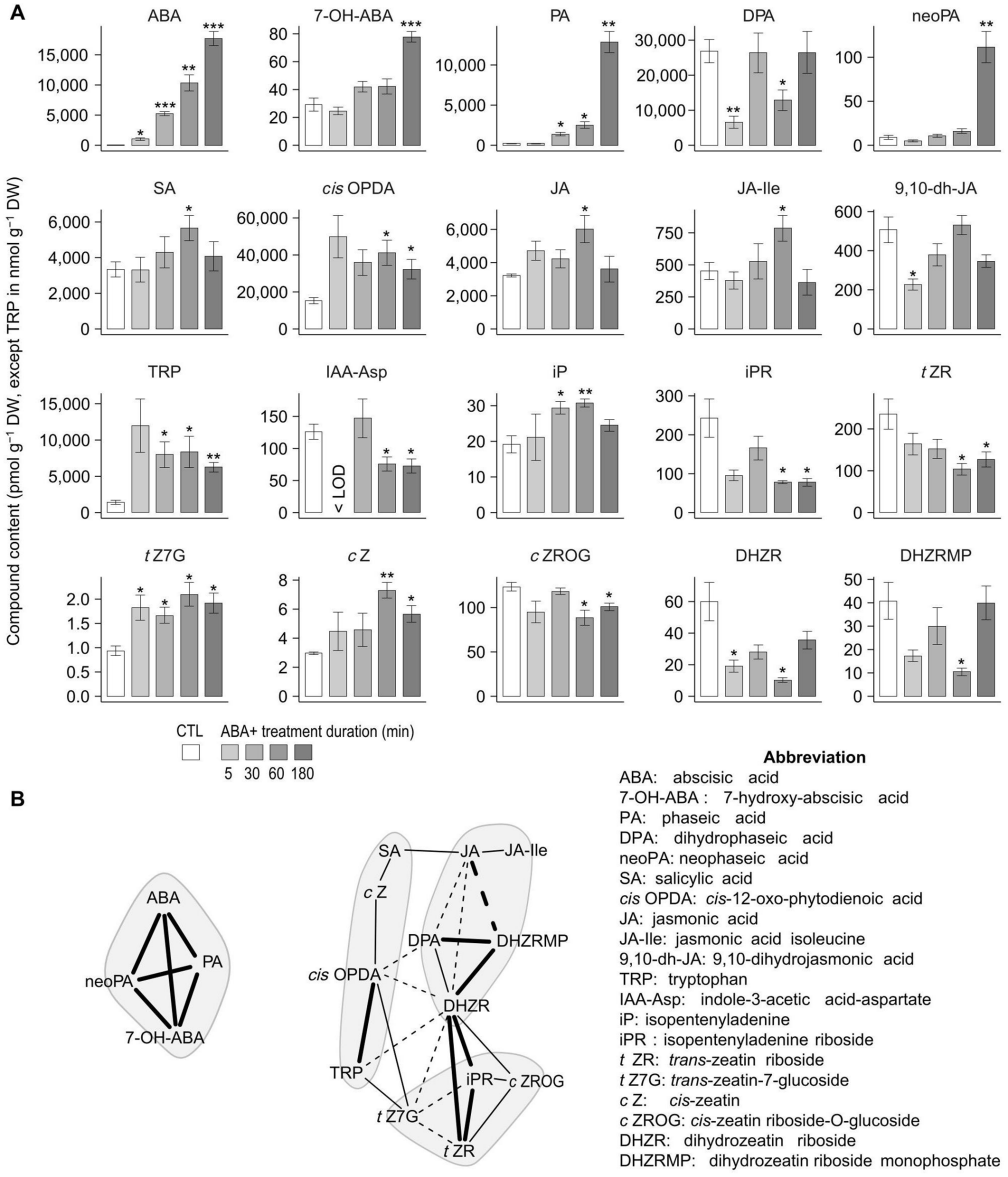
Impact of ABA treatment on hormone contents. Hormone contents were quantified in roots during Exp1, under ABA-free conditions (CTL) and after 2 μM ABA+ treatment for 5, 30, 60 or 180 min. **(A)** Hormone contents are presented as a function of treatments. Data are means ± SE, n = 4. Asterisks indicate significant differences, as determined by Student’s t-test between ABA+ versus CTL (*P*-value: *** ≤ 0.001, ** ≤ 0.01, * ≤ 0.05). “<LOD” indicates value below the limit of detection. **(B)** The covariation of hormone contents is given as a correlation network (n = 20). Only the most significant Pearson coefficients are shown (*P*-value ≤0.01). Edge width denotes the statistical significance (thick line: *P*-value ≤ 0.001). Edge type denotes the direction of correlation (dash: negative coefficient, solid: positive coefficient). Grey shapes highlight the communities which were detected via optimizing modularity score (“i.graph” R package, “cluster_fast_greedy” function with default parameters).

ABA enters plant tissues either passively by diffusion or actively by transporter-mediated uptake [20]. Poplar adventitious roots were exposed to exogenous 2 µM ABA treatment (ABA+), and the changes in the contents of ABA and ABA catabolites were monitored in the root tip over the first three hours (Exp1, Fig 1, S1 Data). Under ABA-free conditions (CTL), ABA content per gram of root dry weight was in the picomole range. The content of ABA catabolites, namely 7-OH-ABA, PA, and neoPA, was also in the picomole range while DPA was found as the major ABA catabolite, reaching the nanomole range. Under ABA+, the ABA content in the root reached the nanomole range. A 5-min-long ABA exposure was sufficient to induce a 24-fold increase in ABA content in comparison with the CTL. And a 180-min-long exposure led to a 400-fold increase in ABA content relative to the CTL. Among ABA catabolites, PA exhibited the strongest response to ABA+, reaching the nanomole range 30 min after treatment began. DPA content oscillated and was significantly reduced by 5 and 60-min-long exposures relative to the CTL. NeoPA and 7-OH-ABA underwent delayed accumulation; their contents being 10-fold and 2-fold-higher 180 min after treatment began relative to the CTL, respectively. In addition, pairwise correlation among samples revealed high positive correlations between ABA and its catabolites, except DPA (Fig 1B: ABA vs 7-OH-ABA r = 0.85***, ABA vs PA r = 0.91***, ABA vs neoPA r = 0.85***). These ABA catabolites showed a delayed accumulation relative to ABA.

In addition to ABA and ABA catabolites, 29 hormone-related compounds were quantified in the root tip under CTL and ABA+ (Fig 1, S1 Data). Half of them exhibited no significant changes in concentration under ABA+ compared with the CTL. Salicylic acid, jasmonic acid, and jasmonic acid isoleucine transiently accumulated 60 min after treatment began. The content of *cis*-12-oxo-phytodienoic acid, a precursor of jasmonate, approximately doubled under ABA+ while the content of 9,10-dihydrojasmonic acid transiently halved. Tryptophan content increased by 5-fold and the content of indole-3-acetic acid-aspartate decreased by 1.5-fold under ABA+. The contents of active cytokinin forms, isopentenyladenine and *cis*-zeatin, peaked 60 min after treatment began. The contents of isopentenyladenine riboside, *trans*-zeatin riboside, dihydrozeatin riboside and dihydrozeatin riboside monophosphate decreased under ABA+. *Cis*-zeatin riboside-O-glucoside also decreased. In contrast, *trans*-zeatin-7-glucoside accumulated under ABA+ relative to the CTL. Pairwise comparison among samples revealed some correlations between these compounds (Fig 1B). Yet, their differential accumulation did not reflect ABA+ exposure duration. Within the first three hours, ABA+ triggered the accumulation of ABA and ABA catabolites (PA, neoPA and 7-OH-ABA), which were clearly insulated from other hormone-related compounds (Fig 1B).

### ABFs and ABI5 identified as potential orchestrators of early transcriptional events

**Fig 2:**
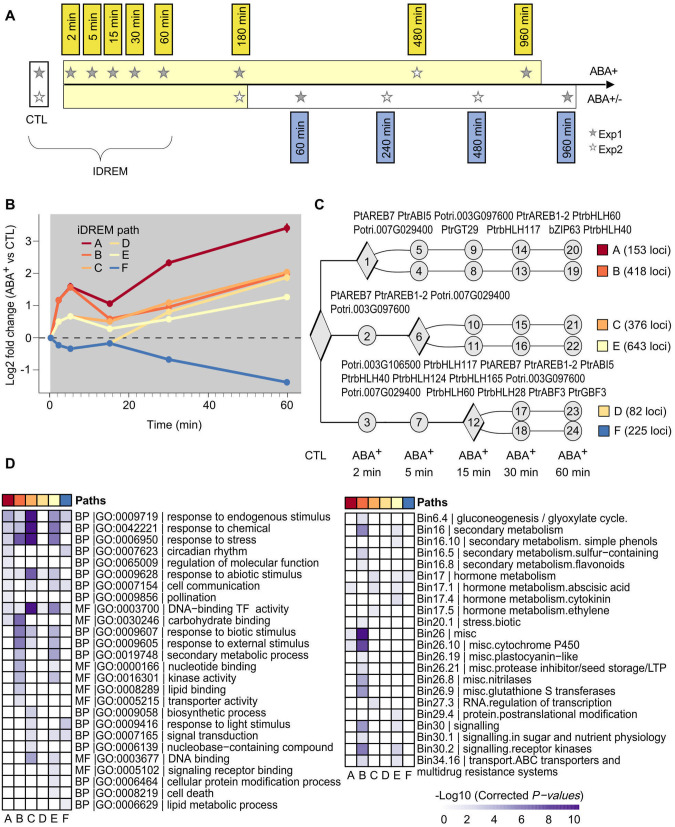
Dynamics of root transcriptome response to ABA exposure. **(A)** Experimental design for time-resolved transcriptomics. **(B-D)** iDREM revealed the transcriptional events occurring during the first hour of ABA treatment in Exp1. **(B)** iDREM reconstructed six distinct patterns of temporal expression (paths A to F). **(C)** iDREM regulatory map annotated with the putative transcription factors most likely to regulate the path transition events (Diamond, split cut-off score <0.0001). **(D)** Gene ontology (GO) and MapMan (Bin) enrichment analyses for the six iDREM paths are given as an heatmap with darkest colors being associated with the most significant enrichment.

ABA triggered transcriptional remodelling within the first hour of exposure (Exp1: CTL and ABA+ for 2 min to 60 min, Fig 2). iDREM, interactive Dynamic Regulatory Events Miner [56], reconstructed the temporal dynamics of early transcriptional events upon ABA+ and revealed the putative transcription factors most likely to control them (Fig 2, S2 Data). ABA exposure triggered six patterns of temporal expression (path A to F gathering 1,897 loci, Fig 2). The earliest event was the split between path A and path B, which occurred between 2 and 5 min after treatment began (Fig 2C). Path A corresponded to the loci with the strongest transcriptional activation (Fig 2B). Moderately responsive to ABA+, path C and path E bifurcated between 5 and 15 min after treatment began and then their expression increased in parallel with path B. The divergence between path D and path F occurred later, between 15 and 30 min after treatment began, but was more drastic (repression for path F vs activation for path D). The six transcriptional time-course profiles led to four distinct transcriptional states after a 60 min-long ABA+ exposure: 153 loci with highly up-regulated expression (path A), 876 with moderately up-regulated expression (path: B, C and D), 643 loci with weakly up-regulated expression (path E) and 225 loci with down-regulated expression (path F). Enrichments of the Gene Ontology (GO) terms and MapMan bins in the loci of the different paths were consistent with abiotic stress responses and short-term response to ABA (Fig 2D and S2 Data). All paths were enriched in the molecular function “DNA-binding transcription factor activity” and in the biological processes related to response to stimulus, including “GO:0009719”, “GO:0006950”, “GO:0009607” and “GO:0042221”. Path B and E were also enriched in “GO: secondary metabolic process”, “bin16: secondary metabolism”, “GO: kinase activity” and “bin30.2: receptor kinase”. Paths A, C, E and F were enriched in MapMan bins related to hormone metabolism, including “bin17: hormone metabolism”, “bin17.1: abscisic acid”, “bin17.4 cytokinin” and “bin17.5 ethylene”.

iDREM predicted 16 *P. trichocarpa* putative transcription factors as the most likely to regulate the transition events (split cut-off score <0.0001, Fig 2C and S2 Data). They encompassed a Trihelix GT3 transcription factor (*PtrGT29*), a BES1/BZR1 transcription factor (*Potri.003G106500*), six basic helix–loop–helix (bHLH) transcription factors, and eight bZIP transcription factors (*bZIP63*, *PtrGBF3*, *bZIP16/bZIP68* homolog *Potri.003G097600*, *GBF1* homolog *Potri.007G029400, PtrABI5*, *PtAREB7*, *PtrABF3*, and *PtrAREB1-2*). These transcription factors were predicted to regulate the expression of 250 target genes on the regulatory map (Fig 2C and S2 Data). Among them, six transcription factors were transcriptionally activated by ABA, namely *PtrGT29* in path B*, PtrGBF3* and *PtrABF3* in path C, and *PtrbHLH165, PtrAREB1-2* and *PtrABI5* in path E. The gene regulatory network (GRN) inferred for the 16 transcription factors comprised 965 predicted transcription factor-target interactions and 258 nodes in four ABA-activated paths (A, C, D and E) (S1A Fig and S2 Data). Most of these transcription factors are homologs of known master transcription factors of ABA signalling in Arabidopsis [41,53]. AREB/ABF proteins are activators of ABA response in the early steps of ABA signalling cascade [57]. In Arabidopsis, bZIP16 inhibited ABA action during seedling development [58]. In summary, the iDREM regulatory map and GRN revealed the rapid activation of the ABA-driven signalling cascade in poplar root through the action of poplar canonical ABA pathway regulators (ABFs and ABI5).

### ABA shapes co-expression modules according to treatment duration

**Fig 3:**
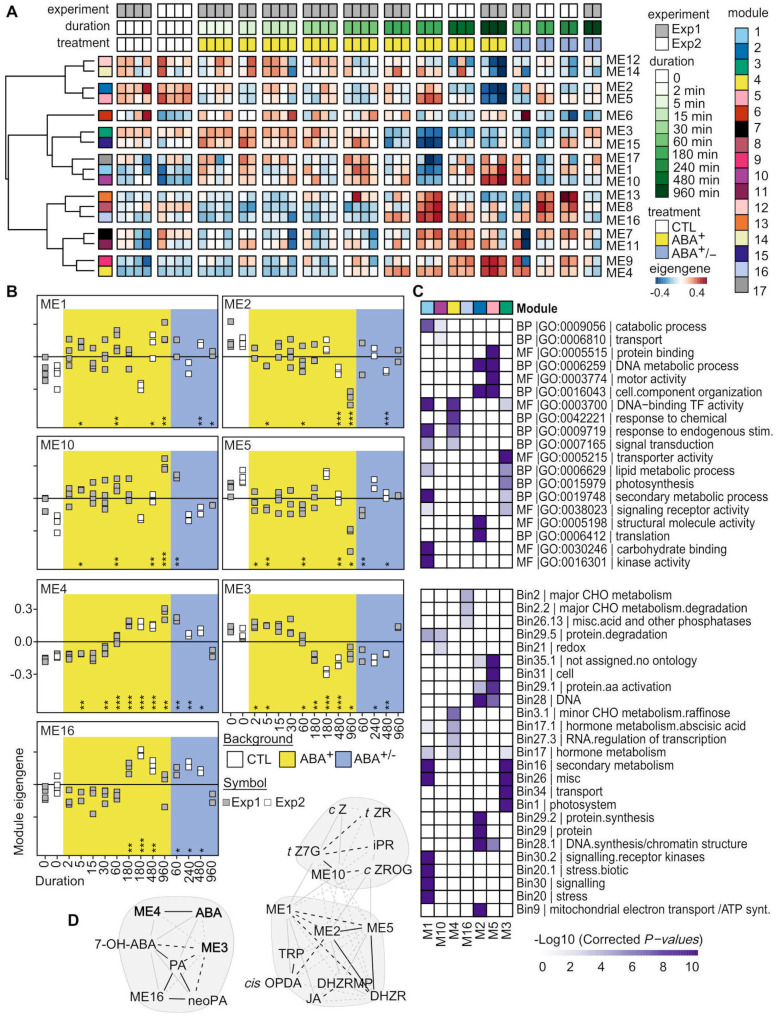
Co-expression patterns during ABA treatments. WGCNA identified 17 co-expression modules within the reference expression matrix (27,379 loci x 46 libraries). **(A)** Heatmap of module eigengene (ME) for all samples and modules. **(B)** ME are given, as a function of treatments, for seven modules significantly structured according to experimental design; Panel B shows a focused view of Panel A. Treatments are ranked according to their duration. Asterisks indicate significant differences, as determined by Student’s t-test between ABA treatments versus their respective control (*P*-value: *** ≤ 0.001, ** ≤ 0.01, * ≤ 0.05). **(C)** Gene ontology (GO) and MapMan (Bin) enrichment analyses for the seven modules are given as a heatmap with darkest colors being associated with the most significant enrichment. **(D)** Correlation network computed using the mean content of ABA responsive hormones and the mean ME of seven modules over five time points in Exp1 (Pearson correlation, n = 5, *P*-value ≤ 0.05). Edge color denotes significance level (black line: *P*-value ≤ 0.01), edge type denotes the direction of correlation (dash: negative coefficient, solid: positive coefficient), and grey shapes highlight the three communities.

The transcriptome was characterized under CTL conditions, after ABA exposure (“ABA+” for 2–960 min) and after withdrawal of ABA (“ABA+/-”, ABA+ pretreated roots transferred to CTL conditions for 60–960 min) over two experiments (Exp1 and Exp2, Fig 2A). We analysed the transcriptome data jointly using weighted correlation network analysis with a signed network type (WGCNA [59]). This WGCNA organized 24,334 loci into 17 co-expression modules (M1-17) and provided gene expression as module eigengene (ME) (Fig 3A and S3 Data). WGCNA is based on unsupervised clustering; the modules are built without considering the experimental design. Heatmap visualization of ME as a function of treatments revealed that seven modules displayed clear patterns consistent with our experimental design (Fig 3A-B). These modules showed significant ME changes in response to ABA+ treatments relative to the CTL (pairwise Student’s t-tests, *P*-value ≤ 0.01, Fig 3B). ME1, ME2, ME10 and ME5 were shaped mostly by the 960-min-ABA+ treatment, whereas ME3 and ME4, and to a lesser extent ME16, changed progressively with the duration of ABA+ treatment. The WGCNA discriminated ABA-activated modules (M1, M10, M4 and M16) and ABA-inactivated modules (M2, M5 and M3) (Fig 3A-B). Among them, three modules were significantly enriched in loci previously associated with iDREM paths (Fig 2 and S2 Data). The ABA-inactivated M3 was significantly enriched in loci associated with ABA-inactivated iDREM path F. The ABA-activated M1 was significantly enriched in loci associated with iDREM paths A, B, C and E while the ABA-activated M4 was significantly enriched in loci associated with iDREM paths A, C, D and E. Consistently, the iDREM-derived GRN mostly included genes from M1 and M4, representing 24% and 55% of the nodes, respectively (S1B Fig and S2 Data). In addition, 11 out of the 16 transcription factors acting in the first layer of the GRN were associated with the ABA-activated M4 (S1B Fig and S2 Data).

The loci within the co-expression modules were enriched in various GO terms and MapMan bins (Fig 3C, S4 and S5 Data). The ABA-activated modules M1 and M10 shared enrichments in “bin29.5: protein degradation” and “GO: catabolic process”. The ABA-activated module M4 was enriched in “bin17.1: hormone metabolism.abscisic acid”, “bin3.1: minor CHO metabolism.raffinose”, “GO: response to chemical”, and “GO: DNA-binding transcription factor activity”, while the ABA-activated module M16 was enriched in “bin2: major CHO metabolism”. The ABA-inactivated modules M2 and M5 shared enrichments in “bin28: DNA”, “GO: DNA metabolic process”, “bin29.1: protein.aa activation”, and “GO: cellular component organization”, whereas the third ABA-inactivated module, M3, was enriched in transport, lipid metabolism, secondary metabolism and photosynthesis (“GO:0005215”, “bin34”, “GO:0006629”, “GO:0019748”, “bin16”, “GO:0015979” and “bin1”). Co-expression module analysis was completed by correlating MEs with hormone changes within the first three hours of ABA exposure (Fig 3D). In this time window, nine modules were correlated with at least one hormone profile. Yet only ME3, ME4, and ME16 were correlated with ABA and ABA catabolites. Early changes in ME4 and ABA content were highly correlated (*P*-values <0.01) while ME3 and ME16 captured the delayed accumulation of ABA catabolites (Fig 3D).

**Fig 4:**
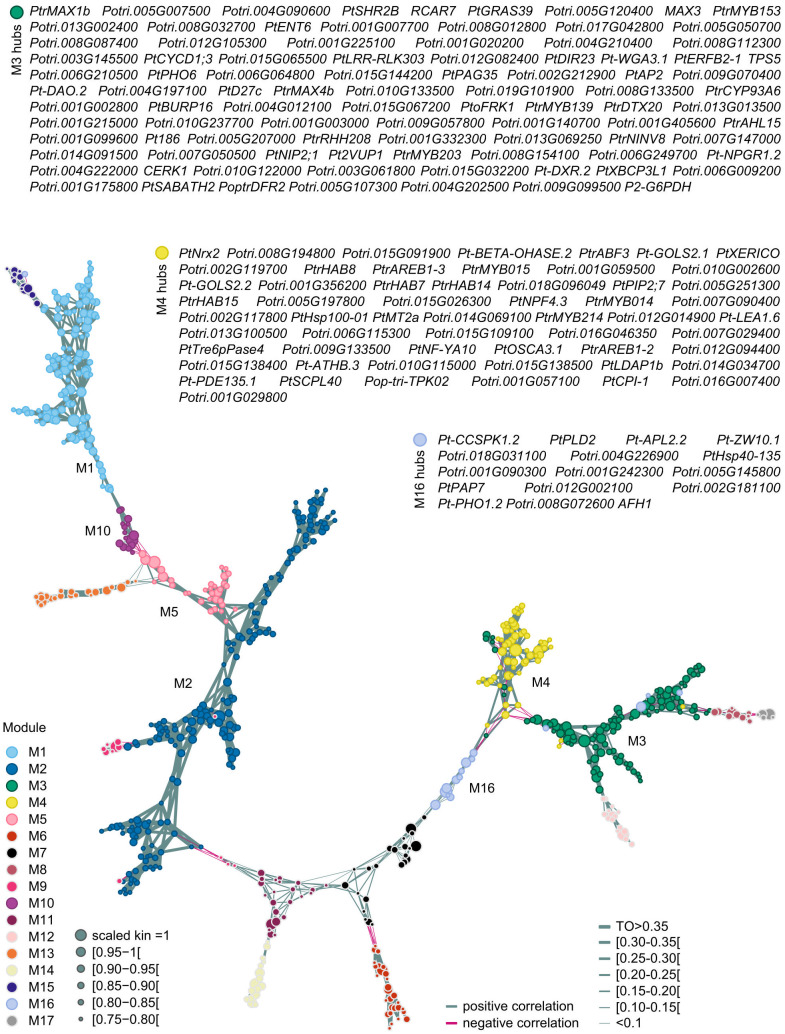
Modular structure of hub-hub co-expression during ABA treatments. WGCNA hub network was summarized as a planar correlation network. Planar network was constructed on most significant pairwise correlation (FDR < 0.05) over the 678 hub genes (scaled intramodular connectivity, Kin ≥ 0.75). Network is drawn using Fruchterman and Reingold layout. Node color and size denote module assignation and scaled Kin, respectively. Edge color and edge size denote the direction of pairwise correlation and hub-hub topological overlap, respectively. Unigene identifiers are given for M3, M4 and M16 hub genes. They are ranked in descending order of Kin. Either *P. trichocarpa V4.1* identifier or a common name is given for each unigene (detailed annotation and reference in S3 Data)**.**

WGCNA computes the intramodular connectivity (kin) that is the connectivity of a given locus with respect to other loci from the same module. This kin metric allowed us to identify 678 intramodular hubs (scaled kin ≥ 0.75). Notably, the iDREM-derived GRN included 19 intramodular hubs, most of which were associated with M4 (S1B Fig and S2 Data). These 17 M4 hubs encompassed three transcription factors acting in the first layer of the iDREM-derived GRN (*Potri.007G029400*, *PtrABF3* and *PtrAREB1*–2, S1B Fig). We investigated hub-hub co-expression relationships using a correlation-based approach and presented the most significant hub-hub correlation as a planar filtered network (Fig 4 and S6 Data). This network map highlighted the modular structure of gene co-expression. Intramodular hubs aggregated locally according to their module assignation. Moreover, intramodular hub edges exhibited a high value of topological overlap (TO). This TO metric shows the similarity of relationships between pairs of loci with respect to all other loci from the expression matrix [60]. The strongest intermodular relationships connected M2 and M5 hubs consistently with their ME covariation (Fig 3A). Intermodular hubs relationships showed the strong connection of ABA-activated and ABA-inactivated modules through highly significant and negative correlations (M3-M4, M16-M3, and M10-M5). Network layout revealed that M2, M5, M10 and M1 hubs formed a community that was distant from the community of M3, M4 and M16 hubs.

### ABA-dependent transcriptional network is partially preserved in the transcriptome response to osmotic stress

**Fig 5:**
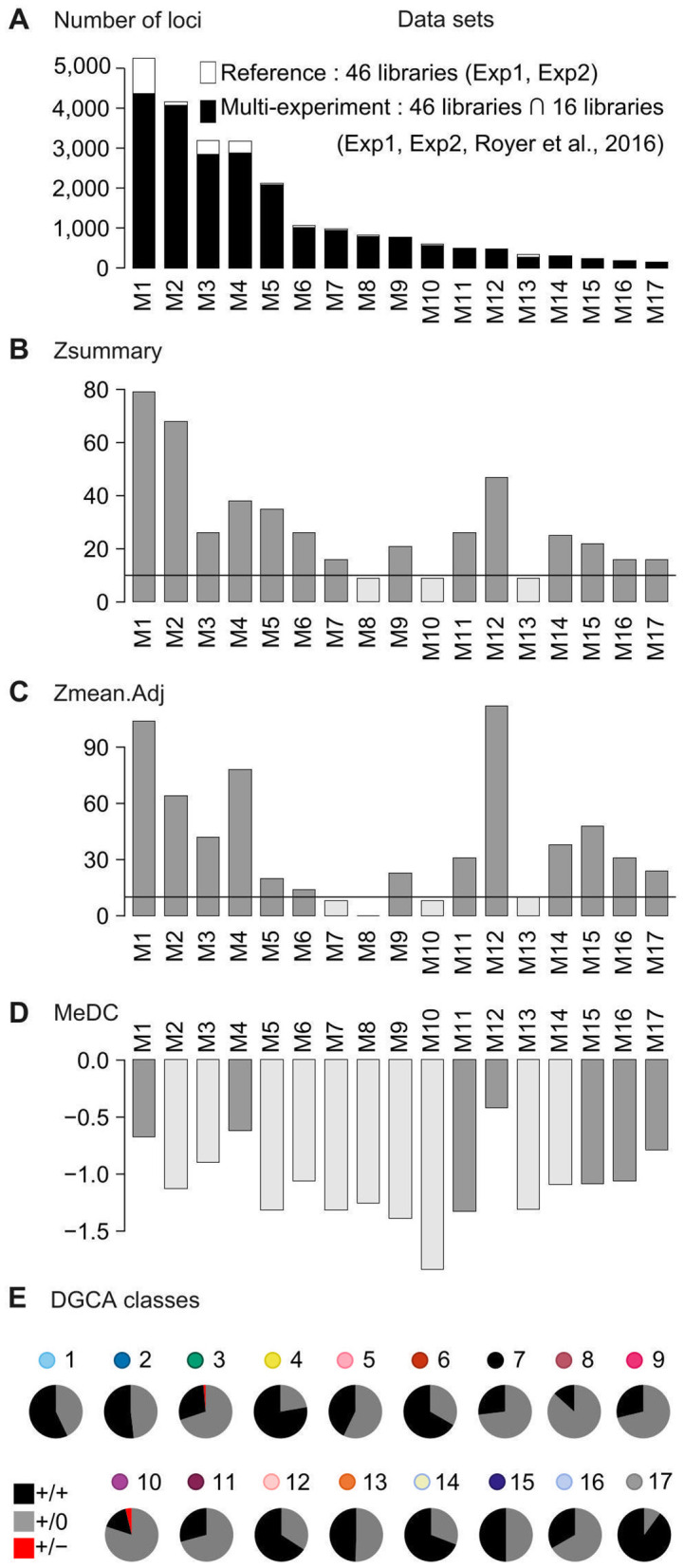
Preservation of co-expression relationships in osmotically stressed samples. The modular structure revealed during ABA treatments (reference set) was tested for its preservation during early osmotic stress response (test set = CTL or 30 min and 180 min-long exposure to a polyethylene glycol-induced osmotic potential of −0.35 MPa). **(A)** Module size is given for reference and multi-experiment datasets. **(B-C)** WGCNA preservation statistics (nPerm = 200). Modules with Zsummary metrics **(B)** or Zmean.Adj metrics **(C)** > 10 (horizontal line) are highly preserved in reference and multi-experiment datasets. Light boxes denote weak to moderate preservation. **(D-E)** DGCA metrics. **(D)** Modular average differential connectivity, MeDC, is the change in correlation among module-embedded loci in reference vs test sets. Light boxes denote MeDC significantly different from 0 (nPerm = 75, empirical *P*-value for significance <0.01). **(E)** The distribution of differential correlation classes for pairwise relationships between intramodular hub loci is given.

WGCNA provides statistics for studying the properties of network modules in an independent dataset [61]. We tested whether the modular properties triggered by ABA treatments (Fig 3) were preserved during early responses to osmotic stress [62]. The multi-experiment dataset included 24,962 loci (Fig 5A and S3 Data). Strong evidence for a high preservation of modular properties in terms of connectivity and density was found for 13 out of the 17 modules (Zsummary >10 and Zmean.Adj > 10 respectively, Fig 5B-C). As Z-scores depend on module size, Zsummary values are expected to be the highest for largest modules. Within their respective size classes, the modular structure of ABA-activated modules M1 and M4 were best preserved (Fig 5A–C). Their mean adjacency scores were significantly higher than by chance with the highest significance (M1: Zmean = 104, Log *P* = -2328; M4: Zmean = 78, Log *P* = -1306). Among the small modules (<1,000 loci), M12 exhibited the strongest preservation of modular properties (M12: Zmean = 112, Log *P* = - 2728). The preservation of modular properties highlighted the high structural robustness and strong reproducibility of ABA-activated modules M1, M4 and M16, as well as ABA-inactivated modules M2, M3 and M5, in response to exogenous ABA treatment and osmotic stress (Fig 5B–C).

Differential gene correlation analysis (DGCA) provides a bottom-up approach for assessing module preservation [63]. DGCA computes the modular average differential connectivity (MeDC) considering all module-embedded loci pairs. As shown in Fig 5D, ABA-activated modules M1, M4 and M16 exhibited no significant changes in modular connectivity between the two datasets (M1: MeDC = -0.7, *P-*value = 0.05; M4: MeDC = -0.6, *P-*value = 0.01; M16: MeDC = -1.1, *P-*value = 0.01). M12 also exhibited strong preservation of modular properties (M12: MeDC = -0.4, *P-*value = 0.5). In contrast, ABA-inactivated modules (M2, M3 and M5) and ABA-activated M10 exhibited a significant loss of average connectivity during the response to osmotic stress (*P-*value = 0, Fig 5D).

DGCA provides statistics for assessing the preservation of pairwise gene correlation between datasets [63]. We tested whether the hub-hub co-expression relationships triggered by ABA treatments were preserved under osmotic stress (S7 Data). DGCA retained 108,947 significant pairwise correlations between hubs in the multi-experiment dataset (*P-*value <0.01, S7 Data). DGCA computes correlation changes between the reference and test sets based on the mean difference in Z-score of the correlations and associated adjusted *P*-value (S7 Data). These results are summarized into nine DGCA classes in which positive, negative or non-significant correlations are coded as “+”, “-” or “0”, respectively, in both the reference and test sets (“reference/test” S7 Data). Intramodular hub-hub relationships were positive and highly significant under ABA treatments (30,226 “+/” correlations, *P*-value <0.01), and half of them were preserved under osmotic stress (DGCA class “+/+”: 15,066 correlations, *P*-value <0.01, Fig 5E). As shown in Fig 5E, the preservation of intramodular hub-hub relationships varied across modules. Among the ABA-activated and ABA-inactivated modules, M4 showed the best preservation of intramodular relationships (78% of DGCA class “+/+”). Within M1, M2 and M5, approximately half of intramodular relationships were preserved under osmotic stress. In contrast, more than 60% of intramodular relationships within M3, M10 and M16 were disrupted under osmotic stress. The divergent DGCA class “+/-” involved only 46 loci and occurred in ABA-responsive modules M3 and M10. Collectively, module-based statistics and hub-centred metrics showed that ABA-inactivated M3 and ABA-activated M10 were less preserved, and that ABA-activated M4 was the most preserved module in the context of osmotic stress.

Finally, our analysis also revealed that the expression of 17 hub genes acting in the iDREM-derived GRN was significantly up-regulated in response to both ABA treatments and osmotic stress (S1C Fig and S2 Data). Genes differentially expressed in response to osmotic stress represented 40% of the nodes, including four transcription factors acting in the first layer of the GRN. This analysis identified 85 genes whose expression rapidly increased in response to both ABA and moderate osmotic stress (S2 Data).

### Identifying the hallmarks of ABA-dependent transcriptional network

In-depth interrogation of literature and public databases outlined the biologically relevant characteristics of the ABA network. We prioritised the most central loci in the ABA-activated module M4, the ABA-inactivated module M3 and those supported by iDREM analysis (Figs 2 and 4 and S1). While prioritising the functional information obtained in poplars, we also took into account homology-based information. The phylogenetic inference of orthologs was obtained by querying comparative genomics databases [64,65]. This functionally grounded analysis highlighted seven molecular processes and pathways that are the most likely to be coordinated by ABA. They are described in detail below.

#### Transcriptional feedback loops for ABA action.

**Fig 6:**
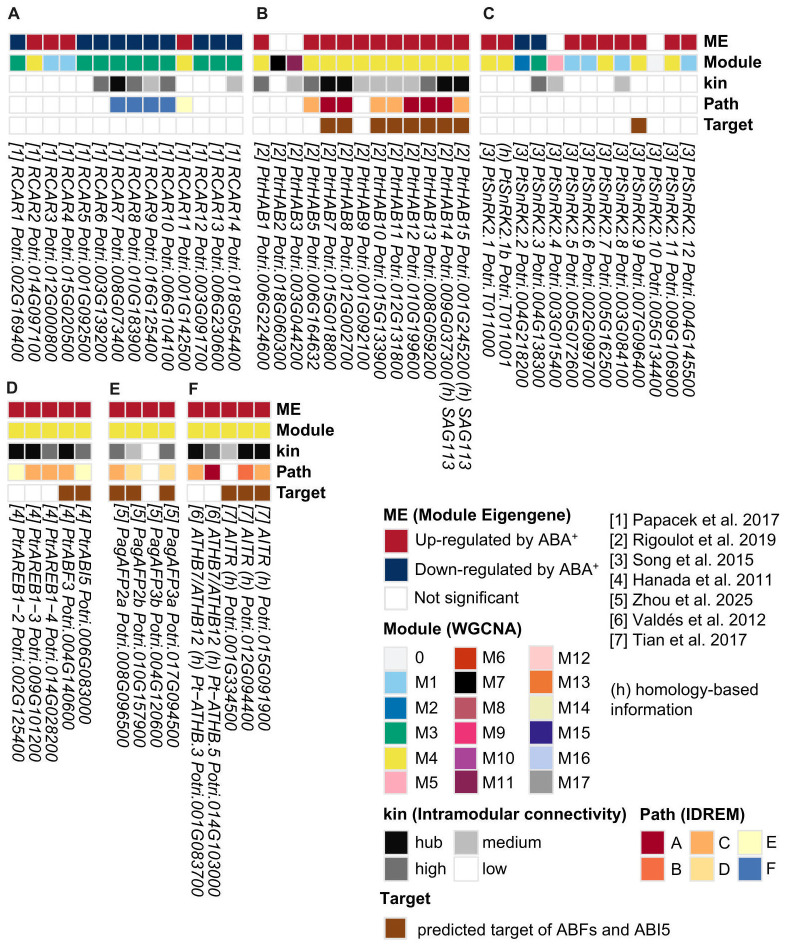
Genes involved in core ABA signalling pathway or in transcriptional feedback loops for ABA action. **(A)** Genes encoding REGULATORY COMPONENTS OF ABA RECEPTOR, RCAR. **(B)** Genes encoding CLADE A PROTEIN PHOSPHATASE, HAB. **(C)** Genes encoding SnrK2 PROTEIN KINASE. **(D)** Genes encoding canonical pathway regulators, ABFs and ABI5. **(E)** Genes encoding ABI5-BINDING PROTEINS, AFPs. **(F)** Genes encoding transcriptional regulators of ABA action. Heatmaps merge the main features from WGCNA (gene assignation to ABA-activated or ABA-inactivated modules, module assignation and scaled intramodular connectivity), from iDREM analysis (path), and from *in silico* prediction about ABI5 and/or ABFs gene interaction from plantregmap.gao-lab.org (Target). [number] indicates literature source and (h) indicates homology-based information by querying comparative genomics databases. Only unigenes expressed in roots are shown, identifiers refer to *P. trichocarpa* V4.1 genome.

Thirty-two M4 genes were associated with core ABA signalling pathway and its transcriptional regulators (Fig 6). Among these, all poplar genes encoding the canonical ABA pathway regulators (ABFs and ABI5) showed high intramodular connectivity in ABA-activated M4 (*PtrAREB1-3*, *PtrAREB1-4*, *PtrABF3*, *PtrAREB1-2*, and *PtrABI5*). One-third of the genes harbouring predicted motifs for ABF and ABI5 binding sites were found in the ABA-activated M4 (18 hub genes and 411 genes, S3 Data). A feedback loop regulating core ABA signalling pathway involves the interaction of ABFs and ABI5 with ABI5-BINDING PROTEINS (AFPs [66–68]). In poplar, PtrAREBs activate *PagAFP2a* expression and PtrAREBs-PagAFP2 interactions repress the expression of PtrAREB targets [68]. This feedback loop was ABA-activated in the root tip (Fig 6E). On the other hand, a negative transcriptional feedback acts on core ABA signalling components by modulating the expression of ABA receptor (*RCARs*) and the inhibitory protein phosphatase (*HABs*). As in Arabidopsis, ABA triggered the opposite regulation of *RCAR* and *HAB* expression [69–71]. Ten out of the 14 *RCARs* clustered in the ABA-inactivated M3 while 11 out of the 13 *PtrHABs* clustered in the ABA-activated M4. In Arabidopsis, ABA-dependent regulation of *HABs* and *RCARs* is mediated by homeodomain-leucine zipper transcription factors (ATHB7 and ATHB12 [72]). Their homologs, *Pt-ATHB.3* and *Pt-ATHB.5*, were central in the ABA-activated M4 and are present in transcriptional networks underlying drought and stress memory [73,74]. ABA-INDUCED TRANSCRIPTION REPRESSORS (AITR) also control *RCAR* and *HAB* expression [75,76] and there was strong connectivity of the three *AITR* homologs in M4.

Twelve M4 genes were associated with the post-transcriptional regulation of the core ABA signalling pathway (S2 Fig). In Arabidopsis, ABA sensing is subjected to a post-transcriptional feedback loop involving the WD40 protein ABT (ABA SIGNALLING TERMINATOR), which disrupts the RCAR/HAB complex, and enhances HAB activity [40,77,78]. The ABT-centred feedback loop is likely to be present in poplar, as we found that the two poplar *ABT* homologs were transcriptionally activated by ABA (S2 Fig). Among the post-transcriptional regulators acting on SNRK2 phosphorylation [79], only one *EGR* phosphatase homolog exhibited a strong ABA activation (S2 Fig). Yet, a putative inhibitory loop involving SNRK2-Interacting Calcium Sensors, SCS [80], was transcriptionally activated by ABA in poplar root. Selective proteasomal degradation is known to occur for core ABA signalling components [3,81]. E3 ligases ATPUB22 and ATPUB23 are involved in the degradation of RCARs, while AIRP2 is involved in the activation of ABI5 [82–84]. The poplar homologs of *ATPUB22* and *ATPUB23* were transcriptionally activated by ABA, as well as the *AIRP2* homolog, *Potri.006G099600*. In Arabidopsis, both ABA and drought activate the expression of E3 ligases *CHYR1* and *XERICO* [85,86]. The expression of *PtCHYR1* was not modified by ABA in poplar roots, while its paralogs (*Potri.006G121801* and *PtCHYR2*) and *PtXERICO* were ABA-activated. The overexpression of *PtXERICO,* a M4 hub gene*,* triggers the expression of genes involved in the ABA homeostasis and enhances poplar drought tolerance [86]. ABA also activated the expression of *PtrPUB72* and *PtrPUB82* (S1 and S2 Figs)*.* The expression of these genes is also up-regulated in response to drought, and PtrPUB72 positively regulates ABA-dependent drought response *via* the ubiquitination of PtrWRKY77, a repressor of the ABA response [87,88].

#### Cell protection against damage.

ABA increased the expression of genes whose products are putatively involved in cell protection against damage, and we further identified 15 M4 hub genes potentially involved in cell protection (S3 Fig). These genes are also transcriptionally activated in responses to water deficit in various organs and species [41,62,73,74,89–92]. The M4 hub gene, *PtHsp100-01,* is orthologous to *DAA1*, an ABA responsive gene that encodes a putative AAA-ATPase with a chaperone-like activity in Arabidopsis [93,94]. Other M4 hub genes or highly connected genes are related to redox homeostasis, another important component of cell protection [95–98]. They included *nucleoredoxin*, *metallothionein*, *cytosolic glutathione peroxidase*, and *putative mitochondrial NDUFS4*. Meanwhile, ABA activated gene expression for late embryogenesis abundant (LEA) proteins and for enzymes involved in raffinose family oligosaccharides (RFO) metabolism (S3 Fig). RFOs and LEA proteins have stabilizing functions, including membrane protection, and act as scavengers of reactive oxygen species or as osmoprotectants [98,99]. In poplar, PtrABF3, one of the IDREM predicted transcriptional regulators, has been shown to activate the expression of several genes in M4 with potential cell protectant ability, such as the LEAs *PtrLEA55* and *RD29b* [8]. In Arabidopsis, *RD*s and *ERDs* encode several families of core drought-inducible proteins and are widely used as transcriptional drought reporters [89,100]. Here, ABA activated the expression of three *ERD7* homologs, harbouring the senescence domain “IPR009686”. In Arabidopsis, ERD7 binds to negatively charged phospholipids and remodels membrane lipid composition [101]. In both Arabidopsis and poplar, ERD7 and LEAs are present in the proteome of lipid droplets [102,103]. These endoplasmic-reticulum-derived organelles, coated with lipid droplet associated proteins (LDAPs), OLEOSINs (OLEs) and SEIPINs, proliferate during stress responses [104–106]. The genes associated with lipid droplets and triacylglycerol synthesis were mostly found in the ABA-activated modules M4 and M1 (S3 Fig). *PtLDAP1b, PtOLE5 and PtOLE6* were identified as putative targets of ABF/ABI5 (S3 Fig). Concordantly, *LDAP* expression is induced by ABA and drought in Arabidopsis [107]. The expression of *AT2G25890*, a *PtOLE6* homolog, is activated by ABF1, ABF2 and ABI5, and repressed by AFP1 and AFP2 [67,108]. The ABA-activated M4 gene *Potri.002G206100* is the ortholog of *TSPO*, an ABA-responsive gene encoding a multi-stress-inducible Translocator Protein that, in *Arabidopsis*, has been proposed to scavenge porphyrins and regulate lipid droplet formation [109–111].

#### Isoprenoid and hormone metabolism.

ABA reprogrammed the expression of genes regulating isoprenoid production *via* the MEP pathway and hormone metabolism (S4 Fig). The MEP pathway provides isoprenoid precursors for diverse pathways [11]. All enzymatic steps of the MEP pathway and its branch points were represented by at least one highly connected gene in the ABA-inactivated M3, including a hub gene encoding geranylgeranyl pyrophosphate synthase (S4A and S4B Fig). In a similar fashion to the MEP pathway, all enzymatic steps in the carotenoid biosynthesis were represented by at least one highly connected gene in the ABA-inactivated M3. For instance, in the case of *PHYTOENE SYNTHASE* (EC:2.5.1.32), although the *PSY* ortholog (*Potri.017G138900*) was present in ABA-activated M4, its paralog (*Potri.001G007700*) was a hub gene in the ABA-inactivated M3 and associated with the ABA-inactivated iDREM path F. In Arabidopsis, PSY is under the post-transcriptional control of ATOR-LIKE, which controls carotenoid biosynthesis [112,113]. Notably, the M3 hub gene *Potri.006G210500* is orthologous to ATOR-LIKE. A key gene involved in ketocarotenoid biosynthesis was an ABA-inactivated M3 hub gene (*Potri.004G197100*) encoding CAPSANTHIN/CAPSORUBIN SYNTHASE (EC:5.3.99.8) [114]. Conversely, key genes involved in zeaxanthin and ABA biosynthesis were co-expressed in the ABA-activated M4, including *β-carotene 3-hydroxylase*, *zea-epoxidase*, *NCED*, and *xanthoxin oxidase* (EC:1.14.15.24, EC:1.14.15.21, EC:1.13.11.51 and EC:1.1.1.288). In addition, the ABA-activated M4 included four putative ABA-catabolizing *CYP707*s (EC:1.14.14.137), but did not include any of the nine poplar genes that we identified as putative *PARs* and *NeoPARs* based on their homology with *CRL1*, *ABH2* and *NeoPAR1* [18,19]. Carotenoid pathway feeds strigolactone synthesis, which was massively repressed by ABA (including the M3 hub genes: *PtrMAX1b*, *MAX3*, *PtrMAX4b* and *PtD27c,* S5 Fig). ABA rewired cytokinin pathway, with down-regulation of biosynthetic genes (*PtIPT5a*, *Pt-CYP735.1* and *Pt-CYP735.2*), up-regulation of catabolic genes (seven *PtCKXs*) and differential ABA responses for other genes (S6 Fig). The ABA-inactivated M3 was enriched in “gibberellin metabolism” (S4 Data), indicating that gibberellin synthesis was down-regulated in response to ABA (S5 Fig). Three *PtGA3oxs,* encoding the enzyme that catalyses the synthesis of bioactive gibberellin, were present in M3, along with other biosynthetic genes. Six of these genes encodes 2*-*OXOGLUTARATE-DEPENDENT DIOXYGENASEs and are associated with *PtGA3ox1* in a gibberellin metabolic cluster located on the poplar chromosome 1 [115]. In support of the negative impact of ABA on the gibberellin pathway, three genes encoding gibberellin-inactivating enzymes were found in the ABA-activated M4, *PtaGA2ox6* and *PtaGA2ox1,* as well as the *ELA1/ELA2* homolog *Potri.019G064200* [116].

#### Transporters and channels.

ABA remodelled the *transporter* landscape (S7 Fig). The ABA-inactivated M3 was enriched in “transport” with 206 annotated genes (Fig 3C; S4 and S5 Data). Notably, M3 was enriched in “nitrate transport” (S7A Fig, S5 Data and S1 Appendix). M3 was also enriched in “nitrogen metabolism” and “oxidative pentose phosphate (OPP) pathway” (S8 Fig and S5 Data). As the OPP pathway provides reductant for biosynthetic reactions, including nitrogen assimilation in heterotrophic tissues [117], ABA repressed, at least at the transcriptional level, key enzymatic steps in nitrogen assimilation. For the sake of brevity, more information about aquaporins (M3 and M4 hub genes), ion channels of OSCA family (highly-connected M4 genes) and transport of anthocyanin/flavonoid (ABA-inactivated M3) are given in S1 Appendix.

#### Anthocyanin/flavonoid pathway.

ABA antagonized the anthocyanin/flavonoid pathway, decreasing the expression of genes involved in transport, metabolism, and transcription factors (S7 and S9 Figs). The ABA-inactivated M3 was enriched in “secondary metabolic process” and more specifically in “flavonoids” (Fig 3, S4 and S5 Data). Most steps in anthocyanin/flavonoid synthesis were repressed at the transcriptional level. However, ABA activated the expression of genes encoding FLAVONOL SYNTHASE, the key enzyme for flavonol production (M16, EC:1.14.11.9, S9 Fig). Such transcriptional regulations also occur in grapevine roots submitted to ABA [118]. R2R3-MYBs are key transcriptional regulators of the anthocyanin/flavonoid metabolism in poplar [119–125]. The ABA-inactivated M3 included most of MYBA1 activators, such as the hub gene *PtrMYB153,* as well as three MYB repressors, *PtrMYB179* and the paralogs *PtrMYB165/PtrMYB194* (S9 Fig). These paralogs belong to R2R3-MYB Subfamily S4, which also includes *PtrMYB203,* a M3 hub gene [126]. MYBs regulate anthocyanin/flavonoid pathway by forming an MBW complex with one bHLH and one WD40 repeat protein [127,128]. In poplar, the activators PtrMYB006 and PtrMYB115 interact with PtbHLH131, which also participates in the PtrMYB134-PtbHLH131-PtWD40 complex that activates the expression of *PoptrBAN1*, an ANTHOCYANIDIN REDUCTASE encoding gene [121,129]. These genes, except *PtrMYB134,* were well connected in the ABA-inactivated M3. In Arabidopsis and poplar, TTG1 and LTF15 are well-known MBW partners, respectively [129–132]. In Arabidopsis, MYC2 also acts as positive regulator of flavonoid biosynthesis [129,133]. The antagonistic effect of ABA on the anthocyanin/flavonoid pathway is strengthened by the co-repression of *LTF15, TTG1* ortholog (*Potri.015G002600*), and *MYC2* homologs (*PtbHLH175* and *PtbHLH178*).

#### Tetrapyrrole and chlorophyll metabolism.

ABA decreased the expression of photosystem-related genes and tetrapyrrole biosynthetic genes (Figs 3C and S10). The ABA-inactivated M3 was enriched in GO term “Photosynthesis” and in bins “photosystem”” and “tetrapyrrole synthesis” (Fig 3, S4 and S5 Data). Seventy-two genes contributed to these enrichments, but they showed low intramodular connectivity. Concerning tetrapyrrole synthesis, ABA decreased the expression of 15 M3 genes including the two genes encoding GUN4 (GENOMES UNCOUPLED 4), a regulatory subunit of Mg-chelatase, which is essential for chlorophyll accumulation [134]. In addition, we identified in the ABA-inactivated M3 two orthologs of key genes acting on chlorophyll homeostasis in Arabidopsis: *Potri.001G143400* is orthologous to *BCM1*/*BCM2* (*BALANCE OF CHLOROPHYLL METABOLISM* [135,136]) and *Potri.010G211500* is orthologous to *RPGE2*/*RPGE3* (*REPRESSOR OF PHOTOSYNTHETIC GENES* [137]). On the other hand, the ABA-activated M1 included *PtSGR1* and *Potri.001G112600*, two homologs of *SGR1*, a Mg-dechelatase degrading chlorophyll and photosystem (STAY-GREEN1, EC: 4.99.1.10, [138]). The M4 hub gene, *Potri.014G034700,* is the ortholog of *HEMD* which encodes the enzyme responsible for the formation of uroporphyrinogen III (EC: 4.2.1.75), the precursor of all tetrapyrroles [139]. *Potri.014G034700* was a predicted target of GBF1 homolog Potri*.*007G029400 on iDREM-derived GRN (S1 Fig). We identified two poplar homologs of *senescence-associated genes*, *SAG113* and *SAG201,* involved in ABA-mediated and auxin-induced leaf senescence, respectively [140,141]. Their expression was ABA-activated in poplar roots, but increased also in leaf during autumn senescence [142].

#### Other transcription factors.

ABA modified the expression of numerous transcription factors (Figs 2 and 3, S4 and S5 Data, S1 Fig). We identified 41 genes in M4 and M3 putatively involved in the amplification and the diversification of the ABA signal (S11 Fig). In Arabidopsis, ABA regulatory network highlights GT3a and GBF3 as initial response regulators [53]. Their homologs, *PtrGBF3* and *PtrGT29*, were found in the ABA-activated M4 and predicted to behave as master regulators by iDREM (Figs 2 and S1). Stress-responsive NACs, also called SNAC, and ANAC016 mediate stress responses and ABA-inducible leaf senescence [140,143–147]. Poplar *SNACs* and *ANAC016* homologs were mostly associated with the ABA-activated M4 and were secondary transcription factors on the iDREM-derived GRN (S1 Fig). ABA-responsive *SNACs* are *PtATAF1.1*/*PtATAF1.2*, *PtNAC049*/*PtNAC078* and *PtRD26*/*PtNAC105,* orthologs of *ATAF1*, *ANAC032* and *RD26/ANAC053/ANAC019*, respectively, as well as *NAC163/PtNAC118* and *NAC095*, orthologs of *ANAC047* and *ATNAP*, respectively. Most poplar *SNACs* were predicted targets of ABF/ABI5, and PtrAREB1-2 binds to the ABRE motifs of three *SNACs* (*PtATAF1.2*, *PtATAF1.1*, and *PtRD26* [148]). Meanwhile, our work confirmed the ABA-dependent transcriptional activation of R2R3-MYBs belonging to clades 16, 41 and 42, as previously reported in Arabidopsis [119,149–154]. The *R2R3-MYBs* of clade 16 comprise the ABA-activated Arabidopsis genes *MYB49, MYB41, MYB102*, and *MYB74*, and six poplar genes which were present in the ABA-activated modules M1 and M4. Among them, *PtrMYB043* is known to be transcriptionally activated by PtrABF3 [8]. In a similar fashion to the *R2R3-MYBs* of clade 16, the *R2R3-MYBs* of clade 41 and 42 comprise three ABA-activated Arabidopsis genes (*MYB121*, *MYB71*, and *MYB79*) and five poplar genes which were present in the ABA-activated M4 (S11C Fig). In a variety of species, MYB121 homologs are transcriptionally activated in roots in response to ABA or abiotic stresses [118,155–158]. We also identified ABA-inactivated transcription factors. Six M3 hub genes encoded R2R3-MYB, bHLH, AP2/ERF, bZIP and GRAS transcription factors, that, to the best of our knowledge, have not been reported yet as ABA-responsive genes (S11 Fig). Among them, *PtGRAS39* is orthologous to *SCL23* in *SCARECROW* subfamily while *PtSHR2B* belongs to *SHORTROOT* subfamily [159]. Our work identified ABA-inactivated homologs of transcription factors acting on chloroplast maintenance and chlorophyll homeostasis in Arabidopsis. *Potri.007G136901* and *Potri.017G015800* are homologous to *GLKs* (*GOLDEN2-LIKE* [136,160]). *Potri*.*006G075200* and *Potri.018G142100* are homologous to *CIA2/CIL* (*CHLOROPLAST IMPORT APPARATUS/CIA-LIKE* [161,162]).

#### Miscellaneous genes.

One-third of M4 hub genes and half of M3 hub genes, encoded miscellaneous enzymes, proteins of unknown function, or poorly documented proteins (S12 Fig). Based on our current knowledge, it is not yet possible to associate these genes with a specific process/pathway. Among them, Potri.008G194800, a DUF1338 protein encoded by a M4 hub, is orthologous to FLO7 in rice, AT1G07040 in Arabidopsis and DD15 in soybean, respectively involved in amyloplast development, lipid droplet organisation or nodule senescence [163–165]. *AT1G07040* encodes a hydroxyglutarate synthase acting in the lysine catabolic pathway [164]. Supporting its potential role in ABA responses across species, DUF1338 encoding transcripts accumulated in response to drought in poplar, Arabidopsis, rice, barley, and brome [73,91]. Although these miscellaneous genes are not functionally linked to the previously mentioned genes, their high intramodular connectivity indicates that they are strongly co-expressed in roots with well-known ABA-dependent responses.

## Discussion

**Fig 7:**
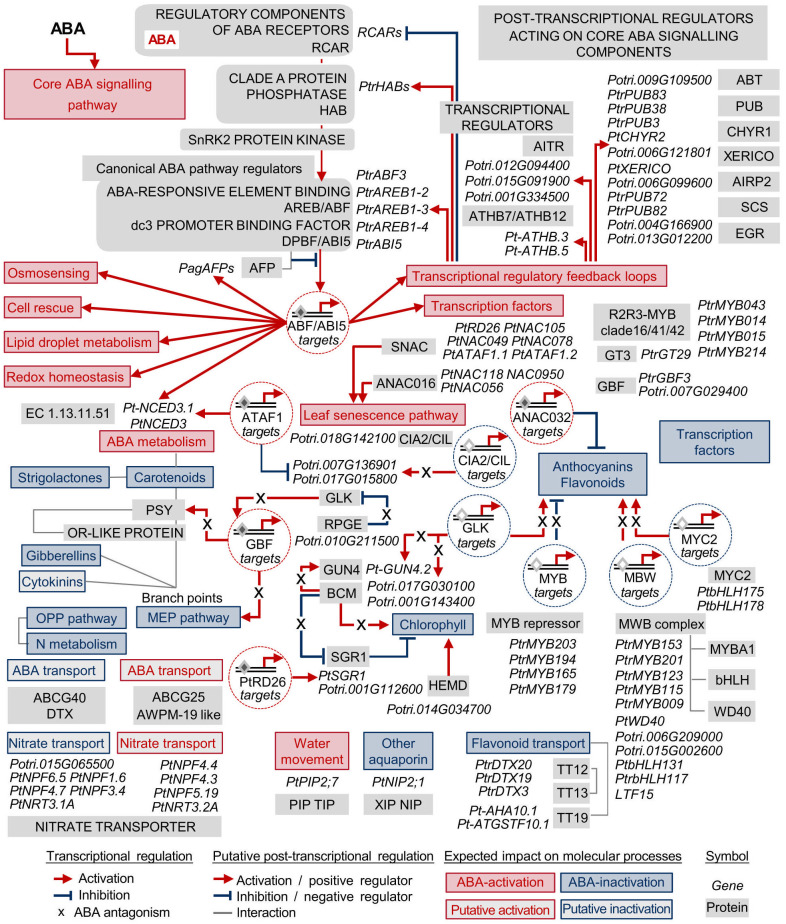
Working model of ABA primary responses in the root tip transcriptome. ABA primary responses involve the coordinated activation and repression of multiple genes, organized into co-expressed modules. These responses are rapidly triggered, showing that the ABA-dependent transcriptional network is structured over time. Our working model provides a comprehensive view of early ABA-dependent processes, their functional links and their putative regulatory architecture. ABFs and ABI5 transcription factors mediate ABA signal, and act as central nodes in ABA regulatory network. In our model, ABF/ABI5 pathway recruits SNAC and GBF transcription factors, amplifying and diversifying transcriptional responses. We propose that ABA-dependent transcriptional repression of several transcription factors, including MYB, bHLH and GLK, is central to ABA regulatory network. They coordinate and reshape downstream ABA responses, with the GLK-BCM pathway explaining the overlap between leaf senescence signalling pathway and ABA responses in the root transcriptome. For clarity, only a subset of genes is shown. The model is supported by S2-S12 Figs.

ABA orchestrates a myriad of transcriptional and post-transcriptional events during environmental changes and plant development [3,4,41]. Here, we investigated transcriptome remodelling in poplar root tips during artificial modulation of ABA homeostasis. Our biological material and experimental set-up allowed to apply short-term exogenous ABA treatments with a fine temporal resolution while controlling for well-known confounding effects in root transcriptomics. Co-expression network analysis of time-resolved transcriptomics revealed that both up- and down-regulation of gene expression contributed to the ABA responses and followed similar temporal dynamics. Both transcriptional activation and inactivation shaped the early response and subsequent transcriptome remodelling. The properties of ABA-activated modules were more robustly preserved under osmotic stress than those of ABA-inactivated modules. This may explain the emphasis placed on ABA-activated transcriptional responses in the drought literature. By considering activation and repression with equal interest, we identified the hallmarks of primary ABA responses. In Fig 7, we propose a working model integrating the processes most likely responding to ABA, their putative molecular architecture, and their regulatory links.

Following exogenous ABA treatment, core ABA signalling components and downstream ABA-dependent phosphorylation cascade were activated, consistent with their previously demonstrated functions in poplar [38,44,45]. Similar to Arabidopsis, ABI5 and ABF pathways co-operated to mediate the ABA signal across the transcriptome, triggering the transcription of specific targets [50]. ABA-dependent transcriptional regulation involves feedback loops controlling ABA action [3,10,72], and we showed that these loops, including those involving ATHB7/ATHB12, AFPs, AITRs and ABT, are conserved and transcriptionally active in poplar. Lineage-specific responses were also observed, reflecting paralog gain, loss, and neo- or sub-functionalization following genome expansion after speciation [42,166,167]. For instance, while E3 ligase CHYR1 is a single copy gene and ABA-activated in Arabidopsis [85], its poplar paralogs, but not its ortholog, were transcriptionally activated by ABA, suggesting evolutionary divergence in promoter sequences. WGCNA identified a module of co-expressed genes rapidly induced by ABA, whose expression was sustained during continuous treatment and declined upon ABA withdrawal (M4). Notably, this ABA-activated module included genes encoding the core ABA signalling component (HAB) and regulatory feedback loops as well as genes involved in ABA metabolism, cell rescue, osmosensing, redox homeostasis, lipid droplet metabolism, water movement, and nitrate transport. These ABA responses functionally overlap with the transcriptional responses to water deficit in Arabidopsis and poplar [62,73,74,90,92], suggesting that evolutionary forces have conserved the coupling of ABA signal regulation and stress responses at the transcriptional level, even in the context of genome expansion.

In addition to ABA activation, ABA triggered widespread transcriptional inactivation. WGCNA identified a module of co-expressed genes whose expression was rapidly repressed and remained low as long as the ABA signal was active (M3). ABA inactivated a wide range of transporters, metabolic pathways, and transcription factors. To the best of our knowledge, the regulatory architecture underlying most of this ABA-mediated transcriptional repression has not yet been elucidated. Here, we showed that the ABA-inactivated module contained a suite of photosystem- and tetrapyrrole-related genes as well as the master regulators of leaf senescence, the GLK–BCM pathway (Fig 7). In Arabidopsis, GLK1 is essential for chloroplast maintenance, for the expression of photosystem- and tetrapyrrole-related genes, and for the activation of GUN4 and BCMs [135,136,160]. *GLK1* overexpression induces chloroplast development in Arabidopsis roots [168]. BCM promotes chlorophyll synthesis by interacting with GUN4 and inhibits chlorophyll degradation by destabilizing the Mg-dechelatase SGR1 [135]. Reduced GLK1 activity initiates leaf senescence by down-regulating *BCM* expression, thereby promoting chlorophyll breakdown [136,160,169]. During leaf development, GLK1 is post-transcriptionally repressed by RPGE2 and transcriptionally activated or repressed by CIA2/CIL and ATAF1, respectively [137,146,162]. ATAF1 binds the *NCED3* promoter, activates *NCED3* expression, and increases ABA biosynthesis, forming a feedback loop that represses *GLK1* expression [146,160,170]. In poplar, *PtATAF1.1*/*PtATAF1.2*, *PtNCED3*, and *PtNCED3-1* were activated by ABA while *GLK* homologs were repressed, suggesting that these regulatory loops are conserved. SNAC transcription factors also transcriptionally up-regulate SGR1, which promotes chlorophyll degradation during senescence [171,172]. PtRD26 directly targets several senescence-associated genes, including *PtSGR1* [142]. In Arabidopsis, *SGR1* expression is directly activated by ABFs and ANAC016 [1,173]. Together, these findings show that ABA-dependent transcriptional regulation extends to key molecular regulators of leaf senescence in poplar roots. *GLK* expression is also strongly down-regulated under drought in poplar roots [74]. While the ABA signal is likely to account for these transcriptional patterns, it also raises intriguing questions about the specificity of processes typically associated with leaf senescence, but occurring in roots.

ABA decreased the expression of genes in several metabolic pathways, including flavonoid and anthocyanin biosynthesis. In Arabidopsis seedlings, GLK1 is a positive regulator of anthocyanin accumulation and the loss of *glk1* function leads to reduced anthocyanin synthesis [174]. Consequently, ABA-dependent transcriptional repression of *GLK* homologs could inactivate the flavonoid/anthocyanin biosynthesis pathway in roots. Further functional studies are required to determine whether poplar GLKs directly regulate biosynthetic genes or act through MWB complex regulators, as previously proposed [174,175]. In poplar, ABA antagonised several regulators of the flavonoid/anthocyanin pathway, including well-known MYB repressors such as LTF15 and MYC2 homologs [40,119–124]. ABA-activated SNACs, *PtNAC049/PtNAC078*, may act as master repressors, consistent with the role of Arabidopsis ANAC032, which inhibits the anthocyanin/flavonoid pathway by down-regulating the expression of both biosynthetic and regulatory genes [176,177]. Furthermore, ABA selectively inactivated the MEP pathway and downstream hormone biosynthesis pathways, while promoting its own biosynthesis. These transcriptional patterns suggest a coordinated regulation of the isoprenoid pathway towards ABA synthesis, potentially reinforced by yet unexplored mechanisms diverting violaxanthin from the ketocarotenoid biosynthesis to ABA biosynthesis. In Arabidopsis leaves, GLKs act as transcriptional regulators that activate the transcription of GBF targets, including *deoxy-D-xylulose 5-phosphate synthase, phytoene synthase,* and *β-carotene hydroxylase 2*, as well as genes required for chloroplast function [178]. In poplar roots, ABA repressed *GLKs,* activated *GBFs*, and differentially regulated their putative targets. For instance, the multiple poplar *PSY* paralogs exhibited distinct ABA responses, suggesting that paralogy diversified trans-regulatory processes. However, these transcriptional patterns also support the long-standing hypothesis that transcriptional control orchestrates secondary metabolismin an evolutionary conserved and lineage-specific manner [178–180]. Carotenoid and chlorophyll biosynthesis are also under the post-transcriptional control of OR family proteins in chloroplasts [181], suggesting that both transcriptional and post-transcriptional regulation co-occurred for secondary metabolism.

ABA extensively rewired the root transcriptional landscape and remodelled several molecular processes (Fig 7). A key finding is that ABA signalling rapidly engaged coordinated regulatory pathways and functional responses previously associated with processes such as germination, stress responses, metabolic regulation, and leaf senescence, although these processes have not previously been reported to share common regulatory features. ABA-activated responses are likely to contribute to cell protection against damage, while ABA-repressed responses are mainly involved in transport and secondary metabolic pathways, whose importance in root remains to be investigated. Among these, the transcriptional inactivation of the tetrapyrrole and chlorophyll metabolism in roots is functionally intriguing. In Arabidopsis cells, ABA-driven inhibition of the chlorophyll branch in the tetrapyrrole pathway has been proposed to redirect protoporphyrin IX towards the heme biosynthesis [110]. ABA-driven increases in heme levels may contribute to cell protection against oxidative stress. An antioxidant molecule (biliverdin) can be produced from the degradation of heme in plastid [110]. Additionally, heme may be exported from plastids and incorporated into heme-binding proteins, including enzymes involved in ROS detoxification (catalase or peroxidase) but also other hemoproteins such as ABA-8’-hydroxylases (CYP707A) involved in ABA catabolism and homeostasis [182]. Based on the proposed working model, we hypothesize that cost-benefit trade-offs could have shaped ABA responses in roots, repressing the expression of accessory, yet costly, metabolic processes and directing gene expression changes towards adaptive responses.

Many other genes were identified as early ABA-responsive, yet they were not included in our working model due to limited knowledge of their functions. ABA is known to modulate transcriptional networks that control root development. In Arabidopsis, the SHR–SCR–SCL23 interaction specifies cell fate in the root meristem [183]. However, gene duplication diversified poplar SHR functions: PtSHR1 retains canonical interaction with PtSCR, whereas PtSHR2B influences phellogen activity, reduces tree growth and alters stem anatomy [184,185]. Meanwhile, *PtSHR2B* was expressed at the root tip [185]. Here, the co-repression of *PtSHR2B* and *PtGRAS39* in ABA-treated roots is particularly intriguing, and highlights an attractive target for future research. Overall, the comprehensive view of the early transcriptional landscape of ABA responses in poplar roots highlights primary targets of ABA, some lineage-specific innovations in the poplar genome, and core ABA-dependent responses encompassing both repression and activation.

## Materials and methods

Woody cuttings (*Populus nigra* clone 6J29) were surface sterilized with hydrogen peroxide [186], and individually set in hydroponic tanks containing 18L of modified Hoagland solution (NS) [187]. To prevent light exposure, the culture system was opaque and placed in a dark room (mean air temperature: 23.7°C ± 0.9°C). The solution was aerated to provide sufficient oxygen to the roots and buds were removed to prevent leaf appearance. This experimental set-up therefore controlled for well-known confounding effects in time-resolved transcriptomic studies of roots, namely light and its impact on shoot-to-root communication. After one week, NS was renewed and rooted cuttings were randomly assigned to treatments. For CTL, fresh NS was supplemented with 238 µL ethanol 96%. A 150 mM stock solution of ABA was prepared in ethanol 96% (Sigma-Aldrich) and used to prepare 2 µM ABA-enriched NS (ABA+). Treatments were applied by carefully transferring the cuttings either into ABA+ for 2, 5, 15, 30, 60, 180, 480, or 960 min or into CTL. For ABA withdrawal modality (ABA+/-), cuttings were transferred twice as they were pretreated with ABA+ for 180 min prior being transferred into CTL for 60, 180, 240, or 960 min. Harvests were arranged according to a full randomized design. Two independent experiments (Exp1 and Exp2 with two overlapping treatment time points, Fig 2A) were performed under the same growth conditions in July and September 2018, respectively.

### Hormone profiling

Hormone profiling was performed in Exp1 under ABA-free conditions (CTL) and under ABA+ for 5, 30, 60, or 180 min. 2-cm long root tips were collected, rinsed rapidly in distilled water, surface-dried, flash-frozen in liquid nitrogen and freeze-dried. Each of the four replicates consisted in 25 roots collected on 12 cuttings. Endogenous hormone contents and their metabolites were determined from 3 mg dry weight according to the method described by Šimura et al. [188] and previously used on poplar roots [189]. Analyte concentrations were calculated as ratios of non-labelled compounds to labelled internal standards or closely eluting stable isotope-labelled tracers using the standard isotope dilution method. The endogenous hormone contents were compared in ABA+-treated roots (for 5 min, 30 min, 60 min, and 180 min) versus CTL roots (*P*-value ≤ 0.05, Student’s t-test). The covariation of hormone contents across samples was determined using Pearson correlation coefficients (n = 20 samples, *P* ≤ 0.01).

### RNA isolation, cDNA library construction and RNA-Sequencing

In Exp1 and Exp2, 2-cm long root tips were collected and immediately fixed in RNAlater (Sigma-Aldrich). All samples were stored at -80°C and further processed together in a single batch for RNA extraction as well as for downstream RNA-seq library preparation and sequencing. The two to four replicates per time point were obtained by extracting RNA from a pool of eight root tips using a Spectrum Plant Total RNA kit (Sigma-Aldrich,) complemented by a DNase I treatment. RNA quality was checked using a Fragment Analyzer 5200 (Agilent Technologies). For details of cDNA library construction please refer to the S1A Protocol. The 48 cDNA libraries were normalized, multiplexed, and sequenced on an Illumina HiSeq3000 at the GeT-Plage core facility (http://www.get.genotoul.fr). RNA sequencing generated about 80 million paired-ends reads of 150 bp per library (S8 Data). RNA-seq data were pre-processed as described in Royer et al. [62] using *P. trichocarpa* genome v4.1 [166,190]. For details of RNA sequencing processing please refer to the S1B Protocol.

### Gene annotation and Functional enrichments

Gene annotation was retrieved from *P. trichocarpa* V4.1 annotation files (S3 Data). Functional categorization was achieved using Gene Ontology (GO) and MapMan resources [191,192]. For details, please refer to the S1C Protocol. Significantly enriched GO terms and MapMan bins, as well as iDREM path assignation, were examined through a Fisher’s exact test combined with an FDR correction for multiple testing (corrected *P*-value threshold of 0.05).

### Expression analysis

Normalized read counts, differential expression and significance statistics were computed with Deseq2 v1.24.0 [193]. Normalized count table was reduced to loci showing a robust expression for at least one treatment (i.e., no less than 10 aligned reads in all the libraries documenting one treatment). Hierarchical clustering on Log2 (normalized count+1) revealed two outlier libraries which were not further considered (S8 Data). No batch correction was applied prior to analysis. The expression matrix consisted in 27,379 loci over 46 libraries.

The pattern of temporal expression was revealed using iDREM [56,194]. This software uses an input–output hidden Markov model to reconstruct regulatory networks while taking into account their dynamics [195]. iDREM model was inferred in the early part of the time series (Exp1: CTL and ABA+ for 2, 5, 15, 30, and 60 min), using default parameter, selecting Spot ID and allowing transformation of the time series to start at zero for CTL and Log-transformation of expression. iDREM was run using mean expression value per time points for the 1,914 loci differentially expressed in response to ABA at least at one of time point in comparison with CTL (absolute Log2-transformed fold-change expression ≥ 1 and a *P*-value ≤ 0.01, Wald test *P*-values corrected for multiple testing by applying FDR correction). iDREM filtered loci if the maximum absolute difference between transformed expression values of any two time points was less than one. The reconstructed temporal map described the groups of co-expressed loci as paths and highlighted the nodes where paths split. Additionally, we constructed an iDREM-derived gene regulatory network (GRN) for the putative transcription factors predicted to regulate transition events on the iDREM temporal map (split cut-off score < 0.0001). This GRN was inferred from the prediction of transcription factor-gene interaction, the temporal expression patterns of target genes, and the path transition events regulated by each transcription factor (S1 Fig and S2 Data). iDREM scored transcription factors with splits and paths based on both gene assignments and prediction about transcription factor-gene interaction in *P. trichocarpa* V3.0 genome (released on 2019-07, plantregmap.gao-lab.org).

### Co-expression networks

Co-expression network analysis was performed using the R package WGCNA v1.70 [59,196]. The expression matrix (27,379 loci x 46 libraries, further referred to as the reference set) was Log2 (normalized count+1)-transformed. Scale-free topology was obtained by applying a soft power of 18 (S13 Fig). The network was constructed using the “blockwiseModules” function (parameter: signed network type, bicor, power = 18, maxBlockSize = 15,000, minModuleSize = 150, reassignThreshold = 1e^-6^, mergeCutHeight = 0.25, maxPOutliers = 0.05, and deepSplit = 3). WGCNA identifies modules of highly correlated loci and provides module eigengene (ME), which is the first principal component of each module. Module-based biological significance was computed as the value of the correlation between the mean ME values and the mean hormone levels over five time points in Exp1 (CTL and ABA+ for 5, 30, 60, and 180 min). Intramodular connectivity (Kin), which is the sum of a locus’ connection strengths with all other loci in the module, was computed using “intramodularConnectivity.fromExpr” function and scaled Kin by calling scaleByMax option. We selected as hubs any loci that exhibited a scaled Kin ≥ 0.75. The topological overlap (TO), which is the similarities of two loci relationships with all other loci in the network [60], was computed using “vectorTOM” function and hubs as input against the whole expression matrix.

Hub-hub correlation network was built using the hub expression matrix as input for R package MEGENA v1.4.1 [197]. Pearson correlation matrix was summarized into MEGENA-derived Planar Filtered Network, using default parameters. Networks were visualized using the R package igraph, v1.2.4.1 [198].

### Module preservation in osmotically stressed samples

We re-analysed 32 libraries documenting CTL and osmotically-stressed root tips (−0.35 MPa, for 30 min or 180 min) in the division zone and the elongation zone of *P. nigra* roots (SRA: SRP067564 [62]). Taking advantage from paired-sampling procedure, the root transcriptome was built by concatenating the FastQ files from the two root zones. The resulting 16 libraries were processed as described above. Normalized and Log2 (normalized count+1)-transformed expression matrices from reference and test sets were embedded into a multi-experiment dataset (24,962 loci x 46 and 16 libraries).

WGCNA assesses the module robustness and replicability across datasets [61]. Preservation statistics were computed using “modulePreservation” function (networkType = “signed,” corFnc = “bicor,” maxModuleSize = 5000, nPermutations = 200). Zsummary, a composite statistic based on density and connectivity metrics, and Zmean.Adj, a module density-based metrics were extracted. A high Zsummary (>10) reveals a strong module structure preservation among the two sets while Zmean.Adj denotes how much module-embedded loci remain connected in the test set.

Modular structure preservation was assessed using DGCA, a bottom-up approach based on differential gene correlation analysis [63]. DGCA quantifies the modular average differential connectivity, MeDC, as the mean change in Z-score difference among all module-embedded locus pairs between reference and test sets, and assesses its statistical significance using empirical *P*-values (moduleDC DGCA function, nperm = 75). Negative MeDC values indicated a loss of connectivity for the module in the test set with respect to the reference. Differential pairwise relationships were computed using the DGCA functions “getCors”, “pairwiseDCor”, and “dcTopPairs” with default parameters (except for: adjust = “fdr,” corSigThresh = 0.001).

### Statistical analyses

Statistical analyses and plots were performed using R 3.6.1 (R Development Core Team, http://www.r-project.org/).

### Data statement

Reads were deposited in the Sequence Read Archive (SRA) under the Study Accession number GSE183780. The expression data are provided in S3 Data. Identifier of genes mentioned in the main text are given in S2 Appendix.

## Supporting information

S1 Fig ABA-dependent gene regulatory network predicted in poplar roots (iDREM-derived GRN). The first layer consists of transcription factors identified by the iDREM regulatory map as the most likely regulators of path transition events. The second layer consists of their target genes within each iDREM path. Node colors denote (A) iDREM paths, (B) WGCNA modules, (C) differentially expressed genes under osmotic stress in comparison with CTL (absolute Log2-transformed fold-change expression ≥ 1 and a *P*-value ≤ 0.01, Wald test P-values corrected for multiple testing by applying FDR correction).
(PDF)

S2 Fig Genes encoding putative post-transcriptional regulators acting on the core ABA signalling components. (PDF)

S3 Fig M4 genes encoding proteins putatively involved in (A) cell protection, (B) raffinose family oligosaccharides metabolism, (C) M4 genes encoding putative LATE EMBRYOGENESIS ABUNDANT (LEA) proteins, (D) Genes encoding putative lipid droplet associated proteins, (E) Further genes contributing to M4 enrichment in “Bin 3: minor CHO metabolism”. (PDF)

S4 Fig Genes encoding proteins involved in (A) the methylerythritol phosphate (MEP) pathway, in (B) the branch points of the MEP pathway, in (C) carotenoid metabolism, in (D) the conversion of β-carotene to zeaxanthin and ABA metabolism, and in (E) lutein and capsanthin synthesis. The $ sign indicates that the AAO3 function linked to ABA has been questioned in poplar [42].
(PDF)

S5 Fig Genes encoding proteins involved in (A) strigolactone synthesis and in (B) gibberellin metabolism. (PDF)

S6 Fig Genes encoding proteins involved in (A) cytokinin metabolism and (B) cytokinin signalling. (PDF)

S7 Fig Genes encoding putative transporters and channels. (A) Genes encoding putative nitrate transporters. The $ sign indicates putative ABA transporter of the *PtNPF* family. (B) Genes encoding other putative ABA transporters of the AWPM-19, ABCG and DTX families. (C) *Aquaporin* and (D) *OSCA* family. (E) Genes related to anthocyanin/flavonoid transport. (F) Further M3 and M4 hub genes encoding miscellaneous transporters.
(PDF)

S8 Fig Genes encoding proteins putatively involved in (A) nitrogen metabolism and in (B) oxidative pentose phosphate (OPP) pathway. (PDF)

S9 Fig Genes encoding proteins putatively involved in (A-B) flavonoid metabolism, (B) Further genes contributing to M3 enrichment in “Bin16.8: secondary metabolism.flavonoids”, (C-F) Genes related to the regulation of anthocyanin/proanthocyanidin synthesis. R2R3-MYB transcription factors acting as (C) activators and (D) repressors. The $ sign indicates R2R3-MYB Subfamily S4 (Du et al. 2015). Genes encoding other putative members of the regulatory MBW complex belonging to (E) WD40 repeat family protein and (F) bHLH transcription factor family. (PDF)

S10 Fig Genes contributing to M3 enrichments in (A) “Bin1: photosystem” and (B) “Bin19: tetrapyrrole synthesis”, (C) Poplar homologs and orthologs of key regulatory genes acting on chlorophyll homeostasis in Arabidopsis and SAG201 homologs. (PDF)

S11 Fig Genes encoding further transcription factors (A) predicted by iDREM or putatively involved in ABA signal amplification and diversification, including (B) Stress-responsive NAC (SNAC) and other NAC transcription factors, (C) R2R3-MYBs and (D) transcription factors from miscellaneous families. (PDF)

S12 Fig M4 and M3 hub genes not currently associated with a specific pathway or process, or encoding proteins of unknown function. (A) Hub genes encoding miscellaneous enzymes and/or previously named. (B) Further hub genes with miscellaneous motif and domains.
(PDF)

S13 Fig Co-expression network construction. (PDF)

S1 Data Hormone contents. (XLSX)

S2 Data iDREM analysis, including iDREM-derived GRN. (XLSX)

S3 Data Loci annotation, expression and differential expression and WGCNA information (module assignation, loci connectivity and ME x sample matrix). (XLSX)

S4 Data Gene Ontology enrichment for co-expressed unigenes per module. (XLSX)

S5 Data MapMan Bin enrichment for co-expressed unigenes per module. (XLSX)

S6 Data Hub network. (XLSX)

S7 Data DGCA for reference Hubs. (XLSX)

S8 Data RNA-Seq pre-processing. (XLSX)

S1 Protocol (A) cDNA library construction, (B) RNA sequencing processing, and (C) Gene Ontology and Mapman bin. (DOCX)

S1 Appendix Additional information about transporters and channels. (DOCX)

S2 Appendix Genes identifiers mentioned in the main text and Appendix. (DOCX)
