# Supplementary material for: Gene network analysis predicts the primary regulators of ABA-dependent transcriptional activation and repression in Populus roots
Source: PLoS Genet. 2026 Aug 4;22(8):e1012256. doi: 10.1371/journal.pgen.1012256 (PMC13466054; doi:10.1371/journal.pgen.1012256)
Supplement: Supplementary file 1 [file pgen.1012256.s001.pdf]

Cohen *et al.* Gene network analysis predicts the primary regulators of ABA-dependent transcriptional activation and repression in *Populus* roots

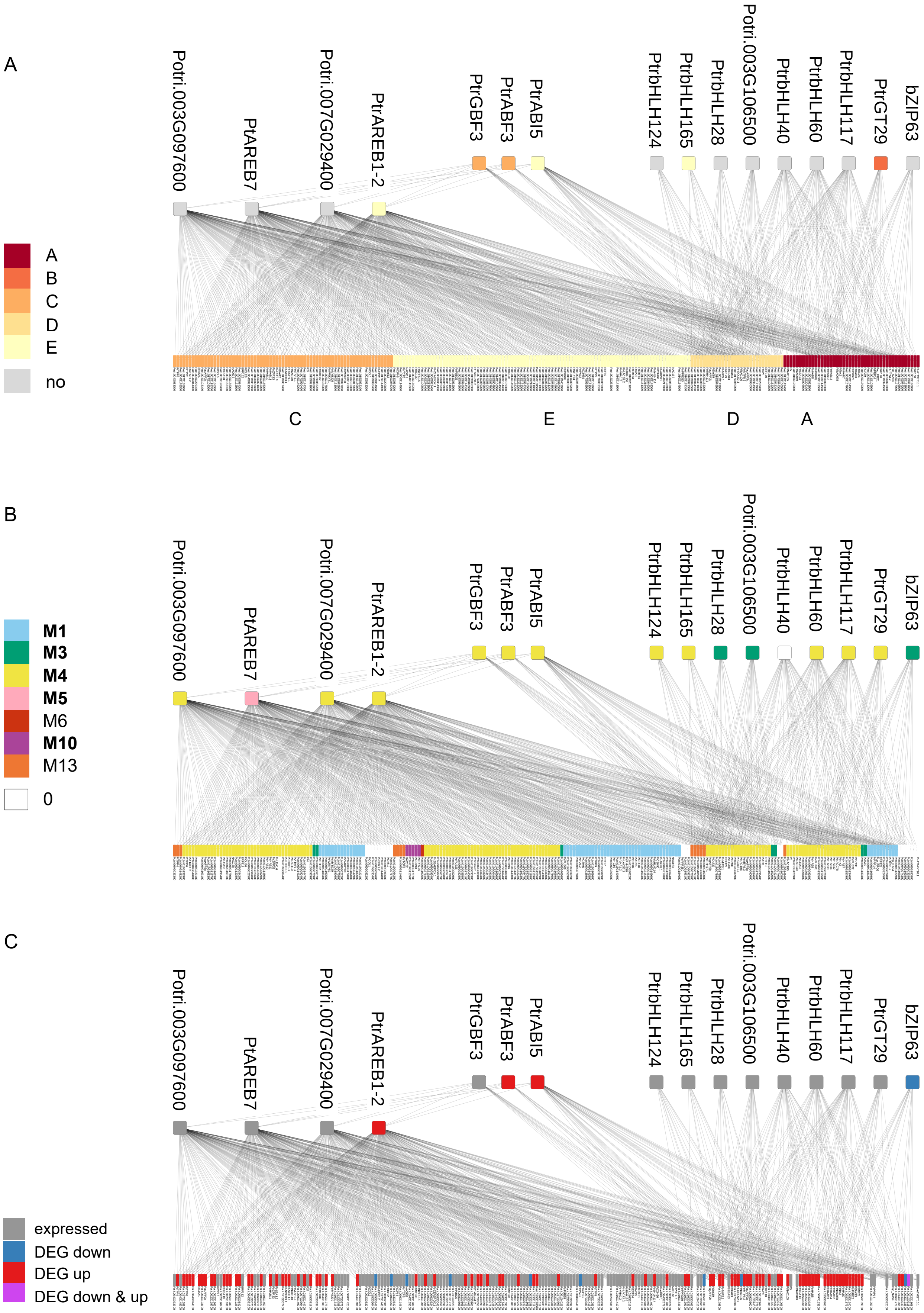

S1 Figure. ABA-dependent gene regulatory network predicted in poplar roots (iDREM-derived GRN). The first layer consists of transcription factors identified by the iDREM regulatory map as the most likely regulators of path transition events. The second layer consists of their target genes within each iDREM path. Node colors denote (A) iDREM paths, (B) WGCNA modules, (C) differentially expressed genes under osmotic stress in comparison with CTL (absolute Log2-transformed fold-change expression ≥ 1 and a *P*-value ≤ 0.01, Wald test *P*-values corrected for multiple testing by applying FDR correction)
