# Supplementary material for: Gene network analysis predicts the primary regulators of ABA-dependent transcriptional activation and repression in Populus roots
Source: PLoS Genet. 2026 Aug 4;22(8):e1012256. doi: 10.1371/journal.pgen.1012256 (PMC13466054; doi:10.1371/journal.pgen.1012256)
Supplement: Supplementary file 2 [file pgen.1012256.s002.pdf]

Cohen *et al*. Gene network analysis predicts the primary regulators of ABA-dependent transcriptional activation and repression in *Populus* roots

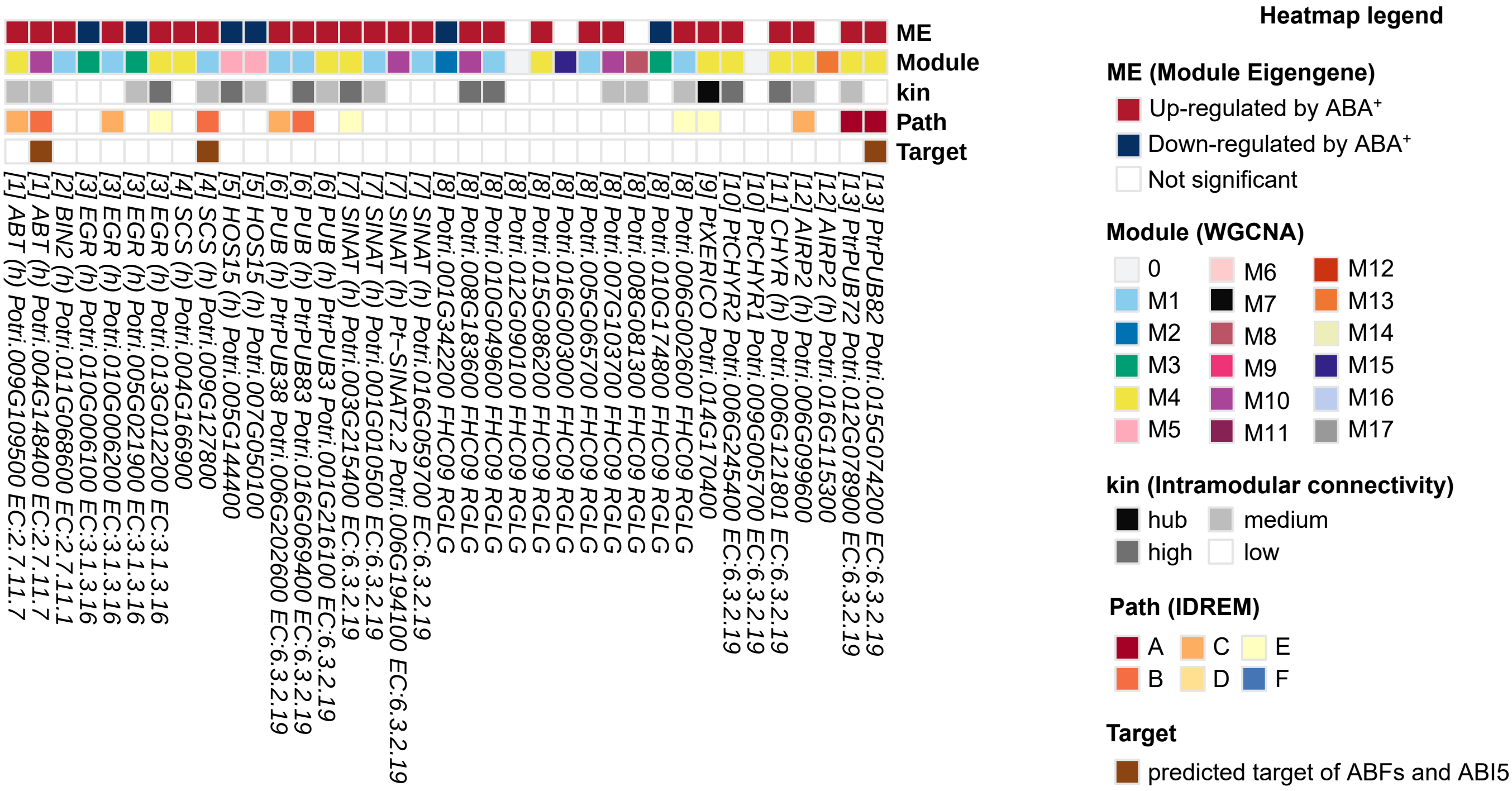

S2 Figure: Genes encoding putative post-transcriptional regulators acting on the core ABA signalling components.

The heatmap summarizes the main transcriptional features of WGCNA analysis, namely gene assignation to ABA-activated or ABA-inactivated modules (ME, Fig 3B), module assignation (Module) and scaled intramodular connectivity (kin), of iDREM analysis (Path), and of *in silico* prediction about ABI5 and/or ABFs gene interaction from plantregmap.gao-lab.org (Target). [number] indicates literature source listed below; (h) indicates homology-based information by querying comparative genomics databases. Only unigenes expressed in roots are shown, identifiers refer to *P. trichocarpa* V4.1 genome.

References

1. Wang Z, Ren Z, Cheng C, Wang T, Ji H, Zhao Y, Deng Z, Zhi L, Lu J, Wu X, et al. Counteraction of ABA Mediated Inhibition of Seed Germination and Seedling Establishment by ABA Signaling Terminator in Arabidopsis. Molecular Plant. 2020b:13(9):1284–1297. https://doi.org/10.1016/j.molp.2020.06.011

2. Cai Z, Liu J, Wang H, Yang C, Chen Y, Li Y, Pan S, Dong R, Tang G, Barajas-Lopez JDD, et al. GSK3-like kinases positively modulate abscisic acid signaling through phosphorylating subgroup III SnRK2s in Arabidopsis. Proc Natl Acad Sci USA. 2014:111(26):9651–9656. https://doi.org/10.1073/pnas.1316717111

3. Li C, Li X, Deng Z, Song Y, Liu X, Tang XA, Li Z, Zhang Y, Zhang B, Tang W, et al. EGR1 and EGR2 positively regulate plant ABA signaling by modulating the phosphorylation of SNRK2 .2. New Phytologist. 2024:241(4):1492–1509. https://doi.org/10.1111/nph.19458

4. Tarnowski K, Klimecka M, Ciesielski A, Goch G, Kulik A, Fedak H, Poznański J, Lichocka M, Pierechod M, Engh RA, et al. Two SnRK2-Interacting Calcium Sensor Isoforms Negatively Regulate SnRK2 Activity by Different Mechanisms. Plant Physiol. 2020:182(2):1142–1160. https://doi.org/10.1104/pp.19.00900

5. Ali A, Kim JK, Jan M, Khan HA, Khan IU, Shen M, Park J, Lim CJ, Hussain S, Baek D, et al. Rheostatic Control of ABA Signaling through HOS15-Mediated OST1 Degradation. Molecular Plant. 2019:12(11):1447–1462. https://doi.org/10.1016/j.molp.2019.08.005

6. Zhao J, Zhao L, Zhang M, Zafar S, Fang J, Li M, Zhang W, and Li X. Arabidopsis E3 Ubiquitin Ligases PUB22 and PUB23 Negatively Regulate Drought Tolerance by Targeting ABA Receptor PYL9 for Degradation. IJMS. 2017:18(9):1841. https://doi.org/10.3390/ijms18091841

7. Xia F-N, Zeng B, Liu H-S, Qi H, Xie L-J, Yu L-J, Chen Q-F, Li J-F, Chen Y-Q, Jiang L, et al. SINAT E3 Ubiquitin Ligases Mediate FREE1 and VPS23A Degradation to Modulate Abscisic Acid Signaling. Plant Cell. 2020:32(10):3290–3310. https://doi.org/10.1105/tpc.20.00267

8. Jiménez-López D, Muñóz-Belman F, González-Prieto JM, Aguilar-Hernández V, and Guzmán P. Repertoire of plant RING E3 ubiquitin ligases revisited: New groups counting gene families and single genes. PLoS ONE. 2018:13(8):e0203442. https://doi.org/10.1371/journal.pone.0203442

9. Kim M-H, Cho J-S, Park E-J, Lee H, Choi Y-I, Bae E-K, Han K-H, and Ko J-H. Overexpression of a Poplar RING-H2 Zinc Finger, Ptxerico, Confers Enhanced Drought Tolerance via Reduced Water Loss and Ion Leakage in Populus. IJMS. 2020:21(24):9454. https://doi.org/10.3390/ijms21249454

10. He F, Wang H-L, Li H-G, Su Y, Li S, Yang Y, Feng C-H, Yin W, and Xia X. PeCHYR1, a ubiquitin E3 ligase from Populus euphratica , enhances drought tolerance via ABA-induced stomatal closure by ROS production in Populus. Plant Biotechnol J. 2018:16(8):1514–1528. https://doi.org/10.1111/pbi.12893

11. Ding S, Zhang B, and Qin F. Arabidopsis RZFP34/CHYR1, a Ubiquitin E3 Ligase, Regulates Stomatal Movement and Drought Tolerance via SnRK2.6-Mediated Phosphorylation. Plant Cell. 2015:27(11):3228–3244. https://doi.org/10.1105/tpc.15.00321

12. Cho SK, Ryu MY, Seo DH, Kang BG, and Kim WT. The Arabidopsis RING E3 Ubiquitin Ligase AtAIRP2 Plays Combinatory Roles with AtAIRP1 in Abscisic Acid-Mediated Drought Stress Responses. Plant Physiology. 2011:157(4):2240–2257. https://doi.org/10.1104/pp.111.185595

13. Tong S, Chen N, Wang D, Ai F, Liu B, Ren L, Chen Y, Zhang J, Lou S, Liu H, et al. The U-box E3 ubiquitin ligase PalPUB79 positively regulates ABA-dependent drought tolerance via ubiquitination of PalWRKY77 in Populus. Plant Biotechnology Journal. 2021:19(12):2561–2575. https://doi.org/10.1111/pbi.13681

Arabidopsis identifiers :

ABT: AIW1 (AT3G18950), AIW2 (AT1G49450); AIRP2 (AT5G01520); BIN2 (AT4G18710); CHYR: CHYR1 (AT5G22920), CHYR2 (AT5G25560); EGR: EGR1 (AT3G05640), EGR2 (AT5G27930), EGR3 (AT3G16800); HOS15 (AT5G67320); PUB: ATPUB22 (AT3G52450), ATPUB23 (AT2G35930); SINAT: SINAT1 (AT2G41980), SINAT2 (AT3G58040)
