# Supplementary material for: Gene network analysis predicts the primary regulators of ABA-dependent transcriptional activation and repression in Populus roots
Source: PLoS Genet. 2026 Aug 4;22(8):e1012256. doi: 10.1371/journal.pgen.1012256 (PMC13466054; doi:10.1371/journal.pgen.1012256)
Supplement: Supplementary file 3 [file pgen.1012256.s003.pdf]

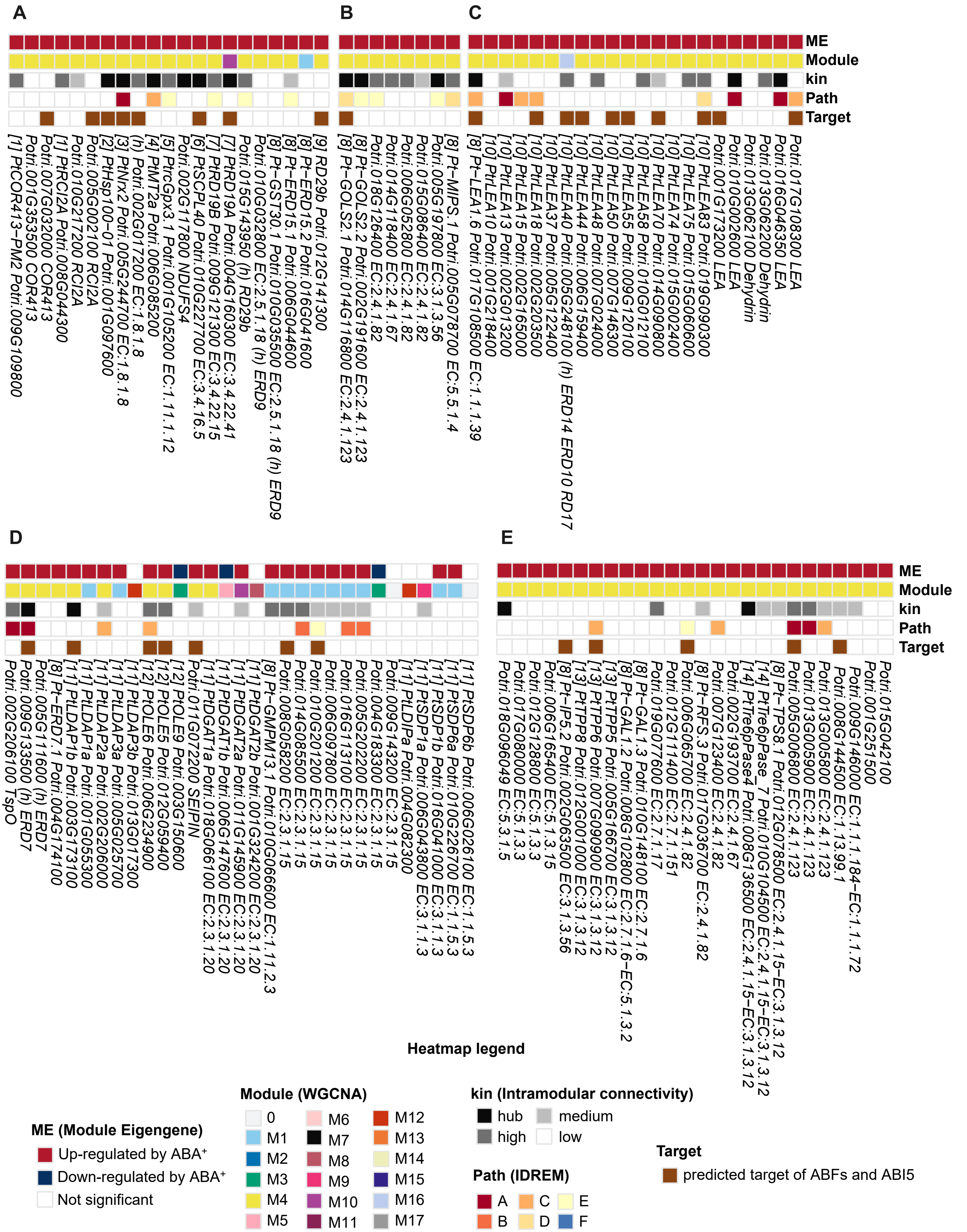

S3 Figure: M4 genes encoding proteins putatively involved in (A) cell protection and (B) raffinose family oligosaccharides metabolism. (C) M4 genes encoding putative LATE EMBRYOGENESIS ABUNDANT (LEA) proteins. (D) Genes encoding putative lipid droplet associated proteins. (E) Further genes contributing to M4 enrichment in “Bin 3: minor CHO metabolism”.

The heatmaps summarize the main transcriptional features of WGCNA analysis, namely gene assignation to ABA-activated or ABA-inactivated modules (ME, Fig 3B), module assignation (Module) and scaled intramodular connectivity (kin), of iDREM analysis (Path), and of *in silico* prediction about ABI5 and/or ABFs gene interaction from plantregmap.gao-lab.org (Target).[number] indicates literature source listed below; (h) indicates homology-based information by querying comparative genomics databases. Only unigenes expressed in roots are shown, identifiers refer to *P. trichocarpa* V4.1 genome.

References

1. Tian H, Chen S, Yang W, Wang T, Zheng K, Wang Y, Cheng Y, Zhang N, Liu S, Li D, et al. A novel family of transcription factors conserved in angiosperms is required for ABA signalling. Plant Cell Environ. 2017:40(12):2958–2971. htps://doi.org/10.1111/pce.13058

2. Yer EN, Baloglu MC, and Ayan S. Identification and expression profiling of all Hsp family member genes under salinity stress in different poplar clones. Gene. 2018:678:324–336. htps://doi.org/10.1016/j.gene.2018.08.049

3. Chibani K, Wingsle G, Jacquot J-P, Gelhaye E, and Rouhier N. Comparative Genomic Study of the Thioredoxin Family in Photosynthetic Organisms with Emphasis on Populus trichocarpa. Molecular Plant. 2009:2(2):308–322. htps://doi.org/10.1093/mp/ssn076

4. De Oliveira VH, Ullah I, Dunwell JM, and Tibbet M. Mycorrhizal symbiosis induces divergent paterns of transport and partitioning of Cd and Zn in Populus trichocarpa. Environmental and Experimental Botany. 2020:171:103925. htps://doi.org/10.1016/j.envexpbot.2019.103925

5. Navrot N, Collin V, Gualberto J, Gelhaye E, Hirasawa M, Rey P, Knaff DB, Issakidis E, Jacquot J-P, and Rouhier N. Plant glutathione peroxidases are functional peroxiredoxins distributed in several subcellular compartments and regulated during biotic and abiotic stresses. Plant Physiol. 2006:142(4):1364–1379. htps://doi.org/10.1104/pp.106.089458

6. Zhu D, C hu W, W ang Y, Yan H , C hen Z , a nd X iang Y . Genome-wide identification, classification and expression analysis of the serine carboxypeptidase-like protein family in poplar. Physiol Plantarum. 2018:162(3):333–352. htps://doi.org/10.1111/ppl.12642

7. Zou Z, Xie G, and Yang L. Papain-like cysteine protease encoding genes in rubber (Hevea brasiliensis): comparative genomics, phylogenetic, and transcriptional profiling analysis. Planta. 2017:246(5):999–1018. htps://doi.org/10.1007/s00425-017-2739-z

8. Sjödin A, Street NR, Sandberg G, Gustafsson P, and Jansson S. The Populus Genome Integrative Explorer (PopGenIE): a new resource for exploring the Populus genome. New Phytologist. 2009:182(4):1013–1025. htps://doi.org/10.1111/j.1469-8137.2009.02807.x

9. Yang Y, Li H-G, Wang J, Wang H-L, He F, Su Y, Zhang Y, Feng C-H, Niu M, Li Z, et al. ABF3 enhances drought tolerance via promoting ABA-induced stomatal closure by directly regulating ADF5 in Populus euphratica. Journal of Experimental Botany. 2020:71(22):7270–7285. htps://doi.org/10.1093/jxb/eraa383

10. Cheng Z, Zhang X, Yao W, Zhao K, Liu L, Fan G, Zhou B, and Jiang T. Genome-wide search and structural and functional analyses for late embryogenesis-abundant (LEA) gene family in poplar. BMC Plant Biol. 2021:21(1):110. htps://doi.org/10.1186/s12870-021-02872-3

11. Veerabagu M, Rinne PLH, Skaugen M, Paul LK, and Van Der Schoot C. Lipid Body Dynamics in Shoot Meristems: Production, Enlargement, and Putative Organellar Interactions and Plasmodesmal Targeting. Front Plant Sci. 2021:12:674031. htps://doi.org/10.3389/fpls.2021.674031

12. Veerabagu M, K Paul L, Rinne PL, and Van Der Schoot C. Plant Lipid Bodies Traffic on Actin to Plasmodesmata Motorized by Myosin XIs. IJMS. 2020:21(4):1422. htps://doi.org/10.3390/ijms21041422

13. Gao Y, Yang X, Yang X, Zhao T, An X, and Chen Z. Characterization and expression patern of the trehalose-6-phosphate synthase and trehalose-6-phosphate phosphatase gene families in Populus. International Journal of Biological Macromolecules. 2021:187:9–23. htps://doi.org/10.1016/j.ijbiomac.2021.07.096

14. Geisler-Lee J, Geisler M, Coutinho PM, Segerman B, Nishikubo N, Takahashi J, Aspeborg H, Djerbi S, Master E, Andersson-Gunnerås S, et al. Poplar Carbohydrate-Active Enzymes. Gene Identification and Expression Analyses. Plant Physiology. 2006:140(3):946–962. htps://doi.org/10.1104/pp.105.072652

Arabidopsis identifiers :

ERD7 (AT2G17840); ERD9 (AT1G10370); ERD10 (AT1G20450); ERD14 (AT1G76180); NDUFS4 (AT5G67590); RD17 (AT1G20440); RD29b (AT5G52300)
