# Supplementary material for: Gene network analysis predicts the primary regulators of ABA-dependent transcriptional activation and repression in Populus roots
Source: PLoS Genet. 2026 Aug 4;22(8):e1012256. doi: 10.1371/journal.pgen.1012256 (PMC13466054; doi:10.1371/journal.pgen.1012256)
Supplement: Supplementary file 4 [file pgen.1012256.s004.pdf]

Cohen *et al*. Gene network analysis predicts the primary regulators of ABA-dependent transcriptional activation and repression in *Populus* roots

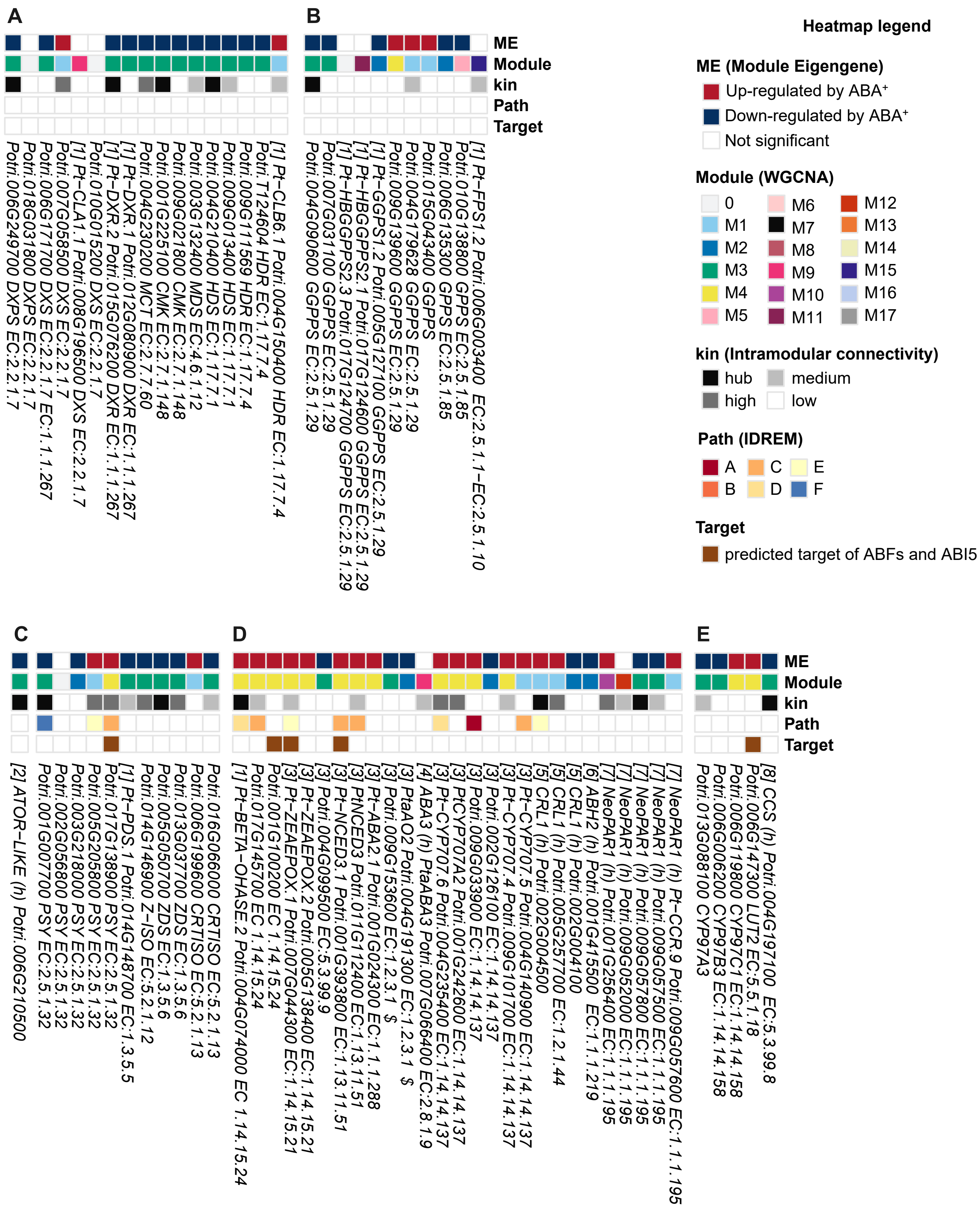

S4 Figure: Genes encoding proteins involved in (A) the methylerythritol phosphate (MEP) pathway, in (B) the branch points of the MEP pathway, in (C) carotenoid metabolism, in (D) the conversion of β-carotene to zeaxanthin and ABA metabolism, and in (E) lutein and capsanthin synthesis. The $ sign indicates that the AAO3 function linked to ABA has been questioned in poplar (Hanada et al. 2011).

The heatmaps summarize the main transcriptional features of WGCNA analysis, namely gene assignation to ABA-activated or ABA-inactivated modules (ME, Fig 3B), module assignation (Module) and scaled intramodular connectivity (kin), of iDREM analysis (Path), and of in silico prediction about ABI5 and/or ABFs gene interaction from plantregmap.gao-lab.org (Target). [number] indicates literature source listed below; (h) indicates homology-based information by querying comparative genomics databases. Only unigenes expressed in roots are shown, identifiers refer to *P. trichocarpa* V4.1 genome.

References

1. Sjödin A, Street NR, Sandberg G, Gustafsson P, and Jansson S. The Populus Genome Integrative Explorer (PopGenIE): a new resource for exploring the Populus genome. New Phytologist. 2009:182(4):1013–1025. htps://doi.org/10.1111/j.1469-8137.2009.02807.x

2. Zhou X, Welsch R, Yang Y, Álvarez D, Riediger M, Yuan H, Fish T, Liu J, Thannhauser TW, and Li L. Arabidopsis OR proteins are the major postranscriptional regulators of phytoene synthase in controlling carotenoid biosynthesis. Proc Natl Acad Sci USA. 2015:112(11):3558–3563. htps://doi.org/10.1073/pnas.1420831112

3. Hanada K, Hase T, Toyoda T, Shinozaki K, and Okamoto M. Origin and evolution of genes related to ABA metabolism and its signaling pathways. J Plant Res. 2011:124(4):455–465. htps://doi.org/10.1007/s10265-011-0431-0

4. Seo M, Peeters AJM, Koiwai H, Oritani T, Marion-Poll A, Zeevaart JAD, Koornneef M, Kamiya Y, and Koshiba T. The Arabidopsis aldehyde oxidase 3 (AAO3) gene product catalyzes the final step in abscisic acid biosynthesis in leaves. Proc Natl Acad Sci USA. 2000:97(23):12908–12913. htps://doi.org/10.1073/pnas.220426197

5. Yin X, Bai Y-L, Ye T, Yu M, Wu Y, and Feng Y-Q. Cinnamoyl coA: NADP oxidoreductase-like 1 regulates abscisic acid response by modulating phaseic acid homeostasis in Arabidopsis thaliana. Journal of Experimental Botany. 2022:73(3):860–872. tps://doi.org/10.1093/jxb/erab474

6. Weng J-K, Ye M, Li B, and Noel JP. Co-evolution of Hormone Metabolism and Signaling Networks Expands Plant Adaptive Plasticity. Cell. 2016:166(4):881–893. htps://doi.org/10.1016/j.cell.2016.06.027

7. Bai Y-L, Yin X, Xiong C-F, Cai B-D, Wu Y, Zhang X-Y, Wei Z, Ye T, and Feng Y-Q. Neophaseic acid catabolism in the 9’-hydroxylation pathway of abscisic acid in Arabidopsis thaliana. Plant Communications. 2022:3(5):100340. htps://doi.org/10.1016/j.xplc.2022.100340

8. Bouvier F, Hugueney P, d’Harlingue A, Kuntz M, and Camara B. Xanthophyll biosynthesis in chromoplasts: isolation and molecular cloning of an enzyme catalyzing the conversion of 5,6-epoxycarotenoid into ketocarotenoid. Plant J. 1994:6(1):45–54. htps://doi.org/10.1046/j.1365- 313X.1994.6010045.x

Arabidopsis identifiers :

ABA3 (AT1G16540); ABH2 (AT4G27250); AAO3 (AT2G27150); ATOR-LIKE (AT5G06130); CRL1 (AT2G33590); NeoPAR1 (AT1G09510)
