# Supplementary material for: Gene network analysis predicts the primary regulators of ABA-dependent transcriptional activation and repression in Populus roots
Source: PLoS Genet. 2026 Aug 4;22(8):e1012256. doi: 10.1371/journal.pgen.1012256 (PMC13466054; doi:10.1371/journal.pgen.1012256)
Supplement: Supplementary file 5 [file pgen.1012256.s005.pdf]

Cohen *et al*. Gene network analysis predicts the primary regulators of ABA-dependent transcriptional activation and repression in *Populus* roots

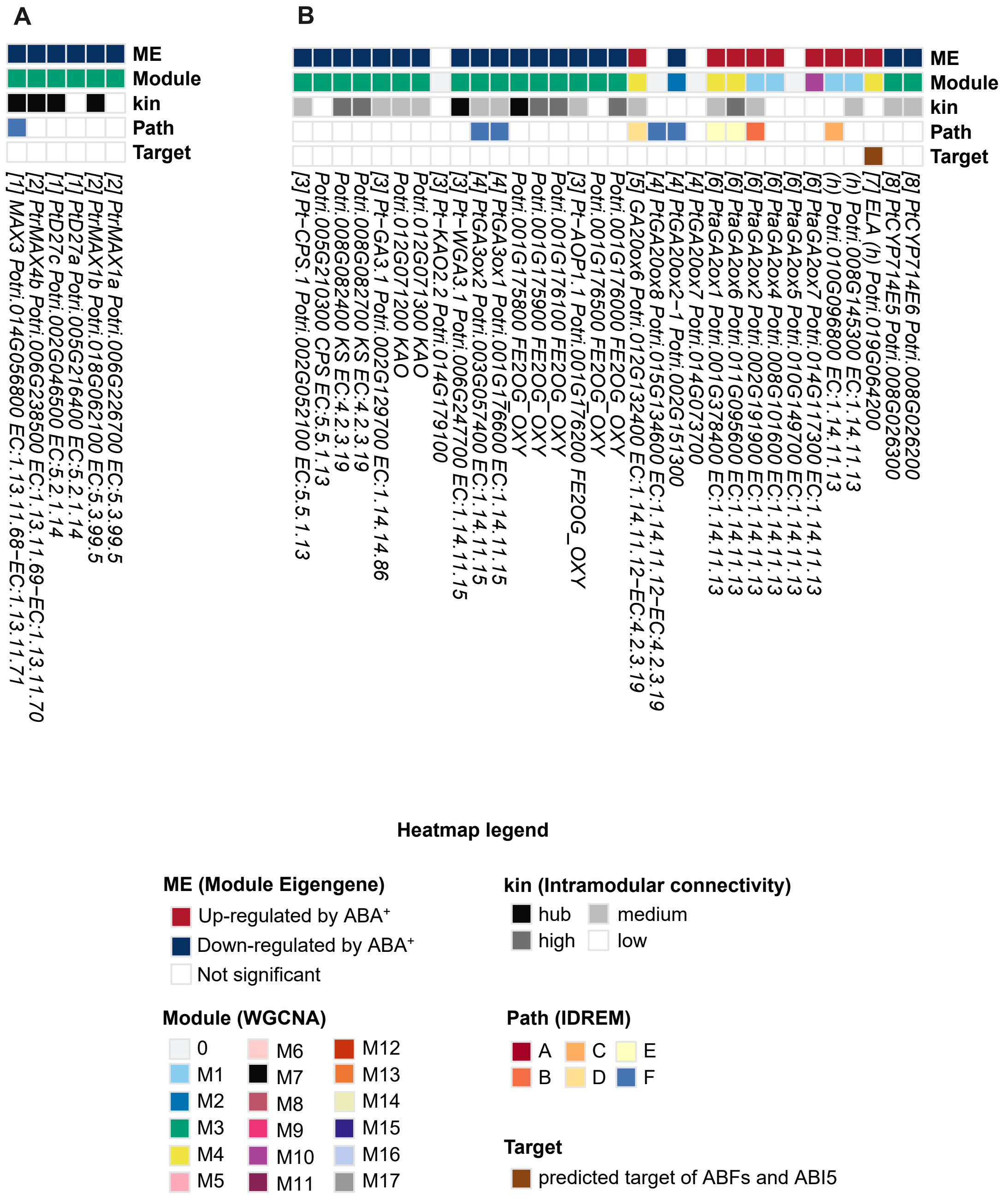

S5 Figure: Genes encoding proteins involved in (A) strigolactone synthesis and in (B) gibberellin metabolism.

The heatmaps summarize the main transcriptional features of WGCNA analysis, namely gene assignation to ABA-activated or ABA-inactivated modules (ME, Fig 3B), module assignation (Module) and scaled intramodular connectivity (kin), of iDREM analysis (Path), and of *in silico prediction* about ABI5 and/or ABFs gene interaction from plantregmap.gao-lab.org (Target). [number] indicates literature source listed below; (h) indicates homology-based information by querying comparative genomics databases. Only unigenes expressed in roots are shown, identifiers refer to *P. trichocarpa* V4.1 genome.

References

1. Katyayini NU, Rinne PLH, and Van Der Schoot C. Strigolactone-Based Node-to-Bud Signaling May Restrain Shoot Branching in Hybrid Aspen. Plant and Cell Physiology. 2019:60(12):2797–2811. htps://doi.org/10.1093/pcp/pcz170

2. Czarnecki O, Yang J, Wang X, Wang S, Muchero W, Tuskan GA, and Chen J-G. Characterization of MORE AXILLARY GROWTH Genes in Populus. PLoS ONE. 2014:9(7):e102757. htps://doi.org/10.1371/journal.pone.0102757

3. Sjödin A, Street NR, Sandberg G, Gustafsson P, and Jansson S. The Populus Genome Integrative Explorer (PopGenIE): a new resource for exploring the Populus genome. New Phytologist. 2009:182(4):1013–1025. htps://doi.org/10.1111/j.1469-8137.2009.02807.x

4. Katyayini NU, Rinne PLH, Tarkowská D, Strnad M, and Van Der Schoot C. Dual Role of Gibberellin in Perennial Shoot Branching: Inhibition and Activation. Front Plant Sci. 2020:11:736. htps://doi.org/10.3389/fpls.2020.00736

5. Rigoulot SB, Petzold HE, Williams SP, Brunner AM, and Beers EP. Populus trichocarpa clade A PP2C protein phosphatases: their stress-induced expression paterns, interactions in core abscisic acid signaling, and potential for regulation of growth and development. Plant Mol Biol. 2019:100(3):303–317. htps://doi.org/10.1007/s11103-019-00861-7

6. Zawaski C and Busov VB. Roles of Gibberellin Catabolism and Signaling in Growth and Physiological Response to Drought and Short-Day Photoperiods in Populus Trees. PLoS ONE. 2014:9(1):e86217. htps://doi.org/10.1371/journal.pone.0086217

7. Zhang Y, Zhang B, Yan D, Dong W, Yang W, Li Q, Zeng L, Wang J, Wang L, Hicks LM, et al. Two Arabidopsis cytochrome P450 monooxygenases, CYP714A1 and CYP714A2, function redundantly in plant development through gibberellin deactivation: CYP714A1 and CYP714A2 function in development. The Plant Journal. 2011:67(2):342–353. htps://doi.org/10.1111/j.1365-313X.2011.04596.x

8. Wang C, Yang Y, Wang H, Ran X, Li B, Zhang J, and Zhang H. Ectopic expression of a cytochrome P450 monooxygenase gene PtCYP714A3 from Populus trichocarpa reduces shoot growth and improves tolerance to salt stress in transgenic rice. Plant Biotechnol J. 2016a:14(9):1838–1851. htps://doi.org/10.1111/pbi.12544

Arabidopsis identifiers :

ELA: ELA1 (AT5G24910), ELA2 (AT5G24900)
