# Supplementary material for: Gene network analysis predicts the primary regulators of ABA-dependent transcriptional activation and repression in Populus roots
Source: PLoS Genet. 2026 Aug 4;22(8):e1012256. doi: 10.1371/journal.pgen.1012256 (PMC13466054; doi:10.1371/journal.pgen.1012256)
Supplement: Supplementary file 6 [file pgen.1012256.s006.pdf]

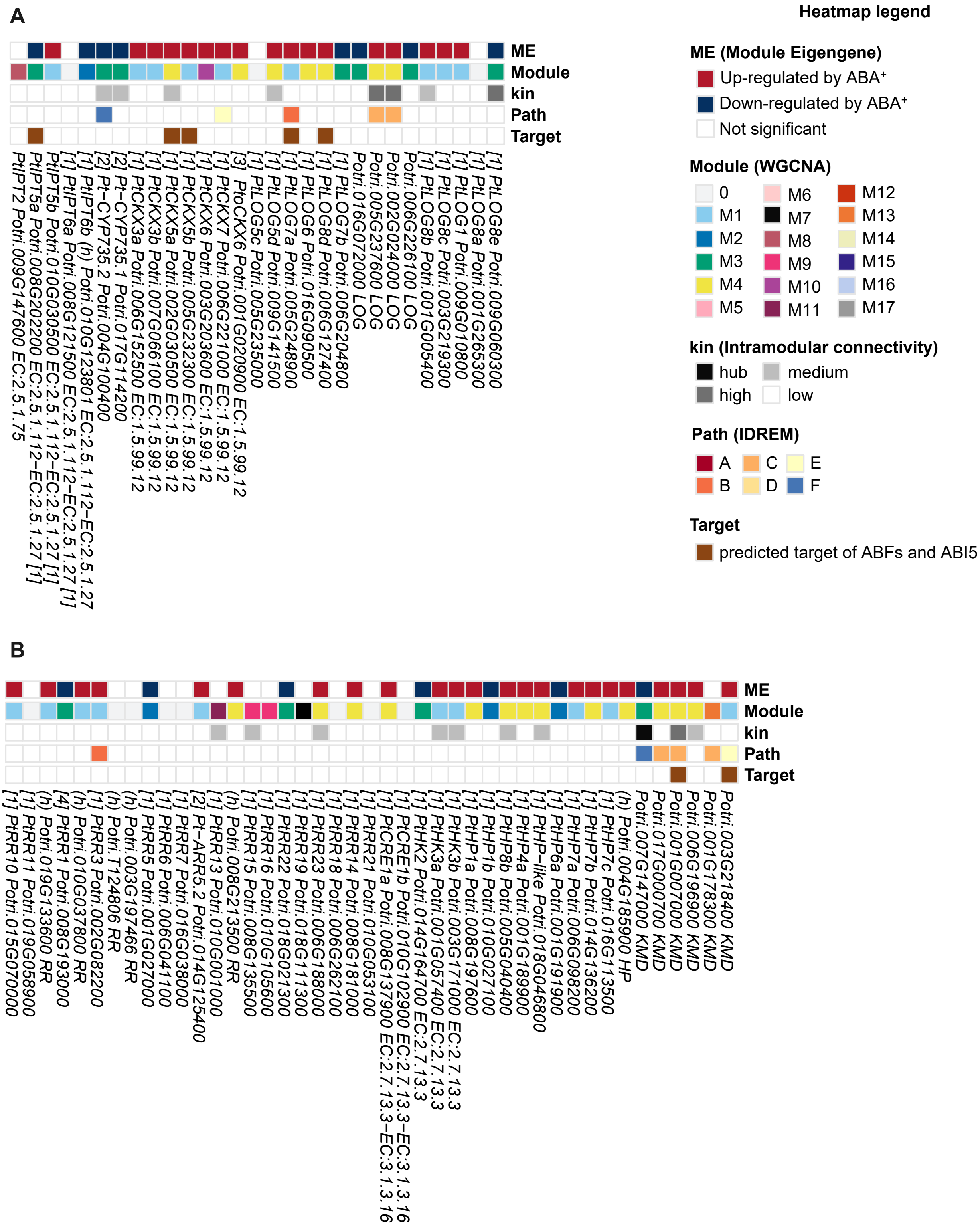

S6 Figure: Genes encoding proteins involved in (A) cytokinin metabolism and (B) cytokinin signalling.

The heatmaps summarize the main transcriptional features of WGCNA analysis, namely gene assignation to ABA-activated or ABA-inactivated modules (ME, Fig 3B), module assignation (Module) and scaled intramodular connectivity (kin), of iDREM analysis (Path), and of *in silico* prediction about ABI5 and/or ABFs gene interaction from plantregmap.gao-lab.org (Target). [number] indicates literature source listed below; (h) indicates homology-based information by querying comparative genomics databases. Only unigenes expressed in roots are shown, identifiers refer to *P. trichocarpa* V4.1 genome.

References

1. Immanen J, Nieminen K, Duchens Silva H, Rodríguez Rojas F, Meisel LA, Silva H, Albert VA, Hvidsten TR, and Helariuta Y. Characterization of cytokinin signaling and homeostasis gene families in two hardwood tree species: Populus trichocarpa and Prunus persica. BMC Genomics. 2013:14(1):885. https://doi.org/10.1186/1471-2164-14-885

2. Sjödin A, Street NR, Sandberg G, Gustafsson P, and Jansson S. The Populus Genome Integrative Explorer (PopGenIE): a new resource for exploring the Populus genome. New Phytologist. 2009:182(4):1013–1025. https://doi.org/10.1111/j.1469-8137.2009.02807.x

3. Song Y, Chen P, Xuan A, Bu C, Liu P, Ingvarsson PK, El-Kassaby YA, and Zhang D. Integration of genome wide association studies and co-expression networks reveal roles of PtoWRKY 42-PtoUGT76C1-1 in trans -zeatin metabolism and cytokinin sensitivity in poplar. New Phytol. 2021:231(4):1462–1477. https://doi.org/10.1111/nph.17469

4. Immanen J, Nieminen K, Smolander O-P, Kojima M, Alonso Serra J, Koskinen P, Zhang J, Elo A, Mähönen AP, Street N, et al. Cytokinin and Auxin Display Distinct but Interconnected Distribution and Signaling Profiles to Stimulate Cambial Activity. Current Biology. 2016:26(15):1990–1997. https://doi.org/10.1016/j.cub.2016.05.053
