# Supplementary material for: Gene network analysis predicts the primary regulators of ABA-dependent transcriptional activation and repression in Populus roots
Source: PLoS Genet. 2026 Aug 4;22(8):e1012256. doi: 10.1371/journal.pgen.1012256 (PMC13466054; doi:10.1371/journal.pgen.1012256)
Supplement: Supplementary file 7 [file pgen.1012256.s007.pdf]

Cohen *et al*. Gene network analysis predicts the primary regulators of ABA-dependent transcriptional activation and repression in *Populus* roots

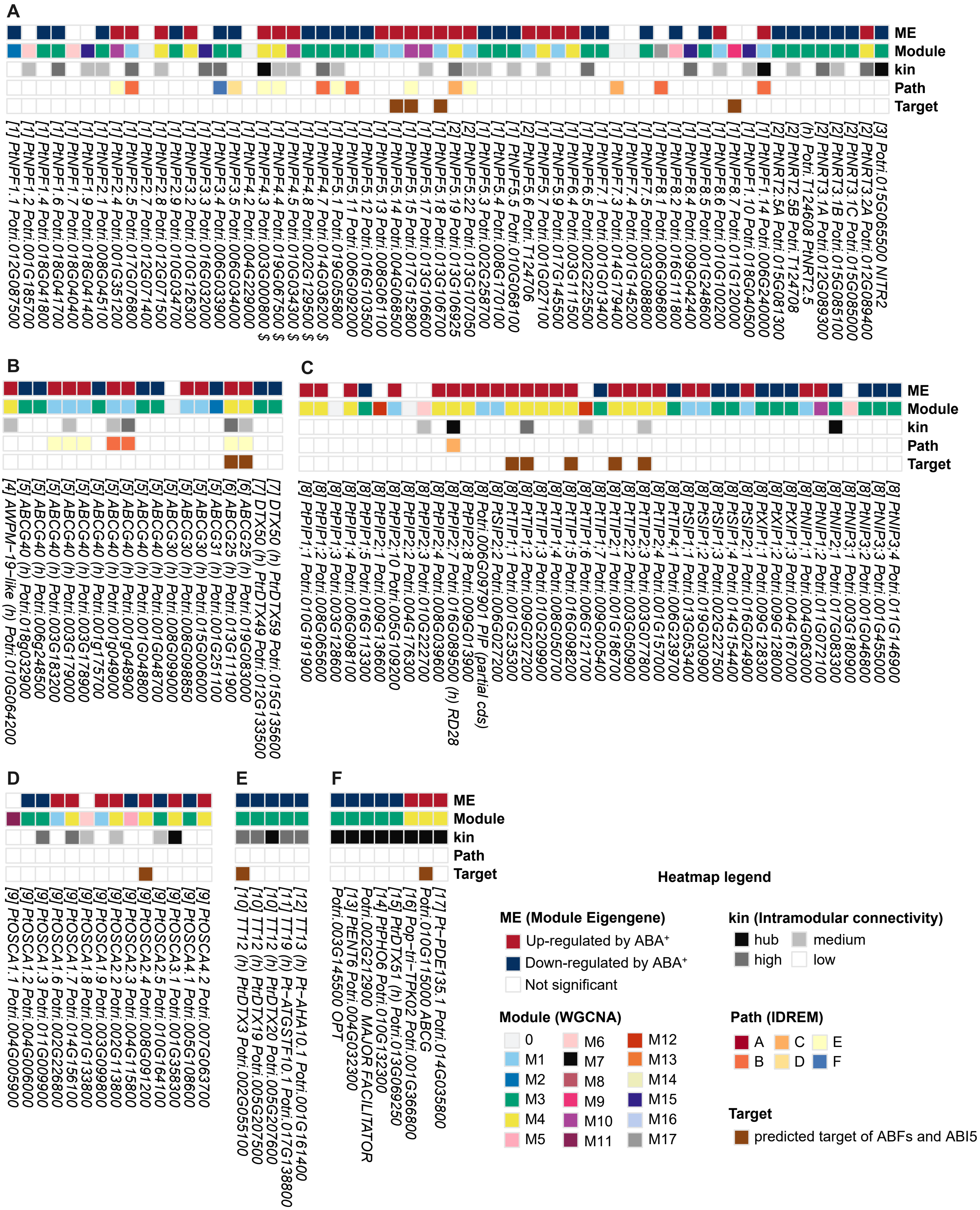

S7 Figure: Genes encoding putative transporters and channels. (A) Genes encoding putative nitrate transporters. The $ sign indicates putative ABA transporter of the PtNPF family. (B) Genes encoding other putative ABA transporters of the AWPM-19, ABCG and DTX families. (C) *Aquaporin* and (D) *OSCA* family. (E) Genes related to anthocyanin/flavonoid transport. (F) Further M3 and M4 hub genes encoding miscellaneous transporters.

The heatmaps summarize the main transcriptional features of WGCNA analysis, namely gene assignation to ABA-activated or ABA-inactivated modules (ME, Fig 3B), module assignation (Module) and scaled intramodular connectivity (kin), of iDREM analysis (Path), and of *in silico* prediction about ABI5 and/or ABFs gene interaction from plantregmap.gao-lab.org (Target). [number] indicates literature source listed below; (h) indicates homology-based information by querying comparative genomics databases. Only unigenes expressed in roots are shown, identifiers refer to *P. trichocarpa* V4.1 genome.

References

1. Léran S, Noguero M, Corratgé-Faillie C, Boursiac Y, Brachet C, and Lacombe B. Functional Characterization of the Arabidopsis Abscisic Acid Transporters NPF4.5 and NPF4.6 in Xenopus Oocytes. Front Plant Sci. 2020:11:144. https://doi.org/10.3389/fpls.2020.00144

2. Castro-Rodríguez V, Cañas RA, De La Torre FN, Pascual MB, Avila C, and Cánovas FM. Molecular fundamentals of nitrogen uptake and transport in trees. Journal of Experimental Botany. 2017:68(10):2489–2500. htps://doi.org/10.1093/jxb/erx037

3. Maeda S, Konishi M, Yanagisawa S, and Omata T. Nitrite Transport Activity of a Novel HPP Family Protein Conserved in Cyanobacteria and Chloroplasts. Plant and Cell Physiology. 2014:55(7):1311–1324. htps://doi.org/10.1093/pcp/pcu075

4. Yao L, Cheng X, Gu Z, Huang W, Li S, Wang L, Wang Y-F, Xu P, Ma H, and Ge X. The AWPM-19 Family Protein OsPM1 Mediates Abscisic Acid Influx and Drought Response in Rice. Plant Cell. 2018:30(6):1258–1276. htps://doi.org/10.1105/tpc.17.00770

5. Kang J, Yim S, Choi H, Kim A, Lee KP, Lopez-Molina L, Martinoia E, and Lee Y. Abscisic acid transporters cooperate to control seed germination. Nat Commun. 2015:6:8113. htps://doi.org/10.1038/ncomms9113

6. Kuromori T and Shinozaki K. ABA transport factors found in Arabidopsis ABC transporters. Plant Signaling & Behavior. 2010:5(9):1124–1126. htps://doi.org/10.4161/psb.5.9.12566

7. Zhang H, Zhu H, Pan Y, Yu Y, Luan S, and Li L. A DTX/MATE-Type Transporter Facilitates Abscisic Acid Efflux and Modulates ABA Sensitivity and Drought Tolerance in Arabidopsis. Molecular Plant. 2014:7(10):1522–1532. htps://doi.org/10.1093/mp/ssu063

8. Gupta A and Sankararamakrishnan R. Genome-wide analysis of major intrinsic proteins in the tree plant Populus trichocarpa: Characterization of XIP subfamily of aquaporins from evolutionary perspective. BMC Plant Biol. 2009:9(1):134. htps://doi.org/10.1186/1471-2229-9-134

9. Hartmann FP, Tinturier E, Julien J-L, and Leblanc-Fournier N. Between Stress and Response: Function and Localization of Mechanosensitive Ca2+ Channels in Herbaceous and Perennial Plants. IJMS. 2021:22(20):11043. https://doi.org/10.3390/ijms222011043

10. Marinova K, Pourcel L, Weder B, Schwarz M, Barron D, Routaboul J-M, Debeaujon I, and Klein M. The Arabidopsis MATE transporter TT12 acts as a vacuolar flavonoid/H+ -antiporter active in proanthocyanidin-accumulating cells of the seed coat. Plant Cell. 2007:19(6):2023–2038. htps://doi.org/10.1105/tpc.106.046029

11. Kitamura S, Shikazono N, and Tanaka A. TRANSPARENT TESTA 19 is involved in the accumulation of both anthocyanins and proanthocyanidins in Arabidopsis. The Plant Journal. 2004:37(1):104–114. htps://doi.org/10.1046/j.1365-313X.2003.01943

12. Appelhagen I, Nordholt N, Seidel T, Spelt K, Koes R, Quatrochio F, Sagasser M, and Weisshaar B. TRANSPARENT TESTA 13 is a tonoplast P 3A -ATPase required for vacuolar deposition of proanthocyanidins in Arabidopsis thaliana seeds. Plant J. 2015:82(5):840–849. htps://doi.org/10.1111/tpj.12854

13. Immanen J, Nieminen K, Smolander O-P, Kojima M, Alonso Serra J, Koskinen P, Zhang J, Elo A, Mähönen AP, Street N, et al. Cytokinin and Auxin Display Distinct but Interconnected Distribution and Signaling Profiles to Stimulate Cambial Activity. Current Biology. 2016:26(15):1990–1997. htps://doi.org/10.1016/j.cub.2016.05.053

14. Zhang C, Meng S, Li M, and Zhao Z. Genomic Identification and Expression Analysis of the Phosphate Transporter Gene Family in Poplar. Front Plant Sci. 2016:7. htps://doi.org/10.3389/fpls.2016.01398

15. Li N, M eng H , Xing H , Liang L , Zhao X, a nd Luo K . Genome-wide analysis of MATE transporters and molecular characterization of aluminum resistance in Populus. Journal of Experimental Botany. 2017:68(20):5669–5683. htps://doi.org/10.1093/jxb/erx370

16. Gomez-Porras JL, Riaño-Pachón DM, Benito B, Haro R, Sklodowski K, Rodríguez-Navarro A, and Dreyer I. Phylogenetic Analysis of K+ Transporters in Bryophytes, Lycophytes, and Flowering Plants Indicates a Specialization of Vascular Plants. Front Plant Sci. 2012:3. htps://doi.org/10.3389/fpls.2012.00167

17. Sjödin A, Street NR, Sandberg G, Gustafsson P, and Jansson S. The Populus Genome Integrative Explorer (PopGenIE): a new resource for exploring the Populus genome. New Phytologist. 2009:182(4):1013–1025. htps://doi.org/10.1111/j.1469-8137.2009.02807.x

Arabidopsis identifiers :

ABCG25 (AT1G71960); ABCG30 (AT4G15230); ABCG31 (AT2G29940); ABCG40 (AT1G15520); AWPM-19-like: OsPM1(Os05g0381400); DTX50 (AT5G52050); RD28 (AT2G37180); TT12 (AT3G59030); TT13 (AT1G17260); TT19 (AT5G17220); TTG1 (AT5G24520)
