# Supplementary material for: Gene network analysis predicts the primary regulators of ABA-dependent transcriptional activation and repression in Populus roots
Source: PLoS Genet. 2026 Aug 4;22(8):e1012256. doi: 10.1371/journal.pgen.1012256 (PMC13466054; doi:10.1371/journal.pgen.1012256)
Supplement: Supplementary file 8 [file pgen.1012256.s008.pdf]

Cohen *et al*. Gene network analysis predicts the primary regulators of ABA-dependent transcriptional activation and repression in *Populus* roots

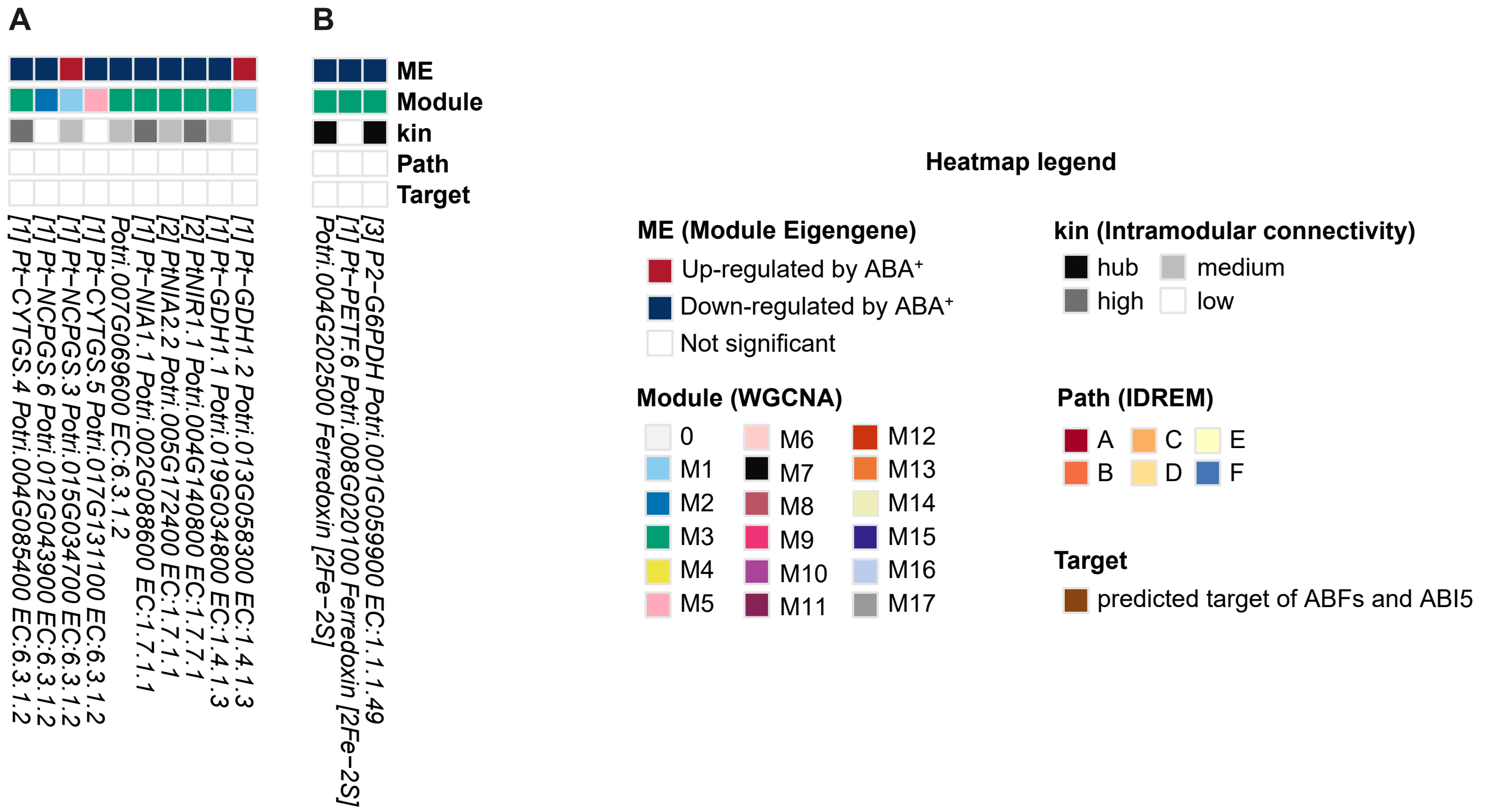

S8 Figure: Genes encoding proteins putatively involved in (A) nitrogen metabolism and in (B) oxidative pentose phosphate (OPP) pathway.

The heatmaps summarize the main transcriptional features of WGCNA analysis, namely gene assignation to ABA-activated or ABA-inactivated modules (ME, Fig 3B), module assignation (Module) and scaled intramodular connectivity (kin), of iDREM analysis (Path), and of *in silico* prediction about ABI5 and/or ABFs gene interaction from plantregmap.gao-lab.org (Target). [number] indicates literature source listed below. Only unigenes expressed in roots are shown, identifiers refer to *P. trichocarpa* V4.1 genome.

References

1. Sjödin A, Street NR, Sandberg G, Gustafsson P, and Jansson S. The Populus Genome Integrative Explorer (PopGenIE): a new resource for exploring the Populus genome. New Phytologist. 2009:182(4):1013–1025. https://doi.org/10.1111/j.1469-8137.2009.02807.x

2. Euring D, Bai H, Janz D, and Polle A. Nitrogen-driven stem elongation in poplar is linked with wood modification and gene clusters for stress, photosynthesis and cell wall formation. BMC Plant Biol. 2014:14(1):391. https://doi.org/10.1186/s12870-014-0391-3

3. Cardi M, Zaffagnini M, De Lillo A, Castiglia D, Chibani K, Gualberto JM, Rouhier N, Jacquot J-P, and Esposito S. Plastidic P2 glucose-6P dehydrogenase from poplar is modulated by thioredoxin mtype: Distinct roles of cysteine residues in redox regulation and NADPH inhibition. Plant Science. 2016:252:257–266. https://doi.org/10.1016/j.plantsci.2016.08.003
