# Supplementary material for: Gene network analysis predicts the primary regulators of ABA-dependent transcriptional activation and repression in Populus roots
Source: PLoS Genet. 2026 Aug 4;22(8):e1012256. doi: 10.1371/journal.pgen.1012256 (PMC13466054; doi:10.1371/journal.pgen.1012256)
Supplement: Supplementary file 9 [file pgen.1012256.s009.pdf]

Cohen *et al*. Gene network analysis predicts the primary regulators of ABA-dependent transcriptional activation and repression in *Populus* roots

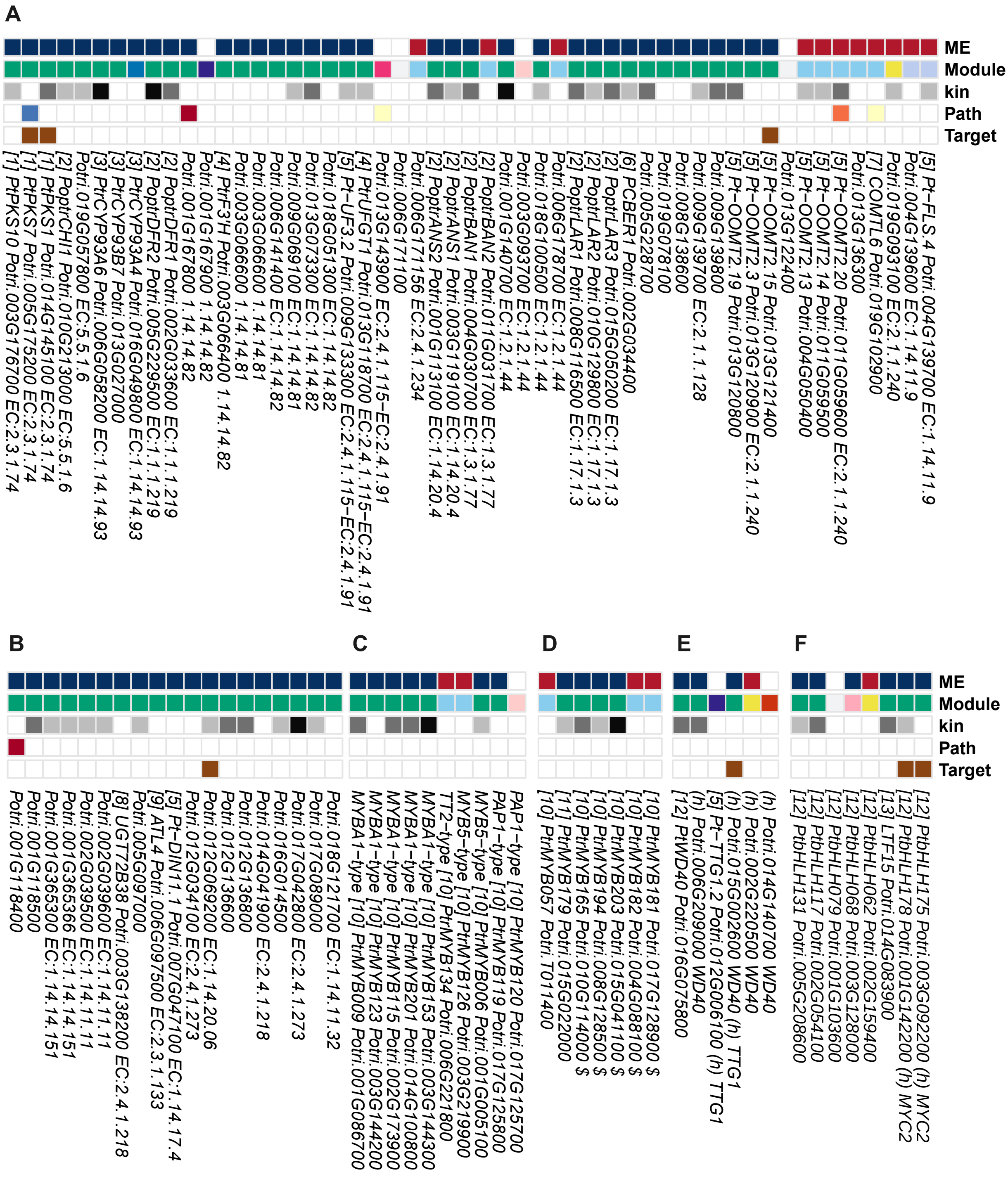

S9 Figure: Genes encoding proteins putatively involved in (A-B) flavonoid metabolism. (B) Further genes contributing to M3 enrichment in "Bin16.8: secondary metabolism.flavonoids". (C-F) Genes related to the regulation of anthocyanin/proanthocyanidin synthesis. R2R3-MYB transcription factors acting as (C) activators and (D) repressors. The $ sign indicates R2R3-MYB Subfamily S4 (Du et al. 2015). Genes encoding other putative members of the regulatory MBW complex belonging to (E) WD40 repeat family protein and (F) bHLH transcription factor family.

The heatmaps summarize the main transcriptional features of WGCNA analysis, namely gene assignation to ABA-activated or ABA-inactivated modules (ME, Fig 3B), module assignation (Module) and scaled intramodular connectivity (kin), of iDREM analysis (Path), and of *in silico* prediction about ABI5 and/or ABFs gene interaction from plantregmap.gao-lab.org (Target). [number] indicates literature source listed below; (h) indicates homology-based information by querying comparative genomics databases. Only unigenes expressed in roots are shown, identifiers refer to *P. trichocarpa* V4.1 genome.

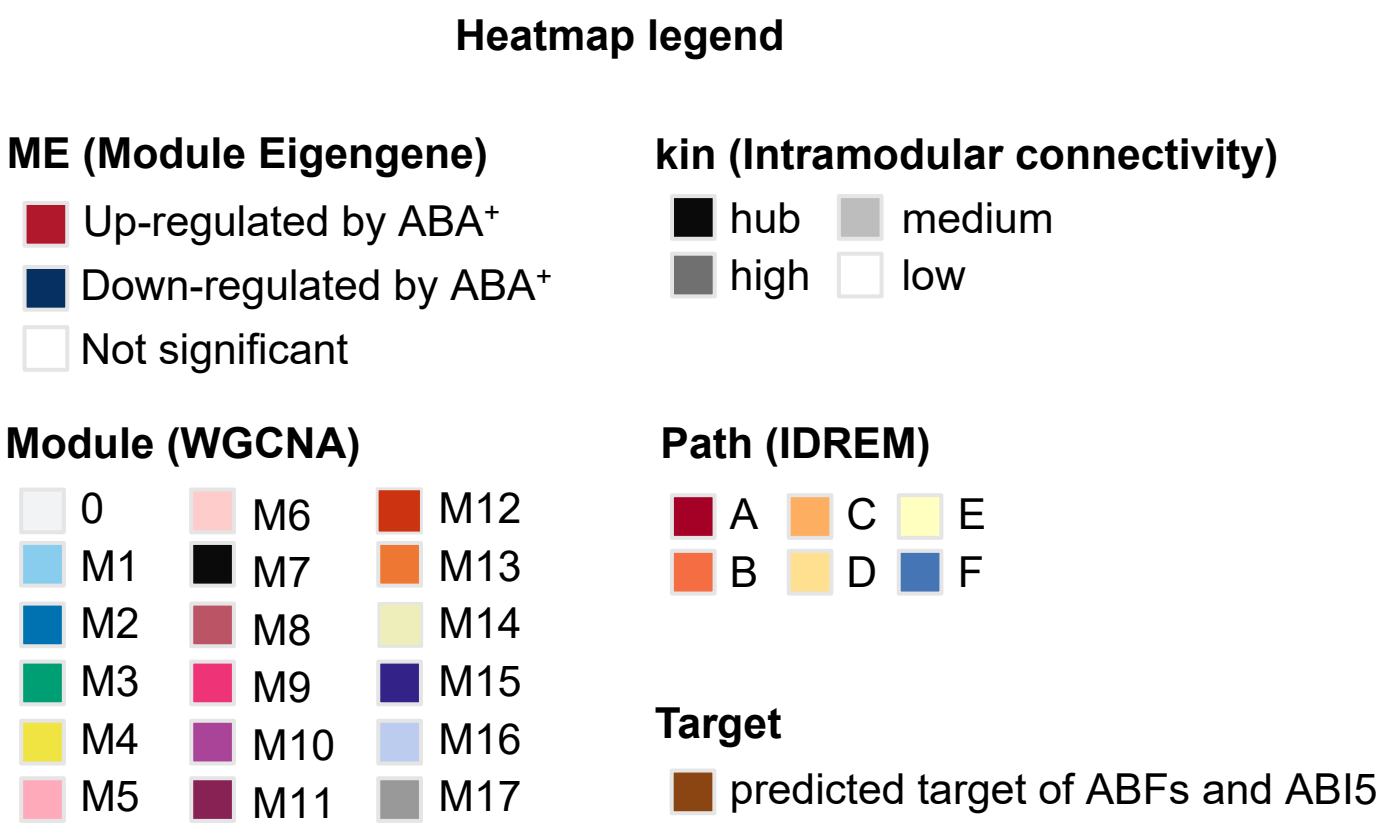

References

1. Su X, Sun X, Cheng X, Wang Y, Abdullah M, Li M, Li D, Gao J, Cai Y, and Lin Y. Comparative genomic analysis of the PKS genes in five species and expression analysis in upland coton. PeerJ. 2017:5:e3974. htps://doi.org/10.7717/peerj.3974

2. Tsai C, Harding SA, Tschaplinski TJ, Lindroth RL, and Yuan Y. Genome-wide analysis of the structural genes regulating defense phenylpropanoid metabolism in Populus. New Phytologist. 2006:172(1):47– 62. htps://doi.org/10.1111/j.1469-8137.2006.01798.x

3. Du H, Ran F, Dong H-L, Wen J, Li J-N, and Liang Z. Genome-Wide Analysis, Classification, Evolution, and Expression Analysis of the Cytochrome P450 93 Family in Land Plants. PLoS ONE. 2016:11(10):e0165020. htps://doi.org/10.1371/journal.pone.0165020

4. Zhuang W, Wang H, Liu T, Wang T, Zhang F, Shu X, Zhai H, and Wang Z. Integrated physiological and genomic analysis reveals structural variations and expression paterns of candidate genes for colored- and green-leaf poplar. Sci Rep. 2019:9(1):11150. htps://doi.org/10.1038/s41598-019-47681-9

5. Sjödin A, Street NR, Sandberg G, Gustafsson P, and Jansson S. The Populus Genome Integrative Explorer (PopGenIE): a new resource for exploring the Populus genome. New Phytologist. 2009:182(4):1013–1025. htps://doi.org/10.1111/j.1469-8137.2009.02807.x

6. Obudulu O, Bygdell J, Sundberg B, Moritz T, Hvidsten TR, Trygg J, and Wingsle G. Quantitative proteomics reveals protein profiles underlying major transitions in aspen wood development. BMC Genomics. 2016:17(1):119. htps://doi.org/10.1186/s12864-016-2458-z

7. Hamberger B, Ellis M, Friedmann M, De Azevedo Souza C, Barbazuk B, and Douglas CJ. Genome-wide analyses of phenylpropanoid-related genes in Populus trichocarpa, Arabidopsis thaliana, and Oryza sativa: the Populus lignin toolbox and conservation and diversification of angiosperm gene familiesThis article is one of a selection of papers published in the Special Issue on Poplar Research in Canada. Can J Bot. 2007:85(12):1182–1201. htps://doi.org/10.1139/B07-098

8. Speeckaert N, Adamou NM, Hassane HA, Baldacci-Cresp F, Mol A, Goeminne G, Boerjan W, Duez P, Hawkins S, Neutelings G, et al. Characterization of the UDP-glycosyltransferase UGT72 Family in Poplar and Identification of Genes Involved in the Glycosylation of Monolignols. IJMS. 2020:21(14):5018. htps://doi.org/10.3390/ijms21145018

9. Tuominen LK, Johnson VE, and Tsai C-J. Differential phylogenetic expansions in BAHD acyltransferases across five angiosperm taxa and evidence of divergent expression among Populus paralogues. BMC Genomics. 2011:12(1):236. htps://doi.org/10.1186/1471-2164-12-236

10. Chai G, Wang Z, Tang X, Yu L, Qi G, Wang D, Yan X, Kong Y, and Zhou G. R2R3-MYB gene pairs in Populus: evolution and contribution to secondary wall formation and flowering time. Journal of Experimental Botany. 2014:65(15):4255–4269. htps://doi.org/10.1093/jxb/eru196

11. Wilkins O, Nahal H, Foong J, Provart NJ, and Campbell MM. Expansion and Diversification of the Populus R2R3-MYB Family of Transcription Factors. Plant Physiology. 2009:149(2):981–993. htps://doi.org/10.1104/pp.108.132795

12. Gesell A, Yoshida K, Tran LT, and Constabel CP. Characterization of an apple TT2-type R2R3 MYB transcription factor functionally similar to the poplar proanthocyanidin regulator PtMYB134. Planta. 2014:240(3):497–511. htps://doi.org/10.1007/s00425-014-2098-y

13. Gui J, Luo L, Zhong Y, Sun J, Umezawa T, and Li L. Phosphorylation of LTF1, an MYB Transcription Factor in Populus, Acts as a Sensory Switch Regulating Lignin Biosynthesis in Wood Cells. Molecular Plant. 2019:12(10):1325–1337. htps://doi.org/10.1016/j.molp.2019.05.008

Arabidopsis identifiers :

MYC2 (AT1G32640) ; TTG1 (AT5G24520)
