# Supplementary material for: Gene network analysis predicts the primary regulators of ABA-dependent transcriptional activation and repression in Populus roots
Source: PLoS Genet. 2026 Aug 4;22(8):e1012256. doi: 10.1371/journal.pgen.1012256 (PMC13466054; doi:10.1371/journal.pgen.1012256)
Supplement: Supplementary file 10 [file pgen.1012256.s010.pdf]

Cohen *et al*. Gene network analysis predicts the primary regulators of ABA-dependent transcriptional activation and repression in *Populus* roots

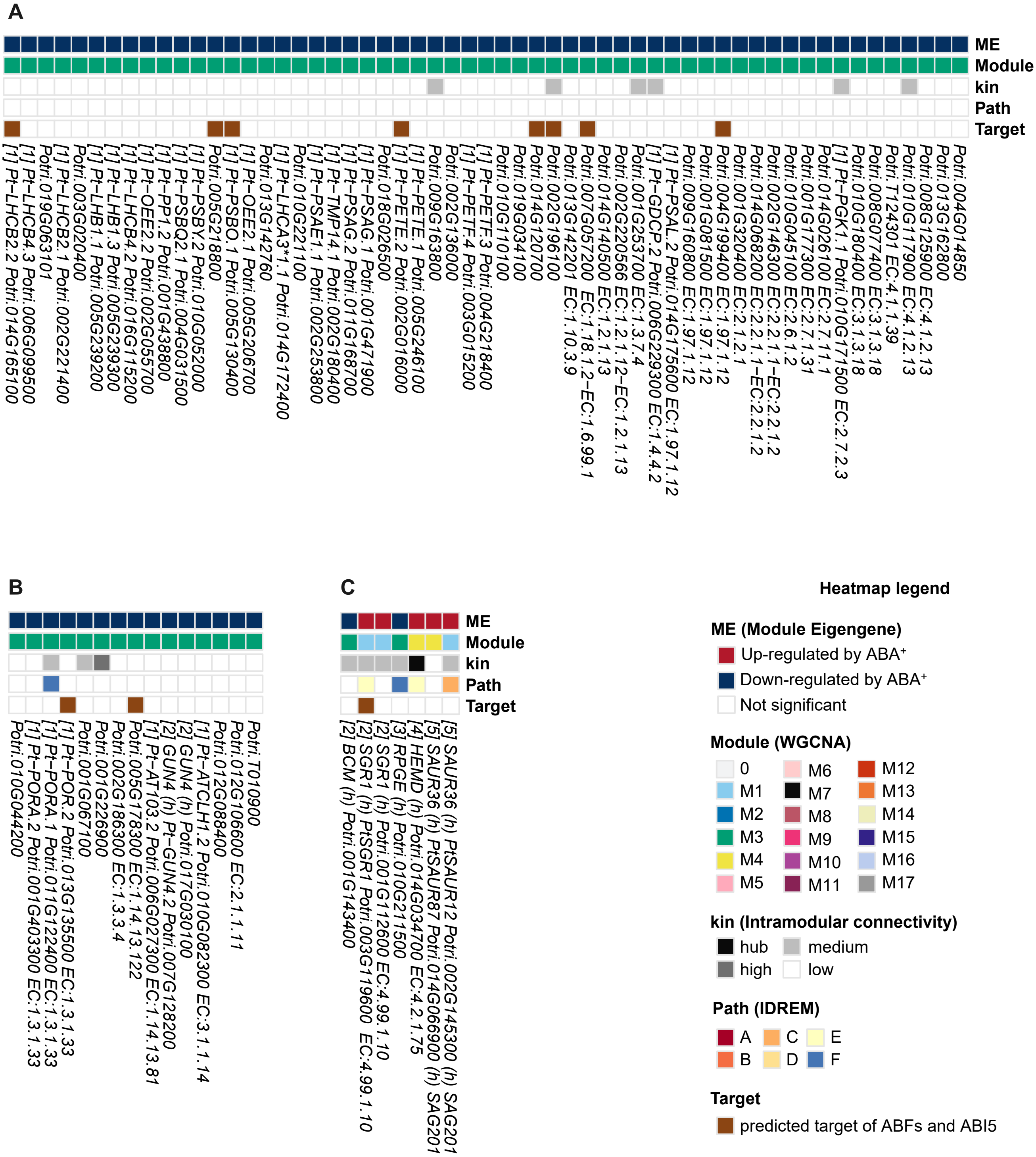

S10 Figure: Genes contributing to M3 enrichments in (A) "Bin1: photosystem" and (B) "Bin19: tetrapyrrole synthesis". (C) Poplar homologs and orthologs of key regulatory genes acting on chlorophyll homeostasis in Arabidopsis and SAG201 homologs.

The heatmaps summarize the main transcriptional features of WGCNA analysis, namely gene assignation to ABA-activated or ABA-inactivated modules (ME, Fig 3B), module assignation (Module) and scaled intramodular connectivity (kin), of iDREM analysis (Path), and of *in* silico prediction about ABI5 and/or ABFs gene interaction from plantregmap.gao-lab.org (Target). [number] indicates literature source listed below; (h) indicates homology-based information by querying comparative genomics databases. Only unigenes expressed in roots are shown, identifiers refer to *P. trichocarpa* V4.1 genome.

References

1. Sjödin A, Street NR, Sandberg G, Gustafsson P, and Jansson S. The Populus Genome Integrative Explorer (PopGenIE): a new resource for exploring the Populus genome. New Phytologist. 2009:182(4):1013–1025. htps://doi.org/10.1111/j.1469-8137.2009.02807.x

2. Wang P, Richter AS, Kleeberg JRW, Geimer S, and Grimm B. Post-translational coordination of chlorophyll biosynthesis and breakdown by BCMs maintains chlorophyll homeostasis during leaf development. Nat Commun. 2020a:11(1):1254. htps://doi.org/10.1038/s41467-020-14992-9

3. Tachibana R, Abe S, Marugami M, Yamagami A, Akema R, Ohashi T, Nishida K, Nosaki S, Miyakawa T, Tanokura M, et al. BPG4 regulates chloroplast development and homeostasis by suppressing GLK transcription factors and involving light and brassinosteroid signaling. Nat Commun. 2024:15(1). htps://doi.org/10.1038/s41467-023-44492-5

4. Tan F-C, Cheng Q, Saha K, Heinemann IU, Jahn M, Jahn D, and Smith AG. Identification and characterization of theArabidopsisgene encoding the tetrapyrrole biosynthesis enzyme uroporphyrinogen III synthase. Biochemical Journal. 2008:410(2):291–299. htps://doi.org/10.1042/bj20070770

5. Zhang K and Gan S-S. An Abscisic Acid-AtNAP Transcription Factor-SAG113 Protein Phosphatase 2C Regulatory Chain for Controlling Dehydration in Senescing Arabidopsis Leaves. Plant Physiology. 2012:158(2):961–969. htps://doi.org/10.1104/pp.111.190876

Arabidopsis identifiers :

BCM: BCM1 (AT2G35260), BCM2 (AT4G17840); GUN4 (AT3G59400); HEMD (AT2G26540); RPGE: RPGE2 (AT3G55240), RPGE3 (AT3G28990); SAUR36 (AT2G45210); SGR1 (AT4G22920)
