# Supplementary material for: Gene network analysis predicts the primary regulators of ABA-dependent transcriptional activation and repression in Populus roots
Source: PLoS Genet. 2026 Aug 4;22(8):e1012256. doi: 10.1371/journal.pgen.1012256 (PMC13466054; doi:10.1371/journal.pgen.1012256)
Supplement: Supplementary file 11 [file pgen.1012256.s011.pdf]

Cohen *et al*. Gene network analysis predicts the primary regulators of ABA-dependent transcriptional activation and repression in *Populus* roots

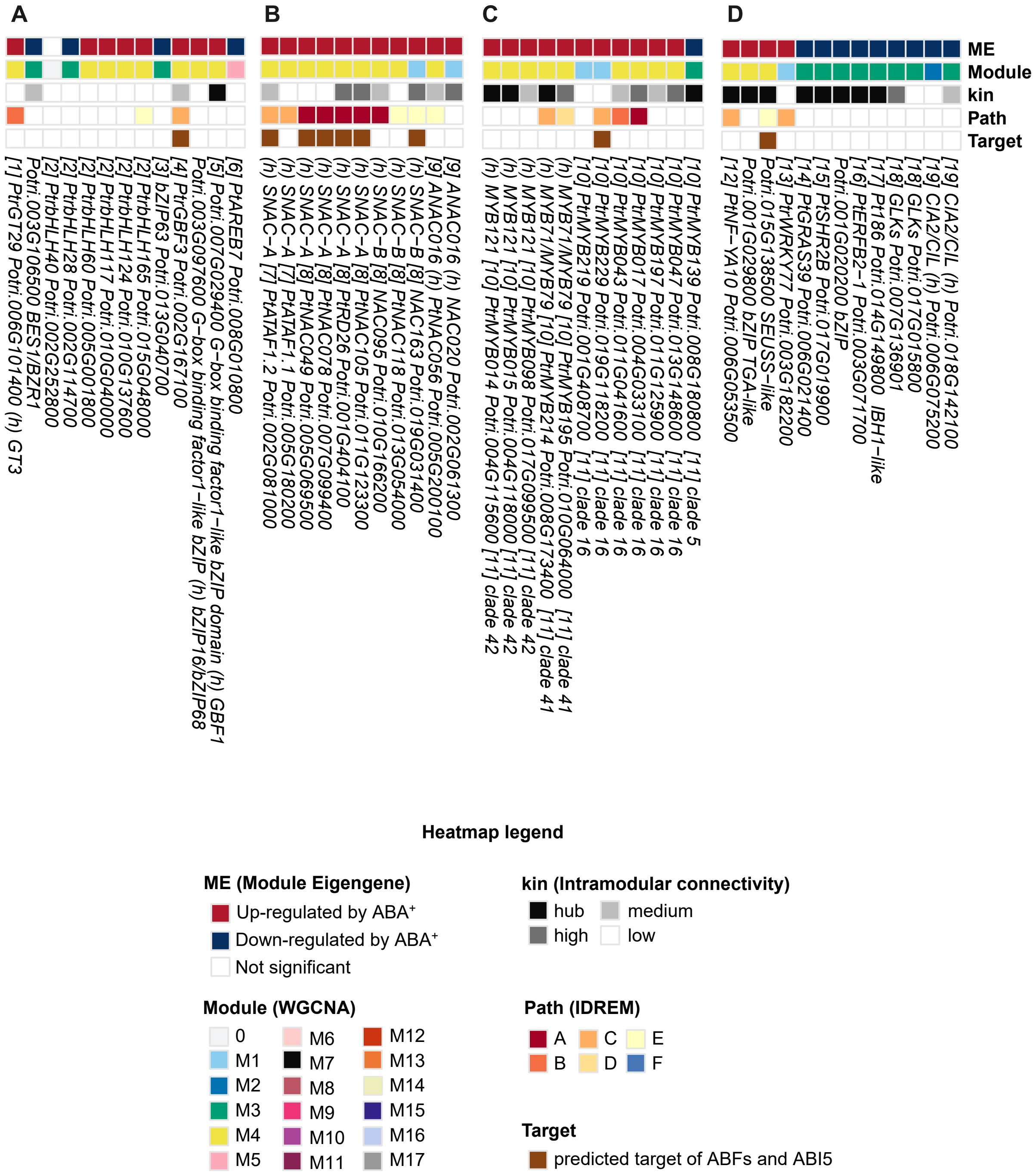

S11 Figure: Genes encoding further transcription factors (A) predicted by iDREM or putatively involved in ABA signal amplification and diversification, including (B) Stress-responsive NAC (SNAC) and other NAC transcription factors, (C) R2R3-MYBs and (D) transcription factors from miscellaneous families.

The heatmaps summarize the main transcriptional features of WGCNA analysis, namely gene assignation to ABA-activated or ABA-inactivated modules (ME, Fig 3B), module assignation (Module) and scaled intramodular connectivity (kin), of iDREM analysis (Path), and of *in silico* prediction about ABI5 and/or ABFs gene interaction from plantregmap.gao-lab.org (Target). [number] indicates literature source listed below; (h) indicates homology-based information by querying comparative genomics databases. Only unigenes expressed in roots are shown, identifiers refer to *P. trichocarpa* V4.1 genome.

References

1. Wang Z, Liu Q, Wang H, Zhang H, Xu X, Li C, and Yang C. Comprehensive analysis of trihelix genes and their expression under biotic and abiotic stresses in Populus trichocarpa. Sci Rep. 2016b:6(1):36274. htps://doi.org/10.1038/srep36274

2. Zhao K, Li S, Yao W, Zhou B, Li R, and Jiang T. Characterization of the basic helix–loop–helix gene family and its tissue-differential expression in response to salt stress in poplar. PeerJ. 2018:6:e4502. htps://doi.org/10.7717/peerj.4502

3. Mair A, Pedrotti L, Wurzinger B, Anrather D, Simeunovic A, Weiste C, Valerio C, Dietrich K, Kirchler T, Nägele T, et al. SnRK1-triggered switch of bZIP63 dimerization mediates the low-energy response in plants. eLife. 2015:4:e05828. htps://doi.org/10.7554/eLife.05828

4. Li S, Lin Y-CJ, Wang P, Zhang B, Li M, Chen S, Shi R, Tunlaya-Anukit S, Liu X, Wang Z, et al. The AREB1 Transcription Factor Influences Histone Acetylation to Regulate Drought Responses and Tolerance in Populus trichocarpa. Plant Cell. 2019:31(3):663–686. htps://doi.org/10.1105/tpc.18.00437

5. Zhao K, Chen S, Yao W, Cheng Z, Zhou B, and Jiang T. Genome-wide analysis and expression profile of the bZIP gene family in poplar. BMC Plant Biol. 2021:21(1):122. htps://doi.org/10.1186/s12870-021-02879-w

6. Ji L, Wang J, Ye M, Li Y, Guo B, Chen Z, Li H, and An X. Identification and Characterization of the Populus AREB/ABF Subfamily. Journal of Integrative Plant Biology. 2013:55(2):177–186. htps://doi.org/10.1111/j.1744-7909.2012.01183.x

7. Wang H, Wang M, and Cheng Q. Capturing the Alternative Cleavage and Polyadenylation Sites of 14 NAC Genes in Populus Using a Combination of 3’-RACE and High-Throughput Sequencing. Molecules. 2018:23(3):608. htps://doi.org/10.3390/molecules23030608

8. Wang H-L, Zhang Y, Wang T, Yang Q, Yang Y, Li Z, Li B, Wen X, Li W, Yin W, et al. An alternative splicing variant of PtRD26 delays leaf senescence by regulating multiple NAC transcription factors in Populus. The Plant Cell. 2021b:33(5):1594–1614. htps://doi.org/10.1093/plcell/koab046

9. Sakuraba Y, Han S-H, Lee S-H, Hörtensteiner S, and Paek N-C. Arabidopsis NAC016 promotes chlorophyll breakdown by directly upregulating STAYGREEN1 transcription. Plant Cell Rep. 2016:35(1):155–166. htps://doi.org/10.1007/s00299-015-1876-8

10. Chai G, Wang Z, Tang X, Yu L, Qi G, Wang D, Yan X, Kong Y, and Zhou G. R2R3-MYB gene pairs in Populus: evolution and contribution to secondary wall formation and flowering time. Journal of Experimental Botany. 2014:65(15):4255–4269. htps://doi.org/10.1093/jxb/eru196

11. Wilkins O, Nahal H, Foong J, Provart NJ, and Campbell MM. Expansion and Diversification of the Populus R2R3-MYB Family of Transcription Factors. Plant Physiology. 2009:149(2):981–993. htps://doi.org/10.1104/pp.108.132795

12. Liu R, Wu M, Liu H, Gao Y, Chen J, Yan H, and Xiang Y. Genome-wide identification and expression analysis of the NF-Y transcription factor family in Populus. Physiol Plantarum. 2021:171(3):309–327. htps://doi.org/10.1111/ppl.13084

13. Jiang Y, Tong S , C hen N , L iu B, B ai Q , C hen Y, B i H , Z hang Z , Lou S , Tang H , et a l. The PalWRKY77 transcription factor negatively regulates salt tolerance and abscisic acid signaling in Populus. Plant J. 2021:105(5):1258–1273. htps://doi.org/10.1111/tpj.15109

14. Liu X and Widmer A. Genome-wide Comparative Analysis of the GRAS Gene Family in Populus, Arabidopsis and Rice. Plant Mol Biol Rep. 2014:32(6):1129–1145.htps://doi.org/10.1007/s11105-014-0721-5

15. Miguel A, Milhinhos A, Novák O, Jones B, and Miguel CM. The SHORT-ROOT -like gene PtSHR2B is involved in Populus phellogen activity. EXBOTJ. 2016:67(5):1545–1555. htps://doi.org/10.1093/jxb/erv547

16. Zhuang J, Cai B, Peng R-H, Zhu B, Jin X-F, Xue Y, Gao F, Fu X-Y, Tian Y-S, Zhao W, et al. Genome-wide analysis of the AP2/ERF gene family in Populus trichocarpa. Biochemical and Biophysical Research Communications. 2008:371(3):468–474. htps://doi.org/10.1016/j.bbrc.2008.04.087

17. Carretero-Paulet L, Galstyan A, Roig-Villanova I, Martinez-García JF, Bilbao-Castro JR, and Robertson DL. Genome-Wide Classification and Evolutionary Analysis of the bHLH Family of Transcription Factors in Arabidopsis, Poplar, Rice, Moss, and Algae. Plant Physiology. 2010:153(3):1398–1412. htps://doi.org/10.1104/pp.110.153593

18. Waters MT, Wang P, Korkaric M, Capper RG, Saunders NJ, and Langdale JA. GLK Transcription Factors Coordinate Expression of the Photosynthetic Apparatus in Arabidopsis. The Plant Cell. 2009:21(4):1109–1128. htps://doi.org/10.1105/tpc.108.065250

19. Yang C, Yan W, Chang H, and Sun C. Arabidopsis CIA2 and CIL have distinct and overlapping functions in regulating chloroplast and flower development. Plant Direct. 2022:6(1). htps://doi.org/10.1002/pld3.380

Arabidopsis identifiers :

bZIP16/bZIP68: bZIP16 (AT2G35530), bZIP68 (AT1G32150); CIA2/CIL: CIA2 (AT5G57180), CIL (AT4G25990); GBF1 (AT4G36730); GLKs: GLK1 (AT2G20570), GLK2 (AT5G44190); GT3: GT3a (AT5G01380), GT3b (AT2G38250); MYB121 (AT3G30210); MYB71/MYB79: MYB71 (AT3G24310), MYB79 (AT4G13480);
