# Supplementary material for: Gene network analysis predicts the primary regulators of ABA-dependent transcriptional activation and repression in Populus roots
Source: PLoS Genet. 2026 Aug 4;22(8):e1012256. doi: 10.1371/journal.pgen.1012256 (PMC13466054; doi:10.1371/journal.pgen.1012256)
Supplement: Supplementary file 12 [file pgen.1012256.s012.pdf]

Cohen *et al*. Gene network analysis predicts the primary regulators of ABA-dependent transcriptional activation and repression in *Populus* roots

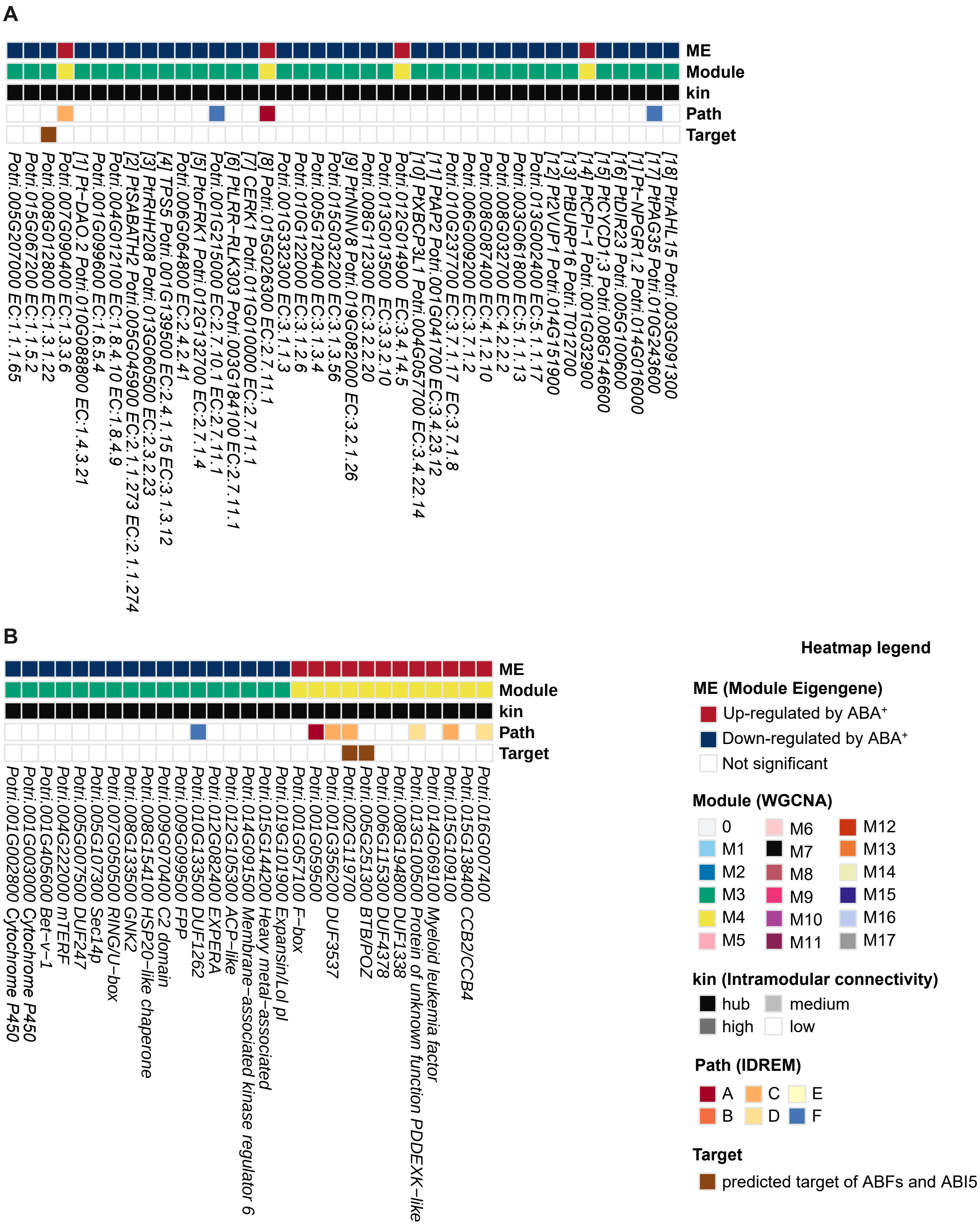

S12 Figure: M4 and M3 hub genes not currently associated with a specific pathway or process, or encoding proteins of unknown function.(A) Hub genes encoding miscellaneous enzymes and/or previously named. (B) Further hub genes with miscellaneous motif and domains.

The heatmaps summarize the main transcriptional features of WGCNA analysis, namely gene assignation to ABA-activated or ABA-inactivated modules (ME, Fig 3B), module assignation (Module) and scaled intramodular connectivity (kin), of iDREM analysis (Path), and of *in silico* prediction about ABI5 and/or ABFs gene interaction from plantregmap.gao-lab.org (Target). [number] indicates literature source listed below. Only unigenes expressed in roots are shown, identifiers refer to *P. trichocarpa* V4.1 genome.

References

1. Sjödin A, Street NR, Sandberg G, Gustafsson P, and Jansson S. The Populus Genome Integrative Explorer (PopGenIE): a new resource for exploring the Populus genome. New Phytologist. 2009:182(4):1013–1025. htps://doi.org/10.1111/j.1469-8137.2009.02807.x

2. Han X-M, Yang Q, Liu Y-J, Yang Z-L, Wang X-R, Zeng Q-Y, and Yang H-L. Evolution and Function of the Populus SABATH Family Reveal That a Single Amino Acid Change Results in a Substrate Switch. Plant and Cell Physiology. 2018:59(2):392–403. htps://doi.org/10.1093/pcp/pcx198

3. Tong G, Shen H, Cao S, Xu W, Ma X, and Cheng Y. Identification of RING-H2 Gene Candidates Related to Wood Formation in Poplar. Forests. 2019:10(8):698. htps://doi.org/10.3390/f10080698

4. Wang R, Reng M, Tian S, Liu C, Cheng H, Liu Y, Zhang H, Saqib M, Wei H, and Wei Z. Transcriptome-wide identification and characterization of microRNAs in diverse phases of wood formation in Populus trichocarpa. G3 Genes|Genomes|Genetics. 2021c:11(8):jkab195. htps://doi.org/10.1093/g3journal/jkab195

5. Xu W, Zhao Y, Chen S, Xie J, and Zhang D. Evolution and Functional Divergence of the Fructokinase Gene Family in Populus. Front Plant Sci. 2020:11:484. htps://doi.org/10.3389/fpls.2020.00484

6. Zan Y, J i Y, Z hang Y, Yang S , S ong Y, a nd Wang J . Genome-wide identification, characterization and expression analysis of populusleucine-rich repeat receptor-like protein kinase genes. BMC Genomics. 2013:14(1):318. htps://doi.org/10.1186/1471-2164-14-318

7. Miyata K, Kozaki T, Kouzai Y, Ozawa K, Ishii K, Asamizu E, Okabe Y, Umehara Y, Miyamoto A, Kobae Y, et al. The Bifunctional Plant Receptor, OsCERK1, Regulates Both Chitin-Triggered Immunity and Arbuscular Mycorrhizal Symbiosis in Rice. Plant and Cell Physiology. 2014:55(11):1864–1872. htps://doi.org/10.1093/pcp/pcu129

8. Xing S, Li M, and Liu P. Evolution of S-domain receptor-like kinases in land plants and origination of S-locus receptor kinases in Brassicaceae. BMC Evol Biol. 2013:13(1):69. htps://doi.org/10.1186/1471-2148-13-69

9. Chen Z, G ao K, S u X, R ao P, and A n X . Genome-Wide Identification of the Invertase Gene Family in Populus. PLoS ONE. 2015:10(9):e0138540. htps://doi.org/10.1371/journal.pone.0138540

10. Zou Z, Xie G, and Yang L. Papain-like cysteine protease encoding genes in rubber (Hevea brasiliensis): comparative genomics, phylogenetic, and transcriptional profiling analysis. Planta. 2017:246(5):999–1018. htps://doi.org/10.1007/s00425-017-2739-z

11. Cao S, Guo M, Wang C, Xu W, Shi T, Tong G, Zhen C, Cheng H, Yang C, Elsheery NI, et al. Genome-wide characterization of aspartic protease (AP) gene family in Populus trichocarpa and identification of the potential PtAPs involved in wood formation. BMC Plant Biol. 2019:19(1):276. htps://doi.org/10.1186/s12870-019-1865-0

12. Grienenberger E and Douglas CJ. Arabidopsis VASCULAR-RELATED UNKNOWN PROTEIN1 Regulates Xylem Development and Growth by a Conserved Mechanism That Modulates Hormone Signaling. Plant Physiology. 2014:164(4):1991–2010. htps://doi.org/10.1104/pp.114.236406

13. Shao Y, Wei G, Wang L, Dong Q, Zhao Y, Chen B, and Xiang Y. Genome-Wide Analysis of BURP Domain-Containing Genes in Populus trichocarpa. Journal of Integrative Plant Biology. 2011:53: 743-755. htps://doi.org/10.1111/j.1744-7909.2011.01068.x

14. Martinez M and Diaz I. The origin and evolution of plant cystatins and their target cysteine proteinases indicate a complex functional relationship. BMC Evol Biol. 2008:8(1):198. htps://doi.org/10.1186/1471-2148-8-198

15. Guan C, Xue Y, Jiang P, He C, Zhuge X, Lan T, and Yang H. Overexpression of PtoCYCD3;3 Promotes Growth and Causes Leaf Wrinkle and Branch Appearance in Populus. IJMS. 2021:22(3):1288. htps://doi.org/10.3390/ijms22031288

16. Li L, Sun W, Zhou P, Wei H, Wang P, Li H, Rehman S, Li D, and Zhuge Q. Genome-Wide Characterization of Dirigent Proteins in Populus: Gene Expression Variation and Expression Patern in Response to Marssonina brunnea and Phytohormones. Forests. 2021:12(4):507. htps://doi.org/10.3390/f12040507

17. Showalter AM, Keppler BD, Liu X, Lichtenberg J, and Welch LR. Bioinformatic Identification and Analysis of Hydroxyproline-Rich Glycoproteins in Populus trichocarpa. BMC Plant Biol. 2016:16(1):229. htps://doi.org/10.1186/s12870-016-0912-3

18. Wang H, Leng X, Yang J, Zhang M, Zeng M, Xu X, Wang F, and Li C. Comprehensive analysis of AHL gene family and their expression under drought stress and ABA treatment in Populus trichocarpa. Transcriptome-wide identification and characterization of microRNAs in diverse phases of wood formation in Populus trichocarpa. 2021a:9:e10932. htps://doi.org/10.7717/peerj.10932
