# Supplementary material for: Gene network analysis predicts the primary regulators of ABA-dependent transcriptional activation and repression in Populus roots
Source: PLoS Genet. 2026 Aug 4;22(8):e1012256. doi: 10.1371/journal.pgen.1012256 (PMC13466054; doi:10.1371/journal.pgen.1012256)
Supplement: Supplementary file 13 [file pgen.1012256.s013.pdf]

Cohen *et al.* Gene network analysis predicts the primary regulators of ABA-dependent transcriptional activation and repression in *Populus* roots

A

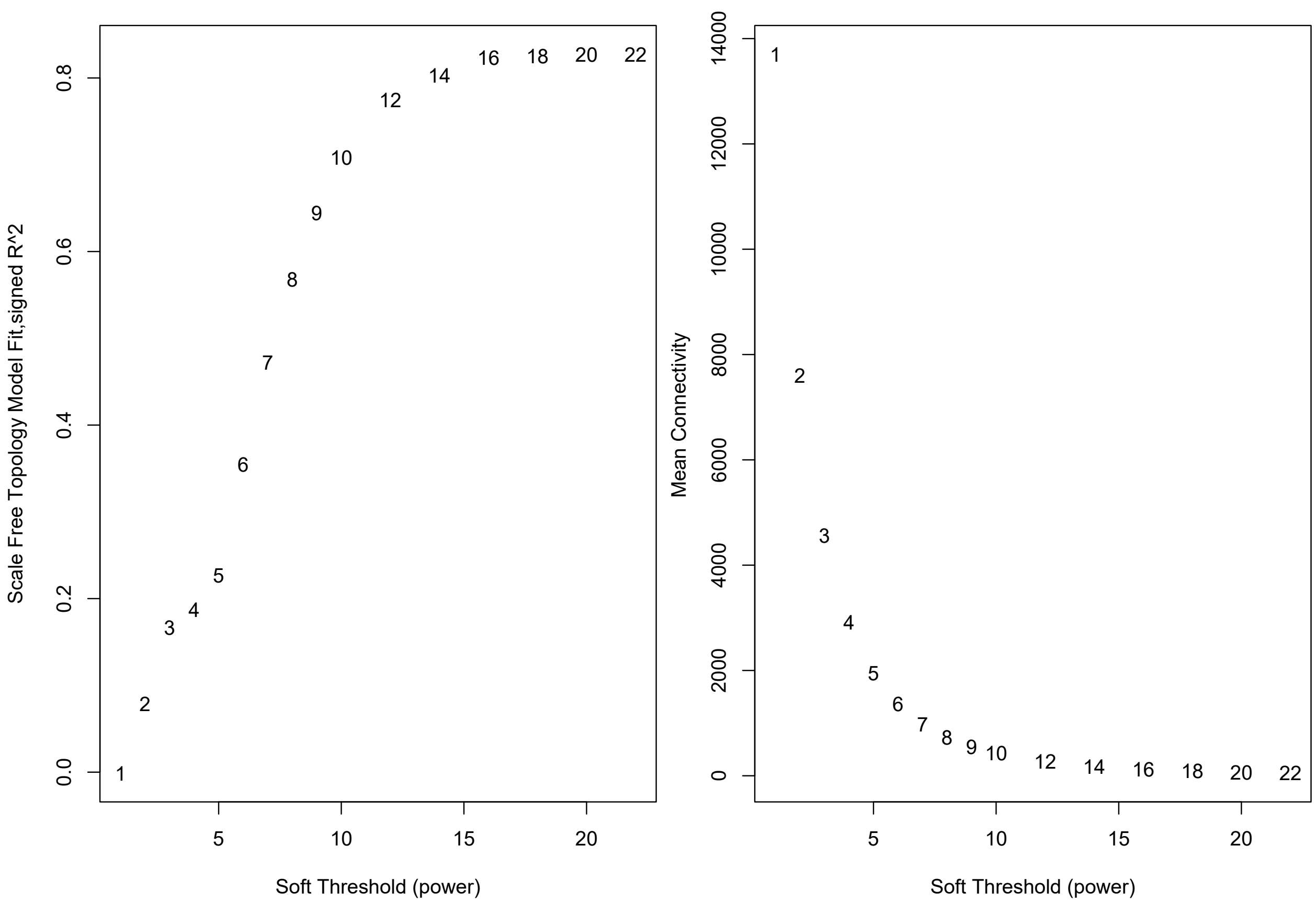

B **Gene dendrogram and module colors**

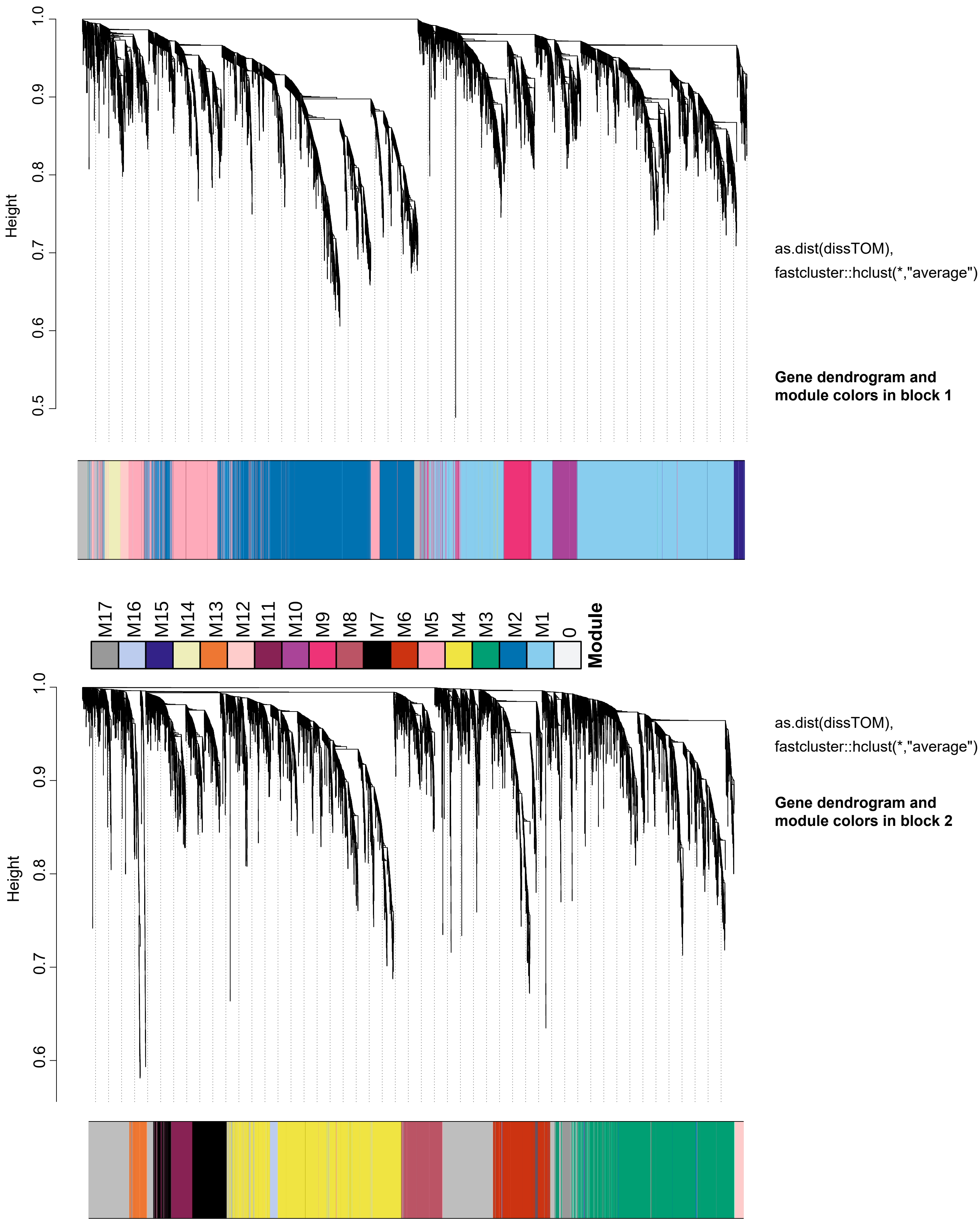

S13 Figure: Co-expression network construction. Scale-free topology was obtained by applying a soft power of 18, which was determined by the network topology analysis function ‘pickSoftThreshold’ (with default parameters, “signed” network type and biweight midcorrelation “bicor”). (A) soft thresold selection (B) Gene dendrogram and module colors.
