# Supplementary material for: Gene network analysis predicts the primary regulators of ABA-dependent transcriptional activation and repression in Populus roots
Source: PLoS Genet. 2026 Aug 4;22(8):e1012256. doi: 10.1371/journal.pgen.1012256 (PMC13466054; doi:10.1371/journal.pgen.1012256)
Supplement: Supplementary file 14 [file pgen.1012256.s022.docx]

**Cohen *et al*. Gene network analysis predicts the primary targets of abscisic acid signal in the poplar root transcriptome**

[**S**](https://oup.silverchair-cdn.com/oup/backfile/Content_public/Journal/plcell/37/10/10.1093_plcell_koaf217/1/koaf217_supplementary_data.zip?Expires=1765930877&Signature=qQC54tDz6R35v5wTR3LVGD28p-JOcMrgYmKKAgPaIqUw5FfgGKJ~piEZMJqpTMr7yI4AtxyXwEI3U3ZqUSKLTeOUopkat7G~ddgnIaGQC9Q00hkDsIfYkDdSTaIdi2VSYRA3CCx0PIYE87t1XMIxkVvyRxG4HRtXmkVrK2LTlwInL3uryNrXOm7bE66y5HauUr1RjlI9ERVNZhyKg97MF6v7pehDzEapPsbBwMmlOp~vfAJZ00mzTWXVG4wet-gJSctr4vrBkFySwQYxYP~vzV4fELWI7S8vC1n4CHiKYjIAEmZWP9ML6KM5j6uC3L6Eg1-EzZ-aNE868ZlOcjux-g__&Key-Pair-Id=APKAIE5G5CRDK6RD3PGA)**1 Protocol:**

A. **cDNA library construction**

48 cDNA libraries were built using the TruSeq Stranded mRNA Sample Prep kit (Illumina, California, USA) according to manufacturer’s instructions. mRNA were purified using poly-(T) beads from 4 µg of each total RNA sample, then cleaved in segments of 155 bp on average (120-210 bp range). Cleaved RNA fragments were then primed with random hexamers and reverse transcribed into first strand cDNA. A second strand of cDNA was consecutively synthetized, and double-stranded cDNA was purified using beads. The 3’ ends of the blunt fragments were then adenylated. Indexed adapters were ligated to the cDNA fragments enriched by PCR (11 cycles). Libraries were purified and quality-assessed using a Fragment Analyzer (Advanced Analytical Technologies, Inc., Iowa, USA). Libraries were quantified by QPCR using the Kapa Library Quantification Kit.

B. **RNA sequencing processing**

Trimming and filtering were done using Erne v2.1.1 [1], read mapping was performed with TopHat2 v2.12 [2,3] and the following parameters : -r 83, --mate-std-dev 21, -g 10, --read-mismatch 15, --read-edit-dist 30, and --read-gap-length 15, and --read-realign-edit-dist 10. Single mapped reads, ~ 98% of mapped reads, were used to construct a reference annotation-based transcript assembly [4]. Assembly was performed using Cufflinks v2.2.1 [5]. A merged.gtf file was created from transcripts.gtf output files using Cuffmerge. Artefactual contigs were removed. The new assembly consisted in 35,956 loci, including 32,154 unique predicted genes, 1,184 chimera and 2,618 putative unannotated transcriptionally active regions (uTAR). Raw read counts were summarized (~ 69 million counts per library) using the ‘featureCounts’ command with the following parameters : -O –s2 -p -P -d20 -D55000 (Subread v1.6.1 [6])

C. **Gene Ontology and Mapman bin**

GO assignment and enrichment analysis were performed using Blast2GO v5.2 [7]. The 34,699 protein sequences encoded by the *P. trichocarpa* v4.1 primary transcripts were blasted against *Arabidopsis thaliana* (‘blastp’, default parameters, except e-value threshold=0.001). Only the best hit was imported, linking poplar gene models to the GO identifiers of their closest Arabidopsis homologs. GO was summarized as plant GO-Slim terms. *P. trichocarpa* v4.1 protein sequences were submitted to the Mercator web application v3.6 using default parameters and were assigned to MapMan Bins [8].

**Reference**

1.	Del Fabbro C, Scalabrin S, Morgante M, Giorgi FM. An Extensive Evaluation of Read Trimming Effects on Illumina NGS Data Analysis. PLoS ONE. 2013;8: e85024. doi:10.1371/journal.pone.0085024

2.	Trapnell C, Pachter L, Salzberg SL. TopHat: discovering splice junctions with RNA-Seq. Bioinformatics. 2009;25: 1105–1111. doi:10.1093/bioinformatics/btp120

3.	Kim D, Pertea G, Trapnell C, Pimentel H, Kelley R, Salzberg SL. TopHat2: accurate alignment of transcriptomes in the presence of insertions, deletions and gene fusions. Genome Biol. 2013;14: R36. doi:10.1186/gb-2013-14-4-r36

4.	Roberts A, Pimentel H, Trapnell C, Pachter L. Identification of novel transcripts in annotated genomes using RNA-Seq. Bioinformatics. 2011;27: 2325–2329. doi:10.1093/bioinformatics/btr355

5.	Trapnell C, Williams BA, Pertea G, Mortazavi A, Kwan G, Van Baren MJ, et al. Transcript assembly and quantification by RNA-Seq reveals unannotated transcripts and isoform switching during cell differentiation. Nat Biotechnol. 2010;28: 511–515. doi:10.1038/nbt.1621

6.	Liao Y, Smyth GK, Shi W. The Subread aligner: fast, accurate and scalable read mapping by seed-and-vote. Nucleic Acids Res. 2013;41: e108–e108. doi:10.1093/nar/gkt214

7.	Conesa A, Götz S, García-Gómez JM, Terol J, Talón M, Robles M. Blast2GO: a universal tool for annotation, visualization and analysis in functional genomics research. Bioinformatics. 2005;21: 3674–3676. doi:10.1093/bioinformatics/bti610

8.	Lohse M, Nagel A, Herter T, May P, Schroda M, Zrenner R, et al. Mercator: a fast and simple web server for genome scale functional annotation of plant sequence data: Mercator: sequence functional annotation server. Plant Cell Environ. 2014;37: 1250–1258. doi:10.1111/pce.12231
