# Supplementary material for: Gene network analysis predicts the primary regulators of ABA-dependent transcriptional activation and repression in Populus roots
Source: PLoS Genet. 2026 Aug 4;22(8):e1012256. doi: 10.1371/journal.pgen.1012256 (PMC13466054; doi:10.1371/journal.pgen.1012256)
Supplement: Supplementary file 15 [file pgen.1012256.s023.docx]

**Cohen *et al*. Gene network analysis predicts the primary regulators of ABA-dependent transcriptional activation and repression in *Populus* roots**

**S1 Appendix:** **Transporters and channels**

ABA remodelled the *transporter* landscape in the poplar root transcriptome (S7 Fig). Among WGCNA modules, the ABA-inactivated M3 was enriched in “transport” with 206 annotated unigenes (Fig 3C, S4 and S5 Data). Notably, M3 was enriched in “nitrate transport” (S7 Fig, S5 Data). Putative nitrate transporters were present in the ABA-inactivated M3, including 18 members of the *PtNPF* family*,* six members of *PtNRT2* and *PtNRT3s* families, and the poplar homolog of *ATNITR2;1*, a putative nitrite transporter [1]. In Arabidopsis, ABA transport is mediated by NFPs, ATP-binding cassette (ABCG) transporters, multidrug and toxic compound extrusion (MATE)-type/DTX transporters [2–7]. In rice, the AWPM-19 family protein, OsPM1, has also been shown to mediate ABA influx [8]. Here, we showed that putative ABA transporters were transcriptionally regulated by ABA in roots (S7 Fig). The ABA-inactivated M3 included some *ABCG40* homologs*, PtNPF4.7, PtNPF4.8, PtrDTX49*, and *PtrDTX59* while the ABA-activated M4 included two *ABCG25* homologs, an *AWPM-19-like* gene*, PtNPF4.4* and the hub gene *PtNPF4.3*.

On the other hand, our work showed the dual impact of ABA on the expression of the *aquaporin* family. Both the ABA-activated M4 and the ABA-inactivated M3 were enriched in “major intrinsic protein”, highlighting the differential regulation of *aquaporins* (S5 Data, S7C Fig). AQUAPORINs are multifunctional transmembrane proteins that facilitate the movement of water and solutes, and are regulated by ABA at transcriptional and post-transcriptional levels [9–14]. The *aquaporins* associated with the ABA-activated M4 were *tonoplastic intrinsic protein*s (*PtTIP1s* and *PtTIP2s*) and *plasma membrane intrinsic proteins* (*PtPIP1s* and *PtPIP2s*), including the hub *PtPIP2;7*, a homolog of *RD28* [15]. TIPs and PIPs are involved in water movement [12]. Twelve *aquaporins* present in the ABA-activated M4 also show up-regulated expression in poplar roots submitted to soil water deficit [16]. Consistently, five M4 *aquaporins* were predicted targets of ABF/ABI5. Conversely, the *aquaporins* associated with the ABA-inactivated M3 were *unrecognized X intrinsic proteins (PtXIPs)* and *nodulin-26 intrinsic proteins* (*PtNIPs*), including the hub *PtNIP2;1*. The expression of *OsLsi1,* the rice ortholog of *PtNIP2;1,* is down-regulated in ABA-treated roots [17]. Both *PtNIP2;1* and *OsLsi1* are drought responsive [17,18]. PtNIP2;1 was functionally confirmed as a poplar silicon influx transporter [19,20].

ABA potentially modulates osmosensing as suggested by the transcriptional regulation of the *OSCA* family in M3 and M4 (S7D Fig). OSCAs are mechanically-activated ion channels, with 17 members in poplars [21], known as hyperosmolality-gated calcium-permeable channel with functions in osmosensing [22]. Notably, the ABA-activated M4 included highly connected OSCA encoding genes (*PtOSCA3.1*, *PtOSCA2.2* and *PtOSCA1.7,* S7D Fig) with the M4 hub *PtOSCA3.1* being orthologous to *ERD4*. In Arabidopsis, RD and ERD genes encode several families of core drought-inducible proteins and are widely used as transcriptional drought reporters [9,23].

Our findings highlighted that ABA antagonized the anthocyanin/flavonoid pathway decreasing the expression of genes involved in the metabolism, and the regulation of transcription, but also in the transport (S7E and S9 Figs). The ABA-inactivated M3 highlighted the ABA-dependent remodelling of anthocyanin/flavonoid transport (S7E Fig): *PtrDTX20*, *PtrDTX19* and *PtrDTX3* encode three MATE transporters homologous to TRANSPARENT TESTA 12 (TT12) while *Pt-AHA10.1* and *Pt-ATGSTF10.1* are *TT13* and *TT19* orthologs, respectively. TT12 is a tonoplast-located proton antiporter and mediates transport of selected flavonoids such as cyanidin-3-O-glucoside into the vacuole [24]. TT13 is a tonoplastic autoinhibited H+-ATPase that generates, *via* a proton gradient, the driving force for TT12-mediated transport of proanthocyanidin precursors to the vacuole [25]. TT19 is a glutathione transferase that has been suggested to mediate vacuolar transport of anthocyanins [26,27].

**references**

1.	Maeda S, Konishi M, Yanagisawa S, Omata T. Nitrite Transport Activity of a Novel HPP Family Protein Conserved in Cyanobacteria and Chloroplasts. Plant Cell Physiol. 2014;55: 1311–1324. doi:10.1093/pcp/pcu075

2.	Boursiac Y, Léran S, Corratgé-Faillie C, Gojon A, Krouk G, Lacombe B. ABA transport and transporters. Trends Plant Sci. 2013;18: 325–333. doi:10.1016/j.tplants.2013.01.007

3.	Zhang H, Zhu H, Pan Y, Yu Y, Luan S, Li L. A DTX/MATE-type transporter facilitates abscisic acid efflux and modulates ABA sensitivity and drought tolerance in arabidopsis. Mol Plant. 2014;7: 1522–1532. doi:10.1093/mp/ssu063

4.	Kuromori T, Shinozaki K. ABA transport factors found in Arabidopsis ABC transporters. Plant Signal Behav. 2010;5: 1124–1126. doi:10.4161/psb.5.9.12566

5.	Kanno Y, Hanada A, Chiba Y, Ichikawa T, Nakazawa M, Matsui M, et al. Identification of an abscisic acid transporter by functional screening using the receptor complex as a sensor. Proc Natl Acad Sci. 2012;109: 9653–9658. doi:10.1073/pnas.1203567109

6.	Kang J, Yim S, Choi H, Kim A, Lee KP, Lopez-Molina L, et al. Abscisic acid transporters cooperate to control seed germination. Nat Commun. 2015;6: 8113. doi:10.1038/ncomms9113

7.	Léran S, Noguero M, Corratgé-Faillie C, Boursiac Y, Brachet C, Lacombe B. Functional characterization of the arabidopsis abscisic acid transporters NPF4.5 and NPF4.6 in xenopus oocytes. Front Plant Sci. 2020;11: 144. doi:10.3389/fpls.2020.00144

8.	Yao L, Cheng X, Gu Z, Huang W, Li S, Wang L, et al. The AWPM-19 family protein OsPM1 mediates abscisic acid influx and drought response in rice. Plant Cell. 2018;30: 1258–1276. doi:10.1105/tpc.17.00770

9.	Taji T, Seki M, Yamaguchi-Shinozaki K, Kamada H, Giraudat J, Shinozaki K. Mapping of 25 drought-inducible genes, RD and ERD, in arabidopsis thaliana. Plant Cell Physiol. 1999;40: 119–123. doi:10.1093/oxfordjournals.pcp.a029469

10.	Jang JY, Kim DG, Kim YO, Kim JS, Kang H. An Expression Analysis of a Gene Family Encoding Plasma Membrane Aquaporins in Response to Abiotic Stresses in Arabidopsis Thaliana. Plant Mol Biol. 2004;54: 713–725. doi:10.1023/B:PLAN.0000040900.61345.a6

11.	Chaumont F, Moshelion M, Daniels MJ. Regulation of plant aquaporin activity. Biol Cell. 2005;97: 749–764. doi:10.1042/BC20040133

12.	Maurel C, Verdoucq L, Luu D-T, Santoni V. Plant Aquaporins: Membrane Channels with Multiple Integrated Functions. Annu Rev Plant Biol. 2008;59: 595–624. doi:10.1146/annurev.arplant.59.032607.092734

13.	Gomes D, Agasse A, Thiébaud P, Delrot S, Gerós H, Chaumont F. Aquaporins are multifunctional water and solute transporters highly divergent in living organisms. Biochim Biophys Acta BBA - Biomembr. 2009;1788: 1213–1228. doi:10.1016/j.bbamem.2009.03.009

14.	Parent B, Hachez C, Redondo E, Simonneau T, Chaumont F, Tardieu F. Drought and Abscisic Acid Effects on Aquaporin Content Translate into Changes in Hydraulic Conductivity and Leaf Growth Rate: A Trans-Scale Approach. Plant Physiol. 2009;149: 2000–2012. doi:10.1104/pp.108.130682

15.	Yamaguchi-Shinozaki K, Koizumi M, Urao S, Shinozaki K. Molecular Cloning and Characterization of 9 cDNAs for Genes That Are Responsive to Desiccation in Arabidopsis thaliana: SequenceAnalysis of One cDNA Clone That Encodes a Putative Transmembrane Channel Protein. Plant Cell Physiol. 1992;33: 217–224. doi:10.1093/oxfordjournals.pcp.a078243

16.	Cohen D, Bogeat-Triboulot M-B, Vialet-Chabrand S, Merret R, Courty P-E, Moretti S, et al. Developmental and Environmental Regulation of Aquaporin Gene Expression across Populus Species: Divergence or Redundancy? PLoS ONE. 2013;8: e55506. doi:10.1371/journal.pone.0055506

17.	Yamaji N, Ma JF. Spatial Distribution and Temporal Variation of the Rice Silicon Transporter Lsi1. Plant Physiol. 2007;143: 1306–1313. doi:10.1104/pp.106.093005

18.	Cohen D, Bogeat-Triboulot M-B, Tisserant E, Balzergue S, Martin-Magniette M-L, Lelandais G, et al. Comparative transcriptomics of drought responses in Populus: a meta-analysis of genome-wide expression profiling in mature leaves and root apices across two genotypes. BMC Genomics. 2010;11: 630. doi:10.1186/1471-2164-11-630

19.	Deshmukh RK, Vivancos J, Ramakrishnan G, Guérin V, Carpentier G, Sonah H, et al. A precise spacing between the NPA domains of aquaporins is essential for silicon permeability in plants. Plant J. 2015;83: 489–500. doi:10.1111/tpj.12904

20.	Hassan MM, Martin S, Feng K, Yates TB, Yuan G, Martin MZ, et al. Genome-wide identification and functional prediction of silicon (Si) transporters in poplar (Populus trichocarpa). Plant Biotechnol Rep. 2023;17: 285–302. doi:10.1007/s11816-022-00788-4

21.	Hartmann FP, Tinturier E, Julien J-L, Leblanc-Fournier N. Between Stress and Response: Function and Localization of Mechanosensitive Ca2+ Channels in Herbaceous and Perennial Plants. Int J Mol Sci. 2021;22: 11043. doi:10.3390/ijms222011043

22.	Yuan F, Yang H, Xue Y, Kong D, Ye R, Li C, et al. OSCA1 mediates osmotic-stress-evoked Ca2+ increases vital for osmosensing in Arabidopsis. Nature. 2014;514: 367–371. doi:10.1038/nature13593

23.	Wu G, Tian N, She F, Cao A, Wu W, Zheng S, et al. Characteristics analysis of *Early Responsive to Dehydration* genes in Arabidopsis thaliana ( *AtERD* ). Plant Signal Behav. 2022; 2105021. doi:10.1080/15592324.2022.2105021

24.	Marinova K, Pourcel L, Weder B, Schwarz M, Barron D, Routaboul J-M, et al. The Arabidopsis MATE transporter TT12 acts as a vacuolar flavonoid/H+ -antiporter active in proanthocyanidin-accumulating cells of the seed coat. Plant Cell. 2007;19: 2023–2038. doi:10.1105/tpc.106.046029

25.	Appelhagen I, Nordholt N, Seidel T, Spelt K, Koes R, Quattrochio F, et al. TRANSPARENT TESTA 13 is a tonoplast P _3A_ -ATPase required for vacuolar deposition of proanthocyanidins in *Arabidopsis thaliana* seeds. Plant J. 2015;82: 840–849. doi:10.1111/tpj.12854

26.	Kitamura S, Shikazono N, Tanaka A. *TRANSPARENT TESTA 19* is involved in the accumulation of both anthocyanins and proanthocyanidins in *Arabidopsis*. Plant J. 2004;37: 104–114. doi:10.1046/j.1365-313X.2003.01943.x

27.	Sun Y, Li H, Huang J-R. Arabidopsis TT19 Functions as a Carrier to Transport Anthocyanin from the Cytosol to Tonoplasts. Mol Plant. 2012;5: 387–400. doi:10.1093/mp/ssr110
