# Supplementary material for: Gene network analysis predicts the primary regulators of ABA-dependent transcriptional activation and repression in Populus roots
Source: PLoS Genet. 2026 Aug 4;22(8):e1012256. doi: 10.1371/journal.pgen.1012256 (PMC13466054; doi:10.1371/journal.pgen.1012256)
Supplement: Supplementary file 16 [file pgen.1012256.s024.docx]

**Cohen *et al.* Gene network analysis predicts the primary regulators of ABA-dependent transcriptional activation and repression in *Populus* roots**

**S2 Appendix:** **Genes mentioned in the main text and in S1 Appendix are as follows:**

ABCG25 (AT1G71960); ABCG30 (AT4G15230); ABCG31 (AT2G29940); ABCG40 (AT1G15520); ABF: ABF1 (AT1G49720), ABF2 (AT1G45249), ABF3 (AT4G34000), ABF4 (AT3G19290); ABI5 (AT2G36270); ABH2 (AT4G27250); ABT: AIW1 (AT3G18950), AIW2 (AT1G49450); AFP: AFP1 (AT1G69260), AFP2 (AT1G13740), AFP3 (AT3G29575), AFP4 (AT3G02140); AIRP2 (AT5G01520); AITR: AITR1 (AT3G27250), AITR2 (AT3G48510), AITR3 (AT5G40790), AITR4 (AT5G40800), AITR5 (AT5G50360), AITR6 (AT5G63350); ANAC016 (AT1G34180); DTX50 (AT5G52050); ATHB12 (AT3G61890); ATHB7 (AT2G46680); ATNITR2;1 (AT5G62720); ATOR-LIKE (AT5G06130); ATPUB22 (AT3G52450); ATPUB23 (AT2G35930); BCM: BCM1 (AT2G35260), BCM2 (AT4G17840); bZIP16 (AT2G35530); bZIP63 (AT5G28770); bZIP68 (AT1G32150); CHYR1 (AT5G22920); CIA2 (AT5G57180); CIL (AT4G25990); CRL1 (AT2G33590); DAA1 (AT1G64110); EGR: EGR1 (AT3G05640), EGR2 (AT5G27930), EGR3 (AT3G16800); ELA: ELA1 (AT5G24910), ELA2 (AT5G24900); ERD7 (AT2G17840); GBF1 (AT4G36730); GBF3 (AT2G46270); GLK: GLK1 (AT2G20570), GLK2 (AT5G44190); GT3: GT3a (AT5G01380), GT3b (AT2G38250); GUN4 (AT3G59400); HEMD (AT2G26540); LDAPs: LDAP1 (AT1G67360), LDAP2 (AT2G47780), LDAP3 (AT3G05500); MYB41 (AT4G28110); MYB49 (AT5G54230); MYB71 (AT3G24310); MYB74 (AT4G05100); MYB79 (AT4G13480); MYB102 (AT4G21440); MYB121 (AT3G30210); MYC2 (AT1G32640); NCED3 (AT3G14440); NDUFS4 (AT5G67590); NeoPAR1 (AT1G09510); NPF4.1 (AT3G25260); NPF4.2 (AT3G25280); NPF4.3 (AT1G59740); NPF4.4 (AT1G33440); NPF4.5 (AT1G27040); NPF4.6 (AT1G69850); PSY (AT5G17230); RD17 (AT1G20440); RD28 (AT2G37180); RD29B (AT5G52300); RPGE: RPGE2 (AT3G55240), RPGE3 (AT3G28990); SAG113 (AT5G59220); SAG201 (AT2G45210); SCL23 (AT5G41920); SCR (AT3G54220); SCS (AT4G38810); SGR1 (AT4G22920); SHR (AT4G37650); SNAC: ATAF1 (AT1G01720), ANAC032 (AT1G77450), RD26 (AT4G27410), ANAC053 (AT3G10500), ANAC019 (AT1G52890), ATNAP (AT1G69490), ANAC047 (AT3G04070); TSPO (AT2G47770); TT12 (AT3G59030); TT13 (AT1G17260); TT19 (AT5G17220); TTG1 (AT5G24520); XERICO (AT2G04240); AT1G07040 (AT1G07040); AT2G25890 (AT2G25890); bZIP63 (Potri.013G040700); LTF15 (Potri.014G083900); MAX3 (Potri.014G056800); NAC095 (Potri.010G166200); NAC163 (Potri.019G031400); PagAFP2a (Potri.008G096500); PoptrBAN1 (Potri.004G030700); Potri.001G007700 (Potri.001G007700); Potri.001G112600 (Potri.001G112600); Potri.001G143400 (Potri.001G143400); Potri.001G334500 (Potri.001G334500); Potri.002G206100 (Potri.002G206100); Potri.003G097600 (Potri.003G097600); Potri.003G106500 (Potri.003G106500); Potri.004G166900 (Potri.004G166900); Potri.006G075200 (Potri.006G075200); Potri.006G099600 (Potri.006G099600); Potri.006G121801 (Potri.006G121801); Potri.006G209000 (Potri.006G209000); Potri.006G210500 (Potri.006G210500); Potri.007G029400 (Potri.007G029400); Potri.007G136901 (Potri.007G136901); Potri.008G194800 (Potri.008G194800); Potri.009G109500 (Potri.009G109500); Potri.010G211500 (Potri.010G211500); Potri.012G094400 (Potri.012G094400); Potri.013G012200 (Potri.013G012200); Potri.014G034700 (Potri.014G034700); Potri.015G091900 (Potri.015G091900); Potri.015G002600 (Potri.015G002600); Potri.015G065500 (Potri.015G065500); Potri.017G015800 (Potri.017G015800); Potri.017G030100 (Potri.017G030100); Potri.017G138900 (Potri.017G138900); Potri.018G142100 (Potri.018G142100); Potri.019G064200 (Potri.019G064200); PtaGA2ox1 (Potri.001G378400); PtaGA2ox6 (Potri.011G095600); Pt-AHA10.1 (Potri.001G161400); PtAREB7 (Potri.008G010800); PtATAF1.1 (Potri.005G180200); PtATAF1.2 (Potri.002G081000); Pt-ATGSTF10.1 (Potri.017G138800); Pt-ATHB.3 (Potri.001G083700); Pt-ATHB.5 (Potri.014G103000); PtbHLH131 (Potri.005G208600); PtbHLH175 (Potri.003G092200); PtbHLH178 (Potri.001G142200); PtCHYR1 (Potri.009G005700); PtCHYR2 (Potri.006G245400); PtCRE1a (Potri.008G137900); Pt−CYP735.1 (Potri.017G114200); Pt−CYP735.2 (Potri.004G100400); PtD27c (Potri.002G046500); PtGA3ox1 (Potri.001G176600); PtGRAS39 (Potri.006G021400); PtHsp100-01 (Potri.001G097600); PtIPT5a (Potri.008G202200); PtLDAP1b (Potri.003G173100); PtNAC049 (Potri.005G069500); PtNAC056 (Potri.005G200100); PtNAC078 (Potri.007G099400); PtNAC105 (Potri.011G123300); PtNAC118 (Potri.013G054000); PtNCED3 (Potri.011G112400); Pt-NCED3.1 (Potri.001G393800); PtNF-YA10 (Potri.006G053500); Pt-GUN4.2 (Potri.007G128200); PtNIP2;1 (Potri.017G083300); PtNPF1.6 (Potri.018G041700); PtNPF4.3 (Potri.003G000800); PtNPF4.4 (Potri.019G067500), PtNPF4.7 (Potri.014G036200); PtNPF4.8 (Potri.002G129500), PtNPF5.19 (Potri.013G106925); PtNPF6.5 (Potri.002G225500); PtNRT3.1A (Potri.012G089300), PtNRT3.2A (Potri.012G089400); PtOLE5 (Potri.012G059400); PtOLE6 (Potri.006G234900); PtOSCA1.7 (Potri.014G156100); PtOSCA2.2 (Potri.002G113800); PtOSCA3.1 (Potri.001G358300); PtPIP2;7 (Potri.016G089500); PtrABF3 (Potri.004G140600); PtrABI5 (Potri.006G083000); PtrAREB1-2 (Potri.002G125400); PtrAREB1-3 (Potri.009G101200); PtrAREB1-4 (Potri.014G028200); PtrbHLH28 (Potri.002G114700); PtrbHLH40 (Potri.002G252800); PtrbHLH60 (Potri.005G001800); PtrbHLH117 (Potri.010G040000); PtrbHLH124 (Potri.010G137600); PtrbHLH165 (Potri.015G048000); PtRD26 (Potri.001G404100); PtrDTX3 (Potri.002G055100); PtrDTX19 (Potri.005G207500); PtrDTX20 (Potri.005G207600); PtrDTX49 (Potri.012G133500); PtrDTX59 (Potri.015G135600); PtrGBF3 (Potri.002G167100); PtrGT29 (Potri.006G101400); PtrHAB14 (Potri.009G037300); PtrLEA55 (Potri.009G120100); PtrMAX1b (Potri.018G062100); PtrMAX4b (Potri.006G238500); PtrMYB006 (Potri.001G005100); PtrMYB009 (Potri.001G086700); PtrMYB014 (Potri.004G115600); PtrMYB015 (Potri.004G118000); PtrMYB043 (Potri.011G041600); PtrMYB115 (Potri.002G173900); PtrMYB123 (Potri.003G144200) PtrMYB134 (Potri.006G221800); PtrMYB139 (Potri.008G180800); PtrMYB153 (Potri.003G144300); PtrMYB165 (Potri.010G114000); PtrMYB179 (Potri.015G022000); PtrMYB194 (Potri.008G128500); PtrMYB201 (Potri.014G100800); PtrMYB203 (Potri.015G041100); PtrMYB214 (Potri.008G173400); PtrPUB72 (Potri.012G078900); PtrPUB82 (Potri.015G074200); PtrWRKY77 (Potri.003G182200); PtSAUR87 (Potri.014G066900); PtSGR1 (Potri.003G119600); PtSCR (Potri.006G114200); PtSHR1 (Potri.007G063300); PtSHR2B (Potri.017G019900); PtWD40 (Potri.016G075800); PtXERICO (Potri.014G170400); RD29b (Potri.012G141300); DD15 (glyma.02g021600); OsLsi1 (Os02g0745100); OsPM1(Os05g0381400); FLO7 (Os10g0463800).
