# Supplementary material for: Gene network analysis predicts the primary regulators of ABA-dependent transcriptional activation and repression in Populus roots
Source: PLoS Genet. 2026 Aug 4;22(8):e1012256. doi: 10.1371/journal.pgen.1012256 (PMC13466054; doi:10.1371/journal.pgen.1012256)
Supplement: Supplementary file 17 [file pgen.1012256.s026.docx]

Dear Editors and Reviewers,

Please find enclosed the revised version of our manuscript. The authors sincerely thank the reviewers for their constructive comments and suggestions, which have helped improve the quality and clarity of the manuscript. We have carefully considered all remarks and revised the manuscript accordingly where appropriate. Detailed responses to each comment are provided below.

Sincerely,

The authors.

**Comments to the Authors**

**Reviewer #1**: This manuscript assayed the effect of extraneous ABA on non-stressed plant roots as a method to estimate the regulatory cascade in response to ABA without the confounding effects of other stress sensors. The authors posit that this work fills an important knowledge gap in understanding root responses to ABA, particularly in Poplar. The study was well-designed and the authors have done meticulous work in analyzing the transcriptomic data to identify the individual genes and broad trends involved in ABA response. I particularly appreciate that they compared the ABA response in this study to the osmotic stress response which helps place the pure ABA-triggered response within the broader osmotic stress faced often by plants. However, there are a few shortcomings in the way the paper is conceived and presented.

We thank Reviewer #1 for the careful evaluation of our manuscript and for the positive assessment of the experimental approach, the study design and the transcriptomic analyses.

1. The authors correctly identify that there is a knowledge gap in our understanding of ABA response in roots. However, beyond the transcriptome data and the derived functional analyses the authors do not address the key question of what knowledge was gained by the design and execution of this experiment. An important component of plant response to drought stress is the change to the root system architecture, which is presumably within the purview of ABA signaling. This work would benefit from a systematic comparison of the ABA response seen here to the previously described studies on root development to define the effect of ABA responsive modules on root traits such as meristem activity, lateral root initiation and expansion etc.

We thank Reviewer #1 for this insightful comment and for the opportunity to clarify the biological insight gained from this study. Our primary objectives were to identify putative primary ABA target genes in poplar roots, assess the relative contribution of transcriptional activation and repression, and characterize how these responses are coordinated over time (Introduction, Lines 150–153). Within this framework, our study reveals the temporal dynamics of ABA-dependent transcriptional responses in roots and provides a structured view of ABA-dependent transcriptional regulation under controlled, non-stressed conditions. The primary ABA response in roots involves molecular functions and regulatory processes previously characterized in diverse biological contexts and tissues. A key finding of this study is that ABA signalling in roots rapidly engages coordinated regulatory pathways and functional responses previously associated with key processes such as germination, stress responses, metabolic regulation, and leaf senescence. We have further emphasized these aspects in the revised Discussion to better highlight the biological significance and the novelty of the study (Lines 611-613 and 708-729).

We understand the reviewer’s concern as a request to address the downstream developmental or architectural outcomes of the transcriptional regulations identified here. Yet, our experiment dealt with short-term treatments and young root axes. These one-week-old adventitious roots did not yet harbour lateral roots. A comprehensive evaluation of RSA-related processes would require dedicated analyses and longer-term experiments, thus we have not further emphasized these points in the revised version of the manuscript.

However, we agree that ABA has been extensively implicated in developmental processes such as meristem activity and lateral root formation. To address the reviewer’s suggestion, we performed a systematic comparison between our dataset and genes previously associated with root system architecture (RSA). Using the 767 Arabidopsis genes reported by Justamante et al. (2025) to modulate RSA under abiotic stress conditions, we identified 1,088 poplar homologs corresponding to 572 Arabidopsis genes expressed in our dataset. Yet, this cross-comparison showed that RSA-related genes are distributed across all WGCNA modules, with the highest representation observed in M11 and M12 (9%), whereas ABA-responsive modules displayed comparatively lower proportions (2–5%). These results indicate that genes previously reported as RSA modulators are broadly distributed across the co-expression network and are not preferentially associated within the key early ABA-responsive modules. Among these RSA-related genes, 41 were identified as intramodular hub genes and half of them belonged to ABA-responsive modules. Among them, the ABA-inactivated M3 hub gene, *PtSHR2B*, was confirmed as a strong candidate potentially associated with root development. As we mentioned in our initial Discussion, the co-repression of *PtSHR2B* and *PtGRAS39* in ABA-treated roots is particularly intriguing, and highlights an attractive target for future research. In addition, 13 RSA-related genes were included in the gene regulatory network, predicted by IDREM (New S1 Fig), including the M4 hub genes *PtrAREB1-2*. These genes are putative candidates for ABA-dependent regulation of RSA response to stress, even if to the best of our knowledge, no direct functional evidence currently supports their action in RSA regulation in poplar. This annotation and reference are now included in revised S3 Dataset.

2. This study was performed on roots after the removal of shoot tissue to hone in specifically on the root ABA response. The question then is what was learnt from this approach beyond what was already known from shoot/whole plant studies? Much of the results and discussion sections describe how the known regulators of ABA response (ABFs etc.) were rediscovered here. While that proves the validity of the approach it does not provide any new insights into the root response. The authors should compare and contrast the root response to shoot responses (e.g., from previous studies) to specify what portion of the root response is broadly true across all organs and which portion is specific to roots. If a set of genes are uniquely ABA responsive in roots, then what are the potential regulators of these genes that are identified by the iDREM or WGCNA approaches.

We would like to clarify the rationale underlying our experimental design. Aerial buds were removed from dormant hardwood cuttings prior to cultivation in hydroponics. Poplar develops an adventitious root system from hardwood cuttings even in the absence of leaves and under complete darkness. This experimental set-up therefore controls for well-known confounding effects in time-resolved transcriptomic studies of roots, namely photoperiod and its impact on shoot-to-root communication. Both factors are known to strongly influence ABA signalling and its downstream transcriptional responses. Altogether, this procedure enables the interpretation of transcriptional regulation primarily as a response to ABA treatment rather than as systemic or light-dependent effects.

We have further clarified this point in the revised Introduction (Lines 148-149), Discussion (Lines 611-613) and Materials and methods (Lines 749-750).

The main question raised by Reviewer #1 concerns what was learned beyond what is already known from shoot and whole-plant ABA studies. We agree that canonical ABA regulators such as ABFs were recovered in our analysis, which is expected given the conservation of the ABA signalling pathway. However, the main advance of our study lies in resolving how these components are temporally organized and integrated into coordinated transcriptional modules in roots. Our systematic analysis of ABA-responsive hub genes extends the classical DEG-based approaches by highlighting the associations between core signalling components and downstream ABA-responsive genes within a topology-based framework. By studying the poplar transcriptome, our study highlighted cross-species conservation of numerous processes and also revealed lineage-specific responses. Overall, we found that transcriptional responses previously considered as context- or tissue-specific can occur simultaneously in a rapid and coordinated manner in roots.

Regarding the comparison with shoot responses, the present study was not designed to systematically define root-specific ABA responses. During our previous work, we have explored the root- or shoot-preferred expression pattern in poplars using meta-analysis (Cohen et al. 2010, 2013). We would like to clarify that an unequivocal demonstration of root-specific expression would require the analysis of multiple expression datasets to control for the confounding effects of species, genotype, developmental stage, environmental condition and tissue sampling. Currently, unbiased cross-comparisons with shoots are limited by the lack of datasets generated in the same poplar genotype, under comparable ABA treatments and equivalent temporal resolution across organs. Furthermore, cross-comparisons with Arabidopsis shoot datasets are not straightforward, due to genome expansion in poplar and the resulting ortholog/paralog relationships.

3. The results section includes multiple subsections that discuss the individual classes of genes or processes in ABA response. It is a bit tedious to discuss the effect of ABA on each process and component gene separately. The manuscript would benefit from a discussion of the significance of these changes. For example, what is the significance of repressing Tetrapyrrole and chlorophyll metabolism in roots?

We thank Reviewer #1 for this important comment regarding the biological significance of the ABA-induced transcriptional changes. In the original version of the manuscript, our Discussion primarily focused on the coordination of ABA-responsive molecular processes and on hypotheses concerning the regulatory architecture underlying these responses. We intentionally limited our interpretation to the transcriptional level in order to avoid overstatement of physiological consequences. Accordingly, primary ABA transcriptional responses and their putative impact on molecular processes were summarized and illustrated in a working model (Fig 7). In the revised manuscript, we expanded the Discussion to better address the significance of repressing tetrapyrrole and chlorophyll metabolism in roots and discussed the significance of the transcriptional changes (L708-729).

We would also like to clarify that the Results section was not intended as a catalogue of ABA-responsive genes. Rather, it integrates (i) hub genes identified through network analyses and (ii) previously reported functional evidence from the literature to infer the most likely ABA-responsive molecular processes operating within the M3 and M4 modules. Given the large number of genes identified in this study, we consider this level of detail essential for providing a functionally grounded interpretation of the coordinated transcriptional responses. We have further clarified this point in the revised Results (Lines 376-378) and revised the text to improve readability.

Further specific comments:

1. The design of the study with experiment1 and experiment 2 is difficult to track, especially since there is no direct statement or illustration of the time points sampled. Please include in the first section discussing transcriptomic data (Lines 200-210), an explicit definition of the time points sampled. The embedded timepoint information in Fig. 3B is both too late and too difficult to decipher for the reader.

To improve clarity, we added a schematic illustration of the experimental design and sampling strategy (new Fig 2A) to which we now refer in the text.

2. In lines 205-210 the authors place the point of path divergence as the earliest point before the divergence as visualized in Fig 2. It would be fairer to say that the paths diverged sometime before the next timepoint. For example, line 205 should state “Path A and B separated within 15 minutes of the start of treatment….” Again on line 209 “divergence between path D and F occurred later (between 15 and 30 min), …”

We modified the text accordingly, clarifying that paths diverged within defined time intervals between the split node and the subsequent sampling time point on the iDREM regulatory map (Fig 2C).

3. On lines 240 and 241, the exact sampling timepoints for ABA+ and ABA+/- should be defined. Another suggestion for clarity it to relabel the ABA+/- samples as +60 minutes and +960 minutes or some other way to differentiate them from the 60 and 960 minutes sampled in the ABA+ treatment.

To improve clarity, we now refer to the new schematic illustration (new Fig 2A).

4. Fig. 2C uses the red-white gradient to denote two different contexts, once for module identity and second time for significance which makes it hard to interpret this panel. Please switch to an alternate color gradient for the -log10(p-val) such as green or purple gradient.

To avoid ambiguity, we changed the colour gradient used for the –log10(p-value) representation to a purple gradient in the revised Fig 2 and Fig 3.

5. Fig. 3C preserves the module number for arranging columns, which is not biologically meaningful. The panel would be much easier to interpret is the columns are grouped by the induction/repressed axis used in panel B (i.e., M1,10,4,16 followed by M2,5,3)

We modified the Fig 3C accordingly.

**Reviewer #2:** This paper reports a detailed time‑course analysis of the transcriptomic response that occurs in poplar root tips upon ABA treatment. Using iDREM, the authors classified the early ABA response into six groups, and through WGCNA they identified 17 modules (M1–M17). The discussion focuses on the M3 and M4 modules: M4 forms a large‑scale network centered on ABF/ABI5, which are directly regulated by the ABA core signaling pathway. The ABA‑activated network represents the core of the drought‑stress response, whereas the ABA‑repressed module (M3) integratively suppresses secondary metabolism, senescence‑related pathways, and strigolactone biosynthesis. As the authors note, the root transcriptome is well suited for dissecting short‑term transcriptional responses to ABA. Although several studies in various plant species have reported time‑course transcriptome analyses of ABA‑induced responses, this work is unique in that it collects appropriately fine‑scaled time points and analyzes in detail how transcriptional networks are constructed from the very earliest primary responses. Conducting ABA treatments in complete darkness may also be considered relatively unique. Overall, the experiments are carefully designed, and the resulting transcriptome data appear to be very clean. Given the large volume of high‑quality transcriptomic data, the authors’ decision to focus the paper specifically on transcriptional regulation is logical and appropriate.

We thank Reviewer #2 for the positive evaluation of our work and for highlighting the importance and relevance of focusing on transcription regulations.

Below are several minor corrections:

The authors need to deposit raw transcriptome data to the public site.

Reads were deposited in the Sequence Read Archive (SRA) under Study Accession number (GSE183780). Accession "GSE183780" is currently private and is scheduled to be released on Oct 28, 2026. We will make the accession publicly available earlier if the manuscript is accepted.

To review this accession, please use the following link and copy paste the token in the box. <https://www.ncbi.nlm.nih.gov/geo/query/acc.cgi?acc=GSE183780>

token stmzymmwnbknnqt

Discussion (line 553): “Abscisic acid” → “ABA”

The text was changed accordingly.

Reference list: The formatting lacks consistency and contains errors.

We thank Reviewer #2 for identifying these formatting issues. We carefully checked and revised the reference list to ensure consistency in formatting and to correct the identified errors.

**Reviewer #3:** In this study, Cohen et al. generate time-resolved, high-resolution RNA-seq datasets from Populus roots following ABA treatment. Using these datasets, the authors identify early regulators of this process, pinpoint a potential core gene set involved in ABA-dependent stress responses, and propose a comprehensive working model for transcriptional ABA action in Populus roots. While this study provides a valuable resource to the research community, the manuscript requires substantial revision and structural improvement before it is suitable for publication.

We thank Reviewer #3 for the careful evaluation of our manuscript and for the constructive comments that helped improve the clarity and structure of the manuscript.

Although the authors have generated a comprehensive co-expression network (Figures 3 and 4) and characterized the dynamics of the ABA response (Figure 2), the current analysis remains overly descriptive. Applying a more quantitative approach would greatly improve the holistic understanding of this physiological response and its major regulators.

We acknowledge the reviewer’s concerns and recognize that our manuscript includes some descriptive sections. Nevertheless, we would like to emphasize that the study is grounded in a quantitative analytical framework. WGCNA-derived metrics, including intramodular connectivity, topological overlap, and eigengene correlation were used to define hub genes and characterize module structure, while module preservation statistics were applied to evaluate robustness across independent datasets. In addition, the biological relevance of WGCNA modules was supported through quantitative functional enrichment and correlation analyses. In addition, iDREM is a widely established quantitative framework for time-resolved transcriptomic analysis. Overall, the manuscript integrates statistical and quantitative data to decipher transcriptional processes occurring in the root tips in response to ABA.

The following analytical and structural issues should be addressed:

Regarding Figure 2, the authors should clarify whether any specific transcription factor (TF) families were overrepresented.

Regarding Figure 2, no TF family was found to be significantly overrepresented after multiple testing correction.

In detail, we used Fisher’s exact test combined with FDR correction for multiple testing (adjusted *P*-value threshold = 0.05) and the plantregmap database as reference. This database provided the total number of poplar genes encoding bHLH (202 genes), bZIP (96 genes), BES1 (14 genes), and Trihelix (59 genes). As no significant enrichment was detected, this result was not included in the manuscript.

Furthermore, it is unclear what proportion of the TFs and potential target genes identified in Figure 2 are included in the co-expression network (Figure 4), and to which modules they belong. Integrating this information into a unified gene regulatory network would make these findings significantly more informative.

We thank Reviewer #3 for this insightful comment regarding the integration of WGCNA and iDREM analysis. In the initial version of the manuscript, WGCNA modules and IDREM paths were integrated in the text and visualized in heatmaps (current Fig 6 and S2-S12 Figs). We agree that a unified gene regulatory network (GRN) representation is valuable.

As the iDREM model is inferred from a subset of the dataset used in the WGCNA, we constructed a unified GRN from the 16 putative TFs predicted to control path transition events (Fig 2). This predicted GRN, derived from iDREM analysis, includes 258 genes, including the 16 key TFs, and 965 predicted TF-target interactions. The GRN is described in the Results (Lines 232-239) and in Materials and methods (Lines 817–822). We provided as a new figure (S1A Fig) and reported network features in the revised S2 Dataset.

To address the reviewer’s concern, we performed enrichment analysis using Fisher’s exact test with FDR correction (FDR < 0.05), confirming significant over-representation of iDREM paths within WGCNA modules (revised S2 Dataset). We also used WGCNA module as node attributes onto the GRN (S1B Fig), which shows that ABA-responsive modules are significantly represented within GRN. These results are described in the revised Results (Lines 263-272 and Lines 293-297). We provided a new figure (S1B Fig) and reported network features in the revised S2 Dataset.

A similar issue occurs in the analysis of the public osmotic stress dataset. The authors generate a second co-expression network and compute the correlation between the osmotic and ABA networks (Figure 5). While these data are interesting, the current text and visualizations make it difficult to determine which modules are the most statistically significant in ABA-mediated osmotic regulation, as well as the direction of regulation for the differentially expressed genes (DEGs). The text should be revised to clearly identify the key TFs and their target genes in this context.

We would like to clarify our analytical approach, as there may have been a misunderstanding. We did not generate a second co-expression network, nor did we compute correlations between an osmotic network and an ABA network. We did not either compute or use stress-responsive DEGs. Rather, we assessed whether the modular properties of the ABA network are preserved in an independent dataset. This is a classical quantitative approach implemented using WGCNA and DGCA.

WGCNA metrics, namely Zsummary and Zmean.Adj, quantify the module robustness and replicability across datasets. These results were visualized on Fig 5B-C as bar charts, which provide a clear representation of highly preserved modules (those with values higher than 10). Regarding DGCA, MeDC metrics and associated statistics quantify the modular average differential connectivity in the two datasets. The result was visualized on Fig 5D as a bar chart (MeDC values closest to 0 indicate limited loss of module connectivity in the osmotic dataset). In addition, DGCA quantifies the preservation of pairwise relationships between intramodular hub genes across datasets. In the ABA dataset, hub genes within the same module are strongly co-expressed, and their expression is significantly and positively correlated (+/). In the osmotic dataset, these relationships may be significantly preserved (DGCA class +/+), disrupted (+/0), or significantly divergent (+/−). These DGCA classes were visualized on Fig 5E as a pie chart per module.

We revised the text to improve readability (Lines 319-360), and also revised one sentence in the Abstract (Lines 49-52), which may have been misleading.

Additionally, we thank Reviewer #3 for raising an important question regarding the differential expression of ABA-responsive genes in the context of osmotic stress. In the initial manuscript, DEGs were computed primarily to reconstruct the temporal dynamics of early transcriptional events triggered by ABA (iDREM analysis). To address reviewer’s concern, we identified osmotic-stress related DEGs, defined as genes differentially expressed in response to PEG treatment at least at one time point compared to control (absolute log2 fold-change ≥ 1 and adjusted P-value ≤ 0.01, Wald test with FDR correction). DEG lists are now provided as a resource for future investigations in revised S3 Dataset.

The stress-responsive genes were also mapped onto the iDREM-derived GRN, with node colour indicating the direction of regulation (new S1C Fig). This third GRN is described in the Results (Lines 361-367). We provided a new figure (S1C Fig) and reported network features in the revised S2 Dataset.

The Results subsection titled "Identifying the hallmarks of ABA-dependent transcriptional network" discusses the functions of numerous genes that are either activated or repressed by ABA, relying heavily on heatmaps (Figure 6 and supporting figures). If these genes were derived from the broader network, displaying them as targeted sub-networks would be much more effective. Additionally, this section lacks quantitative context (e.g., noting what percentage of genes involved in ABA feedback loops were actually recovered in the analysis). As currently written, this section reads like a Discussion; it is excessively lengthy and requires substantial reorganization and streamlining.

Regarding the presentation and organization of the section entitled “Identifying the hallmarks of ABA-dependent transcriptional network”, we agree that the large amount of information presented in this section may affect readability.

The objective of this section was not to provide an exhaustive catalogue of ABA-responsive genes, but rather to integrate iDREM- and WGCNA-derived information with previously reported functional evidence in order to identify the molecular processes associated with ABA-responsive modules. Accordingly, the section was intentionally structured into eight subsections, each providing literature-supported evidence linking ABA-responsive hub genes to specific molecular processes or pathways. We have further clarified this point in the revised Results (Lines 376-378) and revised the text to improve readability.

Concerning the quantitative context, most subsections were delineated based on significant GO and Bin term enrichments, whereas others were supported by literature-based inference. We therefore revised the text to better emphasize the quantitative dimension of the analyses when appropriate.

Regarding visualization, we acknowledge the reviewer’s suggestion to display targeted subnetworks. However, we considered heatmaps to provide a more accessible and synthetic representation, particularly because a consistent layout was maintained across all heatmaps. In our view, subnetworks become difficult to interpret when large numbers of genes and interactions are displayed simultaneously, whereas heatmaps allow straightforward visualization of module assignation and associated information while preserving the readability of gene-centred information. This last standing as a major point as our study was dedicated to the identification of the primary targets of ABA signals.

Finally, although we streamlined some paragraphs and revised the text to improve readability and reduce redundancy, we did not substantially reorganize this section because its structure follows the molecular processes and pathways identified through functional annotation and literature-supported interpretation. In addition, substantial restructuring would reduce the overall coherence between Results and the working model proposed on Figure 7.

Finally, there are a few minor methodological and visual issues that should be corrected to improve the manuscript's clarity and rigor:

The authors need to specify in the methods whether any batch correction was performed on Experiments 1 and 2, assuming these sample sets were collected at different times.

No batch correction was performed for Experiments 1 and 2. Although the sample sets were collected at different times, all samples were processed together as a single batch for RNA extraction, as well as for downstream RNA-seq library preparation and sequencing. This clarification has been added to the Materials and Methods section (Lines 778–780 and Line 803).

The data presented in Figures 3A and 3B appear to be redundant and should be consolidated or clearly differentiated.

Figure 3B corresponds to a focused view of Figure 3A intended to emphasis the statical significance of ABA-responsive modules. To avoid confusion, the Figure 3B legend was modified accordingly (“Panel B shows a focused view of Panel A”, Line 1562).
