# Supplementary material for: Gene network analysis predicts the primary regulators of ABA-dependent transcriptional activation and repression in Populus roots
Source: PLoS Genet. 2026 Aug 4;22(8):e1012256. doi: 10.1371/journal.pgen.1012256 (PMC13466054; doi:10.1371/journal.pgen.1012256)
Supplement: Peer review history

# Peer review: Decision Letter 0

**DOI:** 10.1371/journal.pgen.1012256.r001

**Authors:** Angela Hancock, Arun Sampathkumar

12 Apr 2026

PGENETICS-D-26-00159

Gene network analysis predicts the primary regulators of ABA-dependent transcriptional activation and repression in Populus roots

PLOS Genetics

Dear Dr. Cohen,

Thank you for submitting your manuscript to PLOS Genetics and thank you for your patience. After careful consideration, we feel that it has merit but does not fully meet PLOS Genetics's publication criteria as it currently stands. Therefore, we invite you to submit a revised version of the manuscript that addresses the points raised during the review process.

Please submit your revised manuscript by Jun 11 2026 11:59PM. If you will need more time than this to complete your revisions, please reply to this message or contact the journal office at plosgenetics@plos.org. When you're ready to submit your revision, log on to https://www.editorialmanager.com/pgenetics/ and select the 'Submissions Needing Revision' folder to locate your manuscript file.

Please include the following items when submitting your revised manuscript:

* A letter that responds to each point raised by the editor and reviewer(s). You should upload this letter as a separate file labeled 'Response to Reviewers'. This file does not need to include responses to any formatting updates and technical items listed in the 'Journal Requirements' section below.

* A marked-up copy of your manuscript that highlights changes made to the original version. You should upload this as a separate file labeled 'Revised Manuscript with Track Changes'.

* An unmarked version of your revised paper without tracked changes. You should upload this as a separate file labeled 'Manuscript'.

If you would like to make changes to your financial disclosure, competing interests statement, or data availability statement, please make these updates within the submission form at the time of resubmission. Guidelines for resubmitting your figure files are available below the reviewer comments at the end of this letter.

We look forward to receiving your revised manuscript.

Kind regards,

Arun Sampathkumar

Academic Editor

PLOS Genetics

Angela Hancock

Section Editor

PLOS Genetics

Aimée Dudley

Editor-in-Chief

PLOS Genetics

Anne Goriely

Editor-in-Chief

PLOS Genetics

**Journal Requirements:**

If the reviewer comments include a recommendation to cite specific previously published works, please review and evaluate these publications to determine whether they are relevant and should be cited. There is no requirement to cite these works unless the editor has indicated otherwise.

1) We ask that a manuscript source file is provided at Revision. Please upload your manuscript file as a .doc, .docx, .rtf or .tex. If you are providing a .tex file, please upload it under the item type u2018LaTeX Source Fileu2019 and leave your .pdf version as the item type u2018Manuscriptu2019.

2) Please upload all main figures as separate Figure files in .tif or .eps format. For more information about how to convert and format your figure files please see our guidelines:

https://journals.plos.org/plosgenetics/s/figures

3) In the online submission form, you indicated that  The RNA-Seq data are currently under embargo at NCBI (https://www.ncbi.nlm.nih.gov/geo/query/acc.cgi?acc=GSE183780), and access will be provided to reviewers upon request. All PLOS journals now require all data underlying the findings described in their manuscript to be freely available to other researchers, either

1. In a public repository

2. Within the manuscript itself

3. Uploaded as supplementary information.

This policy applies to all data except where public deposition would breach compliance with the protocol approved by your research ethics board. If your data cannot be made publicly available for ethical or legal reasons (e.g., public availability would compromise patient privacy), please explain your reasons by return email and your exemption request will be escalated to the editor for approval. Your exemption request will be handled independently and will not hold up the peer review process, but will need to be resolved should your manuscript be accepted for publication. One of the Editorial team will then be in touch if there are any issues.

4) Please amend your detailed Financial Disclosure statement. This is published with the article. It must therefore be completed in full sentences and contain the exact wording you wish to be published.

1) State the initials, alongside each funding source, of each author to receive each grant. For example: "This work was supported by the National Institutes of Health (####### to AM; ###### to CJ) and the National Science Foundation (###### to AM)."

2) State what role the funders took in the study. If the funders had no role in your study, please state: "The funders had no role in study design, data collection and analysis, decision to publish, or preparation of the manuscript."

3) If any authors received a salary from any of your funders, please state which authors and which funders..

If you did not receive any funding for this study, please simply state: u201cThe authors received no specific funding for this work.u201d

**Reviewers' comments:**

Reviewer's Responses to Questions

**Comments to the Authors:**

**Please note here if the review is uploaded as an attachment.**

Reviewer #1: This manuscript assayed the effect of extraneous ABA on non-stressed plant roots as a method to estimate the regulatory cascade in response to ABA without the confounding effects of other stress sensors. The authors posit that this work fills an important knowledge gap in understanding root responses to ABA, particularly in Poplar. The study was well-designed and the authors have done meticulous work in analyzing the transcriptomic data to identify the individual genes and broad trends involved in ABA response. I particularly appreciate that they compared the ABA response in this study to the osmotic stress response which helps place the pure ABA-triggered response within the broader osmotic stress faced often by plants. However, there are a few shortcomings in the way the paper is conceived and presented.

1. The authors correctly identify that there is a knowledge gap in our understanding of ABA response in roots. However, beyond the transcriptome data and the derived functional analyses the authors do not address the key question of what knowledge was gained by the design and execution of this experiment. An important component of plant response to drought stress is the change to the root system architecture, which is presumably within the purview of ABA signaling. This work would benefit from a systematic comparison of the ABA response seen here to the previously described studies on root development to define the effect of ABA responsive modules on root traits such as meristem activity, lateral root initiation and expansion etc.

2. This study was performed on roots after the removal of shoot tissue to hone in specifically on the root ABA response. The question then is what was learnt from this approach beyond what was already known from shoot/whole plant studies? Much of the results and discussion sections describe how the known regulators of ABA response (ABFs etc.) were rediscovered here. While that proves the validity of the approach it does not provide any new insights into the root response. The authors should compare and contrast the root response to shoot responses (e.g., from previous studies) to specify what portion of the root response is broadly true across all organs and which portion is specific to roots. If a set of genes are uniquely ABA responsive in roots, then what are the potential regulators of these genes that are identified by the iDREM or WGCNA approaches.

3. The results section includes multiple subsections that discuss the individual classes of genes or processes in ABA response. It is a bit tedious to discuss the effect of ABA on each process and component gene separately. The manuscript would benefit from a discussion of the significance of these changes. For example, what is the significance of repressing Tetrapyrrole and chlorophyll metabolism in roots?

Further specific comments:

1. The design of the study with experiment1 and experiment 2 is difficult to track, especially since there is no direct statement or illustration of the time points sampled. Please include in the first section discussing transcriptomic data (Lines 200-210), an explicit definition of the time points sampled. The embedded timepoint information in Fig. 3B is both too late and too difficult to decipher for the reader.

2. In lines 205-210 the authors place the point of path divergence as the earliest point before the divergence as visualized in Fig 2. It would be fairer to say that the paths diverged sometime before the next timepoint. For example, line 205 should state “Path A and B separated within 15 minutes of the start of treatment….” Again on line 209 “divergence between path D and F occurred later (between 15 and 30 min), …”

3. On lines 240 and 241, the exact sampling timepoints for ABA+ and ABA+/- should be defined. Another suggestion for clarity it to relabel the ABA+/- samples as +60 minutes and +960 minutes or some other way to differentiate them from the 60 and 960 minutes sampled in the ABA+ treatment.

4. Fig. 2C uses the red-white gradient to denote two different contexts, once for module identity and second time for significance which makes it hard to interpret this panel. Please switch to an alternate color gradient for the -log10(p-val) such as green or purple gradient.

5. Fig. 3C preserves the module number for arranging columns, which is not biologically meaningful. The panel would be much easier to interpret is the columns are grouped by the induction/repressed axis used in panel B (i.e., M1,10,4,16 followed by M2,5,3)

Reviewer #2: This paper reports a detailed time‑course analysis of the transcriptomic response that occurs in poplar root tips upon ABA treatment. Using iDREM, the authors classified the early ABA response into six groups, and through WGCNA they identified 17 modules (M1–M17). The discussion focuses on the M3 and M4 modules: M4 forms a large‑scale network centered on ABF/ABI5, which are directly regulated by the ABA core signaling pathway. The ABA‑activated network represents the core of the drought‑stress response, whereas the ABA‑repressed module (M3) integratively suppresses secondary metabolism, senescence‑related pathways, and strigolactone biosynthesis.

As the authors note, the root transcriptome is well suited for dissecting short‑term transcriptional responses to ABA. Although several studies in various plant species have reported time‑course transcriptome analyses of ABA‑induced responses, this work is unique in that it collects appropriately fine‑scaled time points and analyzes in detail how transcriptional networks are constructed from the very earliest primary responses. Conducting ABA treatments in complete darkness may also be considered relatively unique. Overall, the experiments are carefully designed, and the resulting transcriptome data appear to be very clean. Given the large volume of high‑quality transcriptomic data, the authors’ decision to focus the paper specifically on transcriptional regulation is logical and appropriate.

Below are several minor corrections:

The authors need to deposit raw transcriptome data to the public site.

Discussion (line 553): “Abscisic acid” → “ABA”

Reference list: The formatting lacks consistency and contains errors.

Reviewer #3: In this study, Cohen et al. generate time-resolved, high-resolution RNA-seq datasets from Populus roots following ABA treatment. Using these datasets, the authors identify early regulators of this process, pinpoint a potential core gene set involved in ABA-dependent stress responses, and propose a comprehensive working model for transcriptional ABA action in Populus roots. While this study provides a valuable resource to the research community, the manuscript requires substantial revision and structural improvement before it is suitable for publication.

Although the authors have generated a comprehensive co-expression network (Figures 3 and 4) and characterized the dynamics of the ABA response (Figure 2), the current analysis remains overly descriptive. Applying a more quantitative approach would greatly improve the holistic understanding of this physiological response and its major regulators. The following analytical and structural issues should be addressed:

Regarding Figure 2, the authors should clarify whether any specific transcription factor (TF) families were overrepresented. Furthermore, it is unclear what proportion of the TFs and potential target genes identified in Figure 2 are included in the co-expression network (Figure 4), and to which modules they belong. Integrating this information into a unified gene regulatory network would make these findings significantly more informative.

A similar issue occurs in the analysis of the public osmotic stress dataset. The authors generate a second co-expression network and compute the correlation between the osmotic and ABA networks (Figure 5). While these data are interesting, the current text and visualizations make it difficult to determine which modules are the most statistically significant in ABA-mediated osmotic regulation, as well as the direction of regulation for the differentially expressed genes (DEGs). The text should be revised to clearly identify the key TFs and their target genes in this context.

The Results subsection titled "Identifying the hallmarks of ABA-dependent transcriptional network" discusses the functions of numerous genes that are either activated or repressed by ABA, relying heavily on heatmaps (Figure 6 and supporting figures). If these genes were derived from the broader network, displaying them as targeted sub-networks would be much more effective. Additionally, this section lacks quantitative context (e.g., noting what percentage of genes involved in ABA feedback loops were actually recovered in the analysis). As currently written, this section reads like a Discussion; it is excessively lengthy and requires substantial reorganization and streamlining.

Finally, there are a few minor methodological and visual issues that should be corrected to improve the manuscript's clarity and rigor:

The authors need to specify in the methods whether any batch correction was performed on Experiments 1 and 2, assuming these sample sets were collected at different times.

The data presented in Figures 3A and 3B appear to be redundant and should be consolidated or clearly differentiated.

**********

**Have all data underlying the figures and results presented in the manuscript been provided?**

Large-scale datasets should be made available via a public repository as described in the *PLOS Genetics*  data availability policy, and numerical data that underlies graphs or summary statistics should be provided in spreadsheet form as supporting information.

Reviewer #1: Yes

Reviewer #2: Yes

Reviewer #3: None

**********

PLOS authors have the option to publish the peer review history of their article (what does this mean?). If published, this will include your full peer review and any attached files.

If you choose “no”, your identity will remain anonymous but your review may still be made public.

**Do you want your identity to be public for this peer review?**  For information about this choice, including consent withdrawal, please see our Privacy Policy.

Reviewer #1: **Yes:** Kranthi Varala

Reviewer #2: **Yes:** Eiji Nambara

Reviewer #3: No

**Figure resubmission:**

While revising your submission, we strongly recommend that you use PLOS’s NAAS tool (https://ngplosjournals.pagemajik.ai/artanalysis) to test your figure files. NAAS can convert your figure files to the TIFF file type and meet basic requirements (such as print size, resolution), or provide you with a report on issues that do not meet our requirements and that NAAS cannot fix.

After uploading your figures to PLOS’s NAAS tool - https://ngplosjournals.pagemajik.ai/artanalysis, NAAS will process the files provided and display the results in the "Uploaded Files" section of the page as the processing is complete. If the uploaded figures meet our requirements (or NAAS is able to fix the files to meet our requirements), the figure will be marked as "fixed" above. If NAAS is unable to fix the files, a red "failed" label will appear above. When NAAS has confirmed that the figure files meet our requirements, please download the file via the download option, and include these NAAS processed figure files when submitting your revised manuscript.

**Reproducibility:**

To enhance the reproducibility of your results, we recommend that authors of applicable studies deposit laboratory protocols in protocols.io, where a protocol can be assigned its own identifier (DOI) such that it can be cited independently in the future. Additionally, PLOS ONE offers an option to publish peer-reviewed clinical study protocols. Read more information on sharing protocols at https://plos.org/protocols?utm_medium=editorial-email&utm_source=authorletters&utm_campaign=protocols

# Author comment: Author response to Decision Letter 1

**DOI:** 10.1371/journal.pgen.1012256.r002

8 Jun 2026

Attachment Submitted filename: Response to Reviewers.docx

# Peer review: Decision Letter 1

**DOI:** 10.1371/journal.pgen.1012256.r003

**Authors:** Angela Hancock, Arun Sampathkumar

15 Jul 2026

Dear Dr Cohen,

We are pleased to inform you that your manuscript entitled "Gene network analysis predicts the primary regulators of ABA-dependent transcriptional activation and repression in Populus roots" has been editorially accepted for publication in PLOS Genetics. Congratulations!

We apologize for the delay in this decision; unfortunately, we were unable to secure feedback from the third reviewer in a timely manner. Thank you, however, for thoroughly addressing all the comments from the previous round of reviews.

Before your submission can be formally accepted and sent to production you will need to complete our formatting changes, which you will receive in a follow up email. Please be aware that it may take several days for you to receive this email; during this time no action is required by you. Please note: the accept date on your published article will reflect the date of this provisional acceptance, but your manuscript will not be scheduled for publication until the required changes have been made.

Once your paper is formally accepted, an uncorrected proof of your manuscript will be published online ahead of the final version, unless you’ve already opted out via the online submission form. If, for any reason, you do not want an earlier version of your manuscript published online or are unsure if you have already indicated as such, please let the journal staff know immediately at plosgenetics@plos.org.

In the meantime, please log into Editorial Manager at https://www.editorialmanager.com/pgenetics/, click the "Update My Information" link at the top of the page, and update your user information to ensure an efficient production and billing process. Note that PLOS requires an ORCID iD for all corresponding authors. Therefore, please ensure that you have an ORCID iD and that it is validated in Editorial Manager. To do this, go to ‘Update my Information’ (in the upper left-hand corner of the main menu), and click on the Fetch/Validate link next to the ORCID field.  This will take you to the ORCID site and allow you to create a new iD or authenticate a pre-existing iD in Editorial Manager.

If you have a press-related query, or would like to know about making your underlying data available (as you will be aware, this is required for publication), please see the end of this email. If your institution or institutions have a press office, please notify them about your upcoming article at this point, to enable them to help maximise its impact. Inform journal staff as soon as possible if you are preparing a press release for your article and need a publication date.

Thank you again for supporting open-access publishing; we are looking forward to publishing your work in PLOS Genetics!

Yours sincerely,

Arun Sampathkumar

Academic Editor

PLOS Genetics

Angela Hancock

Section Editor

PLOS Genetics

Aimée Dudley

Editor-in-Chief

PLOS Genetics

Anne Goriely

Editor-in-Chief

PLOS Genetics

www.plosgenetics.org

BlueSky: @plos.bsky.social

----------------------------------------------------

Comments from the reviewers (if applicable):

Reviewer's Responses to Questions

**Comments to the Authors:**

**Please note here if the review is uploaded as an attachment.**

Reviewer #1: Thanks to the authors for revising the updated and improved manuscript. The new version addresses all my concerns and I am happy to recommend it for publication.

Reviewer #2: The authors have revised the manuscript correctly. I have no further comments.

**********

**Have all data underlying the figures and results presented in the manuscript been provided?**

Large-scale datasets should be made available via a public repository as described in the *PLOS Genetics*  data availability policy, and numerical data that underlies graphs or summary statistics should be provided in spreadsheet form as supporting information.

Reviewer #1: Yes

Reviewer #2: Yes

**********

PLOS authors have the option to publish the peer review history of their article (what does this mean?). If published, this will include your full peer review and any attached files.

If you choose “no”, your identity will remain anonymous but your review may still be made public.

**Do you want your identity to be public for this peer review?**  For information about this choice, including consent withdrawal, please see our Privacy Policy.

Reviewer #1: **Yes:** Kranthi Varala

Reviewer #2: No

----------------------------------------------------

**Data Deposition**

If you have submitted a Research Article or Front Matter that has associated data that are not suitable for deposition in a subject-specific public repository (such as GenBank or ArrayExpress), one way to make that data available is to deposit it in the Dryad Digital Repository. As you may recall, we ask all authors to agree to make data available; this is one way to achieve that. A full list of recommended repositories can be found on our website.

The following link will take you to the Dryad record for your article, so you won't have to re‐enter its bibliographic information, and can upload your files directly:

http://datadryad.org/submit?journalID=pgenetics&manu=PGENETICS-D-26-00159R1

More information about depositing data in Dryad is available at http://www.datadryad.org/depositing. If you experience any difficulties in submitting your data, please contact help@datadryad.org for support.

Additionally, please be aware that our data availability policy requires that all numerical data underlying display items are included with the submission, and you will need to provide this before we can formally accept your manuscript, if not already present.

----------------------------------------------------

**Press Queries**

If you or your institution will be preparing press materials for this manuscript, or if you need to know your paper's publication date for media purposes, please inform the journal staff as soon as possible so that your submission can be scheduled accordingly. Your manuscript will remain under a strict press embargo until the publication date and time. This means an early version of your manuscript will not be published ahead of your final version. PLOS Genetics may also choose to issue a press release for your article. If there's anything the journal should know or you'd like more information, please get in touch via plosgenetics@plos.org.

# Editor report: Acceptance letter

**DOI:** 10.1371/journal.pgen.1012256.r004

**Authors:** Angela Hancock, Arun Sampathkumar

PGENETICS-D-26-00159R1

Gene network analysis predicts the primary regulators of ABA-dependent transcriptional activation and repression in Populus roots

Dear Dr Cohen,

We are pleased to inform you that your manuscript entitled "Gene network analysis predicts the primary regulators of ABA-dependent transcriptional activation and repression in Populus roots" has been formally accepted for publication in PLOS Genetics! Your manuscript is now with our production department and you will be notified of the publication date in due course.

The corresponding author will soon be receiving a typeset proof for review, to ensure errors have not been introduced during production. Please review the PDF proof of your manuscript carefully, as this is the last chance to correct any errors. Please note that major changes, or those which affect the scientific understanding of the work, will likely cause delays to the publication date of your manuscript.

Soon after your final files are uploaded, unless you have opted out or your manuscript is a front-matter piece, the early version of your manuscript will be published online. The date of the early version will be your article's publication date. The final article will be published to the same URL, and all versions of the paper will be accessible to readers.

For Research Articles, you will receive an invoice from PLOS for your publication fee after your manuscript has reached the completed accept phase. If you receive an email requesting payment before acceptance or for any other service, this may be a phishing scheme. Learn how to identify phishing emails and protect your accounts at https://explore.plos.org/phishing.

Thank you again for supporting PLOS Genetics and open-access publishing. We are looking forward to publishing your work!

With kind regards,

Kannan R K Kuppusamy, B.TECH BIOTECHNOLOGY

PLOS Genetics

On behalf of:

The PLOS Genetics Team

Carlyle House, Carlyle Road, Cambridge CB4 3DN | United Kingdom

plosgenetics@plos.org | +44 (0) 1223-442823

plosgenetics.org | Twitter: @PLOSGenetics
